# Selection for avian leukosis virus integration sites determines the clonal progression of B-cell lymphomas

**DOI:** 10.1371/journal.ppat.1006708

**Published:** 2017-11-03

**Authors:** Sanandan Malhotra, Shelby Winans, Gary Lam, James Justice, Robin Morgan, Karen Beemon

**Affiliations:** 1 Department of Biology, Johns Hopkins University, Baltimore, Maryland, United States of America; 2 Department of Biological Sciences, University of Delaware, Newark, Delaware, United States of America; Imperial College London, UNITED KINGDOM

## Abstract

Avian leukosis virus (ALV) is a simple retrovirus that causes a wide range of tumors in chickens, the most common of which are B-cell lymphomas. The viral genome integrates into the host genome and uses its strong promoter and enhancer sequences to alter the expression of nearby genes, frequently inducing tumors. In this study, we compare the preferences for ALV integration sites in cultured cells and in tumors, by analysis of over 87,000 unique integration sites. In tissue culture we observed integration was relatively random with slight preferences for genes, transcription start sites and CpG islands. We also observed a preference for integrations in or near expressed and spliced genes. The integration pattern in cultured cells changed over the course of selection for oncogenic characteristics in tumors. In comparison to tissue culture, ALV integrations are more highly selected for proximity to transcription start sites in tumors. There is also a significant selection of ALV integrations away from CpG islands in the highly clonally expanded cells in tumors. Additionally, we utilized a high throughput method to quantify the magnitude of clonality in different stages of tumorigenesis. An ALV-induced tumor carries between 700 and 3000 unique integrations, with an average of 2.3 to 4 copies of proviral DNA per infected cell. We observed increasing tumor clonality during progression of B-cell lymphomas and identified gene players (especially *TERT* and *MYB*) and biological processes involved in tumor progression.

## Author summary

The Avian Leukosis Virus (ALV) is a simple retrovirus that causes cancer in chickens. The virus integrates its genome into the host genome and induces changes in expression of nearby genes. Here, we determine the sites of viral integrations and their role in the progression of tumors. We report pathways and novel gene players that might cooperate and play a role in the progression of B-cell lymphomas. Our study provides new insights into the changes during lymphoma initiation, progression, and metastasis, as a result of selection for specific ALV integration sites.

## Introduction

Avian leukosis virus (ALV) is a simple retrovirus that causes cancer, primarily B-cell lymphomas in chickens [1–3]. The ALV genome does not contain a viral oncogene and induces aberrant host gene expression via use of strong viral enhancer and promoter elements. Relative to other well studied retroviruses like HIV-1 and MLV, ALV was shown to integrate relatively randomly into the host genomic DNA, with little bias for genomic features [4–7]. The FACT (facilitates chromatin transcription) complex, a chromatin remodeler, was recently reported to promote ALV integration [8].

ALV-induced lymphomas develop in a multistage process, appearing initially as neoplastic follicles in the bursa, some of which develop into primary bursal tumors. Primary tumors can then metastasize to distant organs and form secondary tumors [9]. We are studying rapid-onset lymphomas, which develop in less than 3 months after infection of chicken embryos with ALV [10,11].

Cellular transformation occurs through multiple genetic changes in oncogenes and tumor suppressor genes, as well as noncoding RNAs [12]. These oncogenic changes can occur via different genetic mechanisms; insertional mutagenesis by retroviruses is one such mechanism. Viral integration into the host genome can alter host gene expression and induce cancer development [1–3]. In turn, common viral integration targets observed in multiple tumors can help identify oncogenes [2,13–19]. Consequently, retroviral-mediated lymphomagenesis in chickens provides an excellent experimental model system for analysis of neoplastic change in tumors of B-cell lineage [20]. We have previously identified common proviral integrations in ALV-induced B-cell lymphomas, notably in the *TERT* promoter region, and in hemangiomas [15–17].

Since selection in tumorigenesis alters the pattern of viral integration sites, we also analyze integrations in cultured cells, to identify preferences of ALV integrations in an unbiased way. We investigate how ALV integrations in tissue culture correlate with previously unreported genomic features. We observe an enrichment of ALV integrations in the 5’ end of gene bodies, proximal to CpG islands and transcription start sites, as well as a preference for expressed and highly spliced genes. No association was observed with levels of alternative splicing. In order to determine the effects of selection for oncogenic characteristics, we compare integration sites in cultured cells with those in ALV-induced B-cell lymphomas. We observe that ALV integrations are more strongly selected for proximity to transcription start sites in tumors. In addition, there are fewer integrations near CpG islands in tumors.

ALV tumors are thought to be clonal, as determined by previous work [9,15]. However, the clonality of these neoplasms has not been empirically defined. We analyze ALV infection in tumors by quantifying the clonal abundance and distribution of integrations during progression of tumors. Using the statistical Gini index, we calculate the empirical degree of oligoclonality and extent of clonal expansion in different stages of tumorigenesis [21,22]. Quantifying the clonality index and average number of integrations per cell within individual tumors helps determine the clonal architecture and hierarchies of lymphomagenesis. Furthermore, the gene ontology analysis of host genes most proximal to proviral sites provides insight into underlying gene players and their contribution to oncogenic transformation. Thus, our work helps unravel how the integration sites of ALV are selected for in oncogenesis and play a role in the clonal progression of tumors. This is the most in depth analysis for ALV integration sites to date and is novel in terms of being able to follow the patterns of integration from early infection (in tissue culture) through to early and late tumor development.

## Results

### ALV integration is enriched within genes in cultured cells and in tumors

Via deep sequencing, we analyzed approximately 87,000 unique ALV integration sites (UISs) in tissue culture cells and in tumors, as summarized in S1 Table. Randomly generated integration sites were used as a control for all subsequent analysis. ALV integrations were analyzed for different ALV subgroups (A, C and J) in different infected cell types, including primary chicken embryo fibroblasts (CEF), DT-40, a chicken B-cell lymphoma cell line, and the human HeLa tumor cell line. After mapping the UISs to the host genome, we used the HOMER bioinformatics tool [23] to associate integrations with specific annotated genomic sites (Table 1).

**Table 1:** ALV integrations analyzed in tissue culture and tumors for association with different genome features.

Genome Feature	Random (Galgal 4) (%)	ALV-A CEF (%)	ALV-C CEF (%)	ALV-J CEF (%)	ALV-C DT-40 (%)	ALV-A Tumors (%)	Random (hg19) (%)	ALV-C HeLa (%)
+/- 5kb of TSS	6	8.6	10.9	8.3	9.8	15.4	7.9	13.7
TTS	0.7	0.8	0.9	0.5	1	1.1	0.9	1.3
LINE	6.8	9.9	9.3	7.8	10.7	7.5	7.2	7.8
SINE	0.2	0.2	0.1	0.1	0	0.1	0.1	0.1
Promoter	0.6	1.1	1.2	0.8	0.6	1.4	0.5	1.2
CpG Island	1.1	3.9	2.7	2.3	2.5	1.4	1.2	3.1
Low complexity	0.6	0.4	0.2	0.3	0.3	0.3	0.5	0.2
Simple repeat	0.6	0.7	2.1	5.2	1.5	1.9	0.8	1.9
Satellite	0.3	1.9	3.4	1.4	4.5	1.2	0.5	0.9
Genes	27	38	38.7	41.1	40	37.6	25.4	36.3
Intergenic	73	62	61.3	58.9	59.6	62.4	74.6	63.7

HOMER bioinformatics analysis was performed for infections with different ALV subgroups (A, C and J) in tissue culture (CEF, DT-40 and HeLa cells) and tumors. Percentage of integrations for each category is depicted. As a control, analysis was repeated for a matched number of random sites in the chicken genome (Galgal 4) or human genome (hg19).

**Fig 1:**
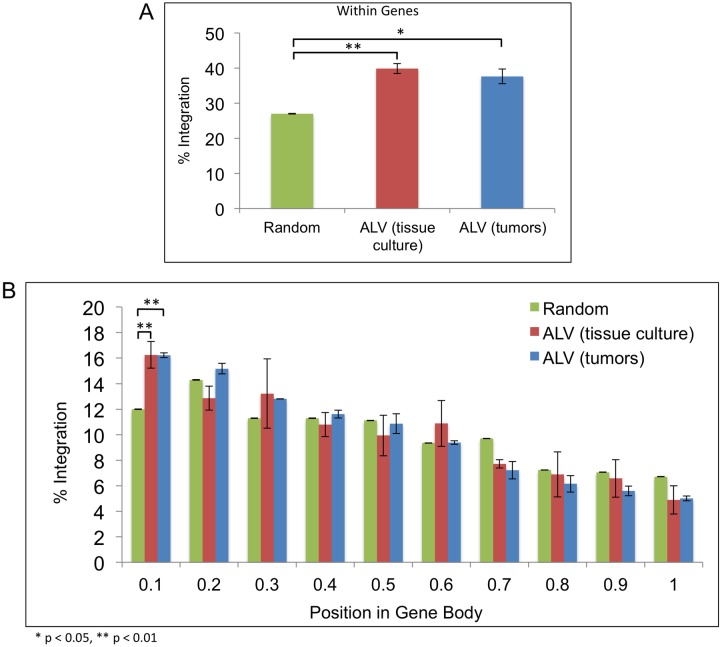
Distribution of ALV integration sites in genes. (A) The percentage of ALV integration events within gene bodies (transcription start site to termination site) was determined in tissue culture and in tumors, relative to a control number of random events, using the ensembl *Gallus gallus* 4 genome. (B) Each transcription unit was divided into 10 equal bins beginning from the 5’ end of the transcription start site. The percentages of ALV integrations were calculated for each bin. Random integration events were used as a relative control.

Upon analysis of ALV integrations in CEFs, using the ensembl *Gallus gallus* 4 genome, we observed a significant bias for ALV integration into genes (approximately 40%) relative to random events (27%) (*t* test, p-value 0.006) (Fig 1A). We also observed a slight enrichment for integrations near LINE sequences, gene promoters, simple repeat and satellite DNA sequences; however, these were not statistically significant (Table 1). Independent analysis of all these features was consistent for infections with ALV in CEFs, DT-40 chicken B cells and in HeLa cells, suggesting that these preferences are not cell type specific (Table 1).

To study selection of ALV integration sites in tumors, we sequenced 72 tissue samples from 41 different birds (S2 Table). We obtained 17.2 million reads, originating from viral integrations in neoplasms and non-tumor tissues, which were mapped to 71,368 UISs. Similar to the integration pattern in cultured cells, integrations in the ALV-induced tumors, at the primary sites in the bursa or secondary metastases, showed a significant enrichment for genes (approximately 38%), relative to random (27%) (*t* test, p-value 0.022) (Fig 1A, Table 1).

### ALV integrations are enriched at the 5’ end of gene bodies in cultured cells and in tumors

We analyzed the distribution of ALV integrations within transcriptional units by dividing the gene bodies into 10 equal segments or bins. Then, we calculated the number of integrations within each bin to determine the density of ALV integrations within a given part of a transcriptional unit. Relative to the matched control set (11.99%), there was a significant enrichment of ALV integrations towards the 5’ end of the gene body. This bias is most distinct within the first 10% of the gene body, i.e. in proximity to the transcriptional start site, in both tissue culture (16.25%) (*t* test, p-value 0.031), and in tumors (16.22%) (*t* test, p-value 0.004) (Fig 1B).

### ALV integration near transcription start sites and CpG islands varies in cultured cells versus tumors

**Fig 2:**
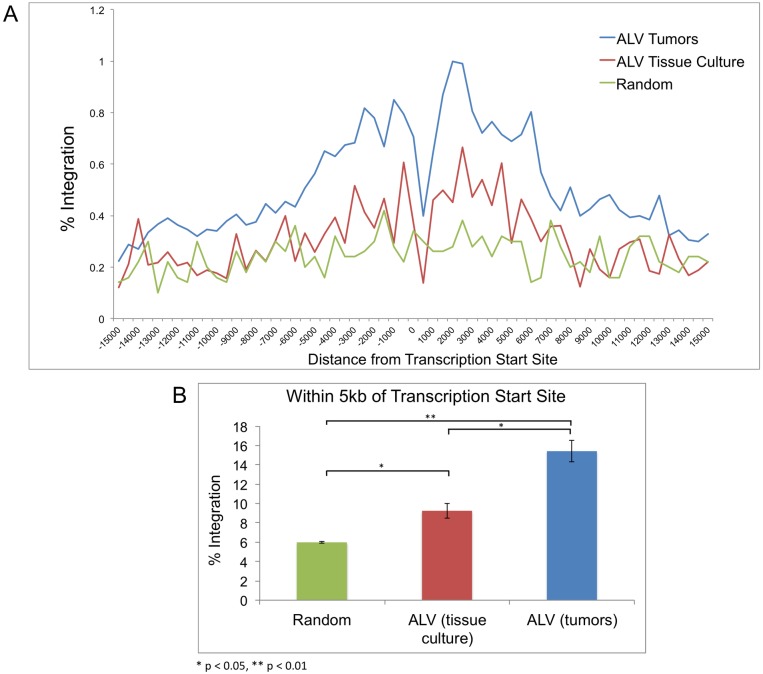
Distribution of ALV integrations with respect to transcription start sites. ALV integrations in tissue culture (n = 15,416) and in tumors (n = 71,368) were compared to a control number of random integration events in the ensembl *Gallus gallus* 4 genome. (A) A plot for the percentage of integration events within a 30 kb window of transcription start sites (TSS) is shown, with division into 500-bp bins. The blue and red lines represent ALV integrations in tumors and tissue culture respectively, and the green line represents the random control events. A preference for integration around TSSs is observed. However, a striking lack of integrations was observed in the immediate vicinity of TSSs. (B) Percentage of integrations within 5 kb of the TSS are calculated for ALV integrations in culture and tumors versus random data.

To determine the pattern of integrations surrounding transcription start sites (TSS), we plotted the observed ALV integrations in cultured cells and in tumors with respect to the nearest TSS, extending over 15 kb on either end (Fig 2A). Relative to the random integration sites within 5 kb of the TSS, we observed a nearly 2-fold enrichment of ALV integration sites in tissue culture (*t* test, p-value 0.042) (Fig 2B). We observed an even greater frequency of integrations near TSS in tumors, with nearly 3-fold enrichment relative to random events within the 5 kb window (*t* test, p-value 0.006). This suggests that clonal selection of integration sites in tumors has a strong bias for proximity to TSS, probably to promote induction of aberrant gene expression in tumorigenesis. In addition, consistent with previously reported data from our lab [17], we observed a pronounced drop in the integration frequency in the vicinity of the TSS in tumors (Fig 2A). Interestingly, we also observed this drop in tissue culture (Fig 2A), suggesting it is a result of integration preference and not due to selection in tumors.

**Fig 3:**
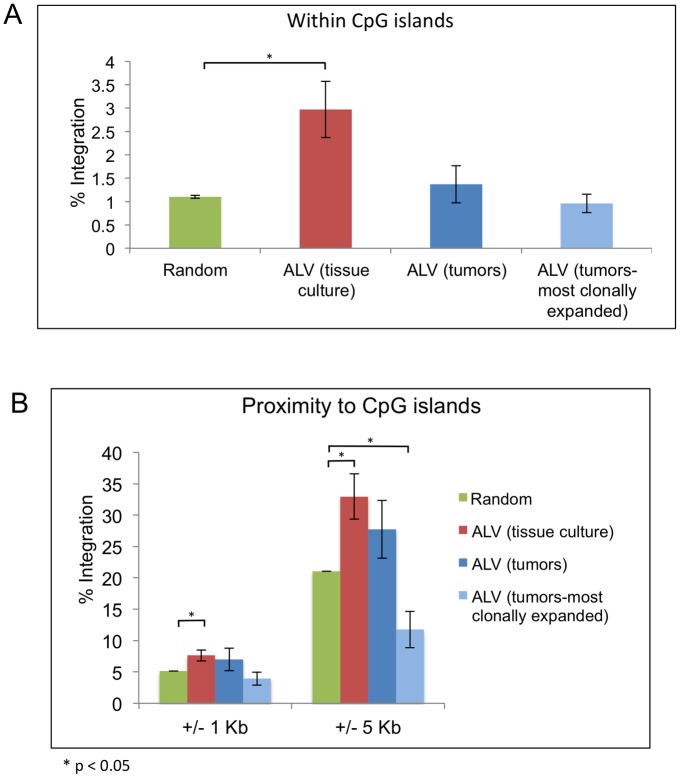
Distribution of ALV integrations in proximity to CpG islands. The percentage of ALV integration events in tissue culture and in tumors are determined for (A) within CpG islands or (B) within 1kb or 5 kb of CpG islands, relative to control number of random events. The most clonally expanded integrations in tumors include integrations with 10 or more breakpoints.

Based on our initial HOMER analysis, we observed a 3-fold preference for integrations within CpG islands (approximately 3%) relative to random sites (1.1%) in cultured cells (*t* test, p-value 0.041) (Fig 3A). In contrast to tissue culture, the percentage of integrations within CpG islands was not enriched in tumors (1.4%), and appeared near random. The same was true for the most clonally expanded integrations (0.96%), with 10 or more breakpoints (see Materials and methods). To further investigate this, we determined the frequency of ALV integrations in the area immediately surrounding CpG islands (Fig 3B). Analyzing ALV integrations in tissue culture, we found that in the 1 kb region surrounding CpG islands, there was a 1.5 fold enrichment of integration relative to random (*t* test, p-value 0.047). If the window is expanded to 5 kb flanking the CpG island, the enrichment becomes more pronounced. Nearly 33% of all integrations are observed within 5kb of CpG islands, which is a 1.7 fold enrichment relative to the matched random control (21%) (*t* test, p-value 0.043).

In contrast, this enrichment for integration near CpG islands in cultured cells was not observed in tumors (Fig 3B). Frequency of integration within 1 or 5 kb of CpG islands in tumors was not significant relative to random events. When the same analysis was done for only the most clonally expanded integrations, there is a striking depletion of integrations within 5kb of CpG islands (11.8%) relative to the total integration set in tumors (27.7%) as well as a matched random control (21%) (Fig 3B). This suggests that in tumors, there is an enrichment of integrations away from CpG islands (*t* test, p-value 0.045).

### ALV integrations are enriched in expressed transcriptional units in cultured cells and in tumors

**Fig 4:**
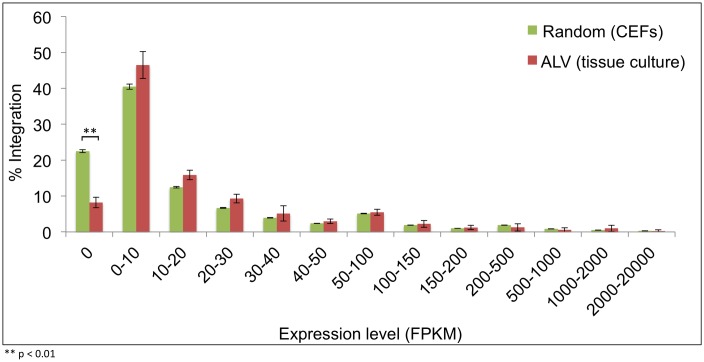
Distribution of ALV integrations relative to the expression of transcriptional units. Expression levels of the chicken RefSeq transcriptional units (6,060) in CEFs were analyzed using available RNA-seq data sets, as described in materials and methods. Gene expression levels were divided, based on FPKM (Fragments Per Kilobase of transcript per Million mapped reads) expression values, into 13 bins. Numbers of integrations that occur near or within genes were then plotted into the bins as a percentage of the total and compared to random events.

We next investigated ALV integration as a function of expression levels of the most proximal transcriptional units. In order to determine the background expression levels of host genes, we analyzed RNA-seq data for CEF, DT-40 and HeLa cells. We divided the whole transcriptome into 13 bins and determined the percentage of integrations within each bin, to observe any enrichment above background. While nearly 22.5% of the chicken genes are not expressed in CEFs, only 8.2% of integrations occur in this expression bin. Thus, we observe a significant depletion in the percentage of integrations within or in proximity to unexpressed genes (*t* test, p-value 0.006) (Fig 4). For expressed genes, there was no preference observed for ALV integration with the level of gene expression, relative to random events. Thus, ALV integrates randomly in proximity to genes with low, intermediate or high levels of expression.

Since integrations can occur in intergenic regions at distant loci from transcriptional units, we asked whether ALV integrations within the transcriptional unit might exhibit a bias towards expressed genes. To address this, we repeated our analysis for only those integrations that occur within the gene body (between the transcription start and termination sites). Overall, we observed a similar integration pattern relative to random, with a more pronounced depletion of integrations within unexpressed genes. In contrast to random events (25.4%), there was a nearly 6-fold decrease in preference for ALV integrations in genes with no expression (3.9%) (*t* test, p-value 0.003) (S1 Fig). Therefore, while ALV preferentially integrates near or within expressed genes, there is no preference for the level of gene expression.

We also correlated ALV integrations with the gene expression levels via analysis of RNA-seq data for a subset of the ALV-induced lymphomas. Similar to our findings in cultured cells, we observed that in tumors ALV integrations are selected for proximity to or within expressed genes, but there is no distinct bias for varying gene expression levels (S2 Fig).

### ALV integration is targeted to highly spliced transcriptional units

**Fig 5:**
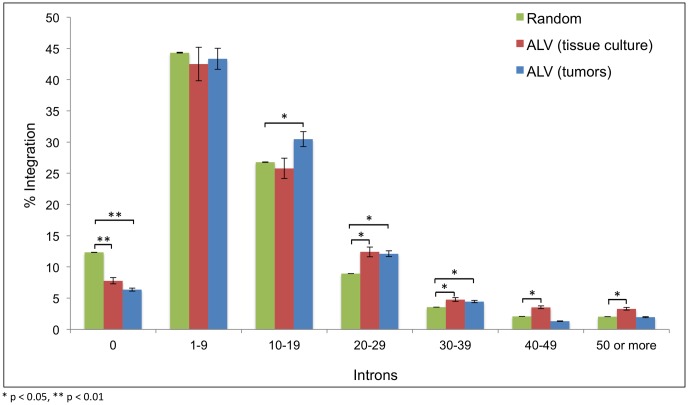
Distribution of ALV integrations relative to the level of splicing. Correlation of ALV integrations in tissue culture and in tumors with the number of introns in the transcription units is depicted, using the ensembl *Gallus gallus* 4 genome. All the transcriptional units in the chicken genome are divided into 7 bins based on their number of introns, with the first bin depicting unspliced transcripts (with 0 introns). The X-axis shows the number of introns, and the Y-axis shows the percentage of integrations that occur near or within genes with the corresponding number of introns.

HIV has been previously reported to preferentially integrate into genes that are highly spliced, i.e. have a greater number of introns [24]. In order to determine whether ALV might display any similar preferences, we correlated ALV integration with the levels of mRNA splicing and alternative splicing. We associated the percent of integrations with the number of introns of the most proximal transcriptional unit, using the ensembl *Gallus gallus* 4 genome. While by random chance 12.1% of integrations are predicted to occur in unspliced transcriptional units, only 7.7% (*t* test, p-value 0.004) and 6.4% (*t* test, p-value 0.002) of ALV integrations in cultured cells and tumors, respectively, occurred within this range. Therefore, there is a significant lack of integration into unspliced genes in tumors. By random chance, the vast majority of the integrations are predicted to occur in transcriptional units with 1–19 introns (71.1%). For this window of splicing, ALV integrations in tissue culture (68.3%) appear close to random as well. On the other hand, nearly 22.1% of ALV integrations in tissue culture fall into highly spliced genes with 20 or more introns, which is a significant enrichment above random events (16.6%) (*t* test, p-value 0.012). Furthermore, ALV integrations in tumors (47.1%) have a greater enrichment for integration within or proximity to transcriptional units with 10 to 39 introns, relative to random sites (39.3%) (*t* test, p-value 0.026). Thus, we observe that ALV has a bias for integration into spliced genes with enrichment for higher levels of gene splicing (Fig 5).

We also asked whether the levels of ALV integration are associated with the number of spliced isoforms of the proximal transcription unit. The chicken genome is not well characterized for reporting alternative spliced variants of genes. Since the human genome is better characterized than the chicken genome, we repeated our analysis for ALV integrations in a HeLa cell line (S3 Fig). However, our analysis did not identify any preference for integrations relative to the level of alternative splicing of proximal transcriptional units.

### Distribution of ALV integrations in B-cell lymphomas

**Fig 6:**
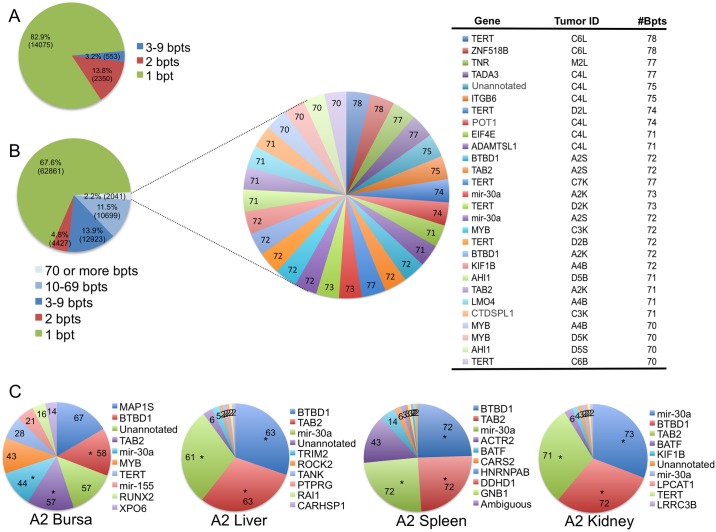
Distribution of integration sites in ALV-induced B-cell lymphomas and clonal expansion in metastases. (A) The pie chart depicts a total of 16,978 breakpoints that were identified in tissue culture (CEFs, DT-40s and HeLa cells). The single breakpoints (82.9%) and the expanded breakpoints (17.1%) are highlighted as separate slices of the pie. (B) A total of 92,951 breakpoints were identified in 69 different ALV-induced neoplasms, from 41 different birds. Genes in proximity to integrations with 70 or more breakpoints are highlighted in a separate pie. Each slice represents a unique integration site, and the size of the slice represents the number of breakpoints for that site. The table shows integrations with the highest number of breakpoints along with the tumor in which that integration was identified. The tumor ID defines the bird number (A1, B1, C2, D2 etc.) along with the respective tissue of bursa (B), liver (L), kidney (K) or spleen (S) harboring the integration. (C) A sample set of individual primary (bursa) and secondary (liver, spleen and kidney) tumors from the same bird are illustrated. The top 10 most clonally abundant UISs are depicted within each pie chart, with the corresponding number of sonication breakpoints indicated on each slice. The list of the most proximal host genes is denoted next to the pie chart. UISs observed in multiple tumors of the same bird are denoted with an “*”. While, the primary tumor in the bursa exhibits heterogeneous distribution for clonally expanded UISs, the metastasized neoplasms exhibit a more homogenous distribution of expanded clones.

In order to measure the relative abundance, or clonal expansion, of UISs within a tissue, we quantified the number of sonication breakpoints for each site, as described previously [16]. The number of breakpoints reveals the extent of clonal expansion of the UIS. In tissue culture (2–5 days after infection) we identified **16,978** unique sonication breakpoints, i.e. an average of 1.1 breakpoints per integration site (Fig 6A). The vast majority of these integrations (82.9%) had a single breakpoint, suggesting that these integrations were not clonally expanded. On the other hand, in tumors we identified 92,951 unique sonication breakpoints. The average number of breakpoints per integration was 1.3, with the vast majority of integrations (67.6%) showing only a single sonication breakpoint. In contrast to 17.1% of integrations in tissue culture, 32.3% of integrations in tumors had two or more breakpoints, revealing that a large fraction of the infected cells are from expanded clones. Moreover, a large number of integrations (approximately 13,000) in tumors had a very high number (10 or more) of breakpoints.

The distribution of viral integration sites in the neoplasms is depicted in a composite pie chart (Fig 6B). The most highly expanded clones, each with 70 or more breakpoints, are highlighted in a table. The table depicts 28 UISs, indicating the gene most proximal to the integration site, respective tumor, and its corresponding number of breakpoints. The maximum observable number of breakpoints is limited by the length of deep sequencing reads and, in the case of highly abundant integration sites, the probability of repeated sonication at the same genomic position. Thus, it is important to note that our standard breakpoint analysis is an underestimate of the fraction of the infected cells with expanded clones [21].

### ALV integrations are most clonal in metastases

Cancers exist in a number of stages, characterized by a spectrum of divergent cells and genetic changes. Each cancer is unique, and in any given tumor the clonal structure shifts over time, which involves the clonal selection and expansion of cells. [25]. In cases of ALV-induced B-cell lymphomas, the bursa serves as the primary organ of malignant transformation and site of tumorigenesis [9,26]. Infected chickens typically develop multiple primary neoplastic follicles in the bursa, some of which may eventually form primary tumors. The development of these neoplasms is a multi-stage process [26–28]. In order to examine the clonality of lymphomagenesis, we studied different stages of cancer progression. These include inflammation, neoplastic follicles, primary tumors in the bursa, and metastatic tumors at secondary sites. The stage of neoplastic follicles in the bursa is an early step in tumor progression towards transformation and malignancy [26]. Metastases of primary tumors are observed from the bursa to secondary sites in the liver, spleen, and kidneys.

Analyzing the different neoplasms, we observed an increasing extent of clonality with the advancing stages of tumorigenesis (S4 Fig). This clonal expansion can be represented by a pie chart, where each pie represents an individual tumor. A given slice of a pie represents a UIS, and the size of the slice corresponds to its relative clonal abundance, denoted by the respective number of sonication breakpoints for that UIS.

The most clonally expanded integrations in secondary tumors can be investigated by comparison of metastasized versus non-metastasized neoplasms within an individual bird (Fig 6C). For example, the different tumors in bird A2, all harbor overlapping UISs with an increasing level of clonal expansion in the metastasized neoplasms. For example, the expanded clones of UISs at *TAB2*, *BTBD1* and *mir-30a* integrations appear in a mix of other clonally expanded integrations in the primary bursa tumor, whereas the secondary liver, kidney and spleen tumors are more homogenous. This suggests increasing clonal homogeneity of the metastasized tumors relative to the bursa. Additional examples of comparisons between primary and secondary neoplasms within an individual bird are depicted in S5 Fig.

### ALV clonality increases with progressing stages of lymphomagenesis

**Fig 7:**
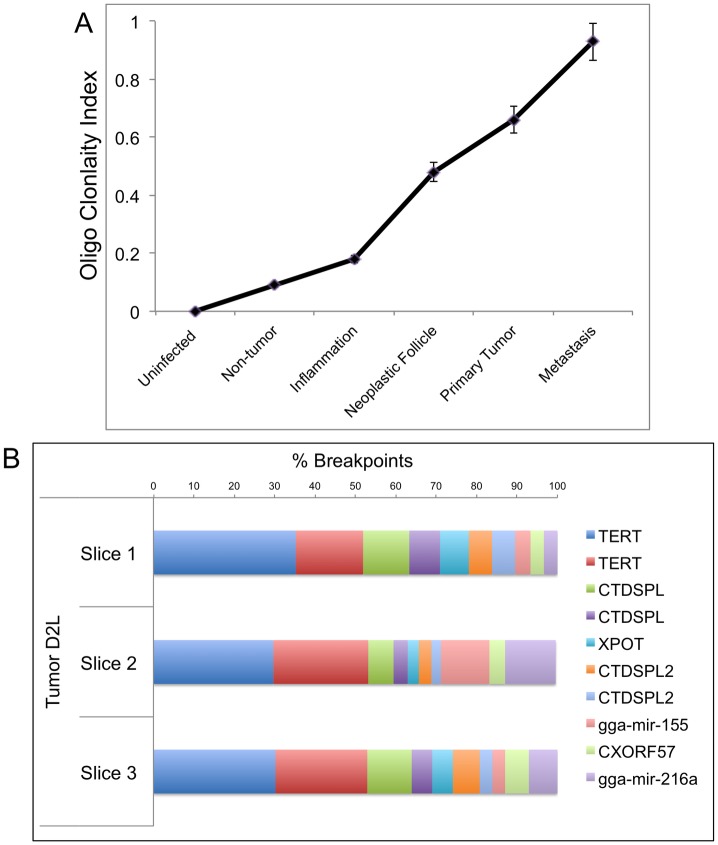
Oligoclonality Index suggests metastatic tumors are clonal. (A) The OCI values of the different tissue samples are plotted, which provide an empirical estimate of homogeneity. The OCI values range between 0.03 to 0.96. The increasing OCI values for later stages of tumorigenesis suggest increasing tissue homogeneity. (B) Top 10 most clonally expanded integrations for metastasized liver tumor D2L, are illustrated. Individual bars represent UISs and the corresponding extent of clonal expansion (as breakpoints) from 3 different parts of the same neoplasm. Slices were chosen randomly from three distinct portions of tumor mass, including peripheral and interior regions.

In order to empirically estimate the clonality of different neoplasms we made use of an objective parameter called the oligoclonality index (OCI) as defined by the Gini co-efficient index [21,29]. The OCI defines the clonal abundance of a tissue on an objective scale of 0 to 1. In theory, a tissue with perfect monoclonality would have an OCI value of 1. Conversely, an entirely polyclonal tissue would have an OCI value of 0. The OCI values of the neoplastic subtypes, in representative stages of lymphomagenesis, are depicted in Fig 7A. The plot represents the OCI values in ascending order with tumor progression, suggesting an increased magnitude of clonal expansion within these neoplasms. As was previously depicted by the pie charts of bursal tumors (Fig 6C, S4 Fig), the OCI value is lower in these samples compared to the metastases. The OCI for the metastasized tumors was significantly greater, in some cases close to 1.

Additionally, in order to further validate the OCI values, we investigated the ALV integrations and their extent of clonal expansion in different slices of representative neoplasms (Fig 7B). A sample with high clonal homogeneity would exhibit a highly uniform pattern of integrations and corresponding clonal abundance in different slices of the tumor, as depicted for tumor D2L. Conversely, a sample with lower OCI value such as a bursa tumor exhibited more clonal heterogeneity in different portions of the neoplasm (S6 Fig). Additional data for integration patterns in slices of other neoplasms is summarized in S6 Fig.

### Proviral load (PVL) increases with the progressing stages of tumorigenesis

**Fig 8:**
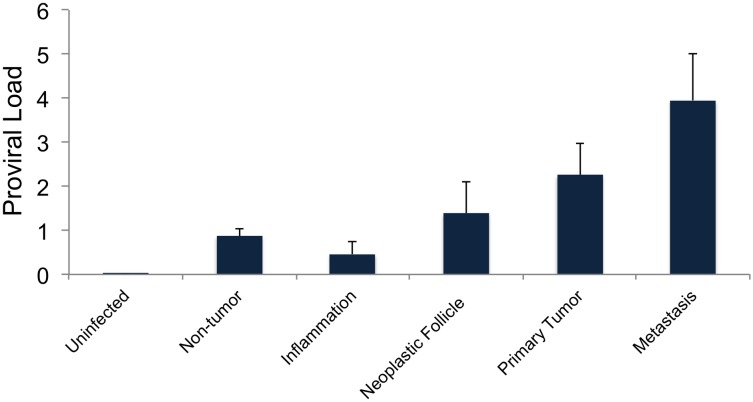
Proviral load increases with tumor progression. PVL values are represented for different malignant and non-malignant tissues, including uninfected normal tissue as control. PVL ranges between 0.5 to 4 copies per cell, with an increasing trend for later stages of tumorigenesis.

In addition to the extent of clonal expansion, we also wanted to investigate the average number of proviral integrations within tumors, defined as its proviral load (PVL). Twenty randomly selected tumors across different stages of lymphomagenesis were isolated for this analysis. The PVL varies widely between the tissues, ranging on an average from approximately 2.3 to 4 copies per infected cell of a tumor (Fig 8). We observed a correlation between the PVL and different stages of tumor progression, suggesting that a higher PVL is associated with later stages of tumorigenesis.

ALV *env* genes are more distinct and offer greater specificity to distinguish ALV proviral genomes from endogenous retrovirus genomes in the host chicken genome. However, proviruses undergo varying amounts of genome rearrangements and deletions during cellular transformation and oncogenesis, especially in the *env* region, probably to evade cellular immune surveillance. Therefore, we utilized LTR specific primers to estimate the PVL. We assume each provirus contains 2-LTRs therefore, the PVL values described here may be underestimates of the actual PVL among infected neoplasms due to the possibility of solo LTRs. Additional analyses of PVL estimates, via use of *gag* or *env* regions of ALV, exhibit similar association of PVL with the progression of tumors (S7 Fig).

### Ontologies of genes near ALV integrations highlight biological processes associated with tumor progression

**Fig 9:**
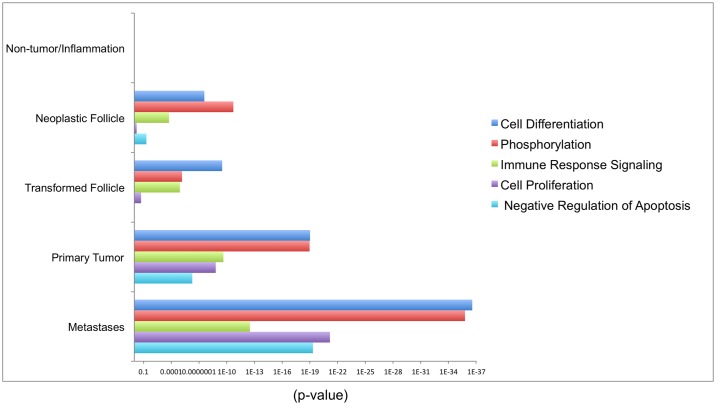
Functional classifications overrepresented among the clonally expanded integrations. Five functional categories that are significantly overrepresented among the neoplastic follicles, primary tumors and metastases, are illustrated as a bar graph. The horizontal bars represent statistical significance as defined by −log (p-values). The integrations are analyzed by g:profiler software using a random list of integrations (n = 5000) as background. Since there are no overrepresented pathways involving the integration sites in inflammation or non-tumor samples, the bars are not visible. Due to use of varying sample size, all the analysis was adjusted for multiple testing with unequal sample size using the Mann Whitney U test.

Cancers acquire, via mutational and epigenetic changes, a variety of traits that trigger clonal expansion, via proliferation, migration and invasion. These properties are alterations to normal developmental and physiological cellular processes [25]. We wanted to further investigate how ALV integrations near host genes, in certain functional categories, confer oncogenic advantages on the infected B-cell clone. To test this, we used the G:profiler analysis software to analyze the ontology of the nearest host gene upstream or downstream of each integration site. G:profiler allows functional profiling of genes within a neoplasm, in a quantitative fashion [30]. Queries are ordered with more importance given to the more expanded clones within tumors. The results showed a significant overrepresentation of genes in five cellular pathways: cell differentiation, phosphorylation, immune response signaling, proliferation and regulation of apoptosis (Fig 9). These biological processes show a greater enrichment in the secondary tumors (malignant) than in neoplasms with low or intermediate clonality.

These changes reflect the temporal order of genetic alterations and underlying cellular processes acquired in the progression of B-cell lymphomagenesis. These processes were associated with the host gene nearest to the viral integration sites, regardless of its transcriptional orientation relative to the provirus. The genes associated with these processes are listed in S3 Table. Furthermore, when we analyzed the GO terms associated with the most clonally expanded integrations in individual tumors, the above mentioned biological processes appeared repeatedly in many tumors (S4 Fig). This suggests that the gene players involved in these biological processes exhibit a degree of cooperativity to trigger oncogenic transformation. For example, among the most clonally expanded UISs in Fig 6C, *TAB2* is known to be involved in immune response signaling, *BTBD1* plays a role in cellular differentiation and *mir-30a* has known roles in regulating cell proliferation, migration and invasion [31–33].

Furthermore, we identified *TERT* and *MYB* among the most clonally expanded integrations in 15 independent tumors from 9 different birds, and in 15 independent tumors from 11 different birds, respectively (S4 Fig, S4 Table). TERT, the catalytic component of telomerase, has known roles in immortalization, senescence and apoptotic signaling [34]. MYB, a transcription factor, functions in regulation of cellular differentiation and proliferation [35]. Clonally expanded *MYB* integrations co-occur in half of the birds with *TERT* tumors. Additionally, we also observed up regulated *MYB* expression in *TERT* tumors without any ALV integrations near or within *MYB* [15]. This suggests a possible cooperation between MYB and TERT. Genes proximal to other clonally expanded integrations, which occur in the *TERT* or *MYB* tumors, might also cooperate with them for inducing oncogenic transformation. Of particular interest, integrations in *CTDSPL* and *CTDSPL2* are frequently clonally expanded, along with *TERT* and *MYB* [36]. *miR-155* integrations were also frequently seen with *MYB* in tumors (S4 Table). These putative cooperating gene players are involved in varying biological processes such as differentiation, proliferation, apoptosis, phosphorylation, immune response signaling, immortalization and DNA damage repair (S4 Table).

In order to determine common pathways activated in multiple individual tumors, we also analyzed genes near the clonally expanded integrations within individual tumors. A number of common transcription factor target gene networks were identified as common integration sites in various tumors. Among the common targets of ALV integration, the most enriched are the genes targeted by the E2F, EGR, WT1 and SP families of transcription factors (S5 Table). E2F is a well-characterized protein family that mediates both cell proliferation and apoptosis [37]. EGR (early growth response) is a family of nuclear proteins that function as transcriptional regulators and target genes required for regulating differentiation and mitogenesis [38]. The SP (specificity protein) and WT (Wilms tumor) family of transcription factors are involved in many cellular processes, including cell differentiation, cell growth, apoptosis, immune responses, and response to DNA damage [39–41]. This suggests that the ALV integrations in these genes are at the intersection of events of tumorigenic transformation. These gene players might cooperate to trigger oncogenic characteristics, thus resulting in tumor formation.

## Discussion

We report the analysis of more than 87,000 UISs, leading from early infections (in tissue culture) through to early and late tumor development. With only slight preferences for some genome features, we show that ALV exhibits a relatively random integration pattern. Due to this relatively minimal discrimination, ALV serves as a good insertional mutagenesis tool to study tumorigenesis. We utilize the OCI to empirically define the magnitude of clonality in different stages of tumorigenesis. Consistent with the clonal expansion hypothesis [25], we observe that ALV clonality increases with progressing stages of tumorigenesis We also identify putative cooperating gene players (especially *TERT* and *MYB*) and the underlying biological processes of cell differentiation, phosphorylation, immune response signaling, proliferation and regulation of apoptosis involved in tumor progression.

We observed a semi-random integration pattern for ALV. In contrast, MLV and FV exhibit a strong preference for integrations within TSS or CpG islands [42–44]. HIV-1, on the other hand, has a strong preference for integrating into transcriptional units with higher expression levels [5,6]. ALV shows a more random distribution of integration sites, similar to HTLV and MMTV [45,46]. Similar trends in the integration sites of different ALV subgroups were observed via an independent analysis in CEF, DT-40 and HeLa cells, suggesting that the ALV integration preference is not cell type specific.

We report a significant enrichment of ALV integrations within gene bodies as nearly 40% of integrations are found in genes relative to 27% at random integration sites. Barr et al. (2005) reported a similar bias for ALV integrations into transcriptional units relative to matched random sites [5]. They also reported that ALV favors integrations in transcriptional units with higher expression levels [5]; however, our data does not support this. This difference could be explained by our use of different methods to measure gene expression. Since we use RNA-seq data, in lieu of microarrays used previously (with 249 probe sets), our gene expression values might be different for the host genome. Moreover, we analyzed 15,416 proviral integration sites in this study compared to their analysis of 658 integrations, which should offer a more comprehensive analysis.

ALV displays a slight preference for integration near TSSs in tissue culture infections. We observe a further enrichment of integrations near the TSSs in tumors, as a consequence of tumorigenic selection. ALV integrations are also enriched within CpG islands as well as near spliced and expressed genes. There is a significant selection of ALV integrations away from CpG islands in the highly clonally expanded tumor cells (10 or more breakpoints). Since DNA methylation is often observed in CpG islands, ALV integrations near CpG islands may be more susceptible to repression by methylation [47]. Thus, cancer cells are likely enriched for integrations away from CpG islands, where the ALV proviruses are more likely to remain transcriptionally active.

In an emerging picture of B-cell malignancy, understanding tumor progression is an important piece of the puzzle. Here, we show that clonal expansion of ALV-infected B-cells is a key feature of malignant transformation in tumors. Approximately 100 to 500 UISs have been observed for HIV in in peripheral blood lymphocytes [48,49]. On the other hand, nearly 500–5000 UISs have been observed for a typical HTLV-1 host [21]. We observe approximately 700 to 3000 UISs in individual tumors induced by ALV mutagenesis. The most clonally expanded viral integrations appear to be early events in tumorigenesis and are expanded during progression of tumors. Therefore, this pattern of selection and expansion defines the clonal evolution of this cancer.

The distribution of the clone abundances can be quantified by an OCI value. Late stage B-cell neoplasms are associated with higher OCI values than earlier stages, and the PVL is also observed to correlate with the progressing stages of tumorigenesis. Interestingly, while the *gag* and *env* ratios appear very similar, LTR ratios are elevated for some individual tumor samples. This suggests that over the course of tumorigenesis, there are likely more deletions and rearrangements acquired in the *gag* and *env* regions of the viral genome. Further work will be necessary to identify the epigenetic factors that may influence proviral expression and tumorigenesis.

We observed a correlation between the PVL and different stages of tumor progression. As determined by the PVL analysis, a single cell in a tumor has multiple (2.3 to 4) copies of integrated ALV proviruses, suggesting multiple UISs contribute to oncogenic transformation. Therefore, loss of super-infection resistance could be involved in tumorigenesis. Alternatively, deletions in the *env* region of proviruses or mutations affecting *env* expression, identified in some tumors in previous work in our lab [18], might allow cells to overcome super-infection resistance.

Analysis of the ontology of genes flanking integration sites demonstrated a functional overrepresentation of certain pathways that are deregulated in many lymphomas [17]. Consistent with present concepts of oncogenesis and lymphomagenesis, GO analysis revealed that five major gene functions contribute to clonal dominance: regulation of proliferation, differentiation, immune response, apoptosis, and phosphorylation. Of these, cell differentiation and phosphorylation appear to be significantly altered in earlier stages of tumor progression. Interestingly, we also observed possible cooperativity between *TERT* and *MYB*, which might function together to induce oncogenic transformation. Further analysis via single cell sequencing would be useful to investigate this cooperativity.

Our work depicts a comprehensive investigation into the role of ALV integrations in lymphomas in chickens. The value of our work can be extended to mammalian systems. B-cell development in chicken and mammals is a very similar process [50]. These similarities are evident at levels of molecular changes and gene regulatory networks [51–53]. Although mice serve as a good mammalian model, in terms of oncogenesis they differ in some fundamental ways from humans. Unlike humans, the mouse telomerase enzyme is active in normal somatic cells [54]. This difference between humans and mice is important because telomerase activation is a critical step in the human oncogenic process, with telomerase activation seen in approximately 90% of human cancers [55,56]. Similar to human expression, chicken telomerase expression is down regulated in most somatic tissues [57]. Furthermore, chicken telomeres shorten with age, and telomerase activity is important for oncogenesis [58]. Therefore, chicken serves as an advantageous model over mouse, to study oncogenic events.

Limited information is available about the molecular mechanisms of lymphomagenesis, and the role of selective clonal expansion. Cells containing certain integration sites can undergo selective expansion in tumors, resulting in abundant clonal populations. We observed that in the course of tumor progression, the more transformed neoplasms contained integrations with a high number of breakpoints [25]. Via our genomic analysis of ALV integrations across progression of B-cell lymphomas, we are able to provide insights into the biological processes associated with initiation, progression, and metastasis of tumors.

## Materials and methods

### Tissue culture infections

Chicken embryo fibroblasts (CEFs) were cultured in medium 199 (Thermo Fisher Scientific) supplemented with 2% tryptose phosphate, 1% fetal calf serum, 1% chicken serum, and 1% antibiotic at 39°C and 5% CO_2_. Viruses were generated by transfecting CEFs via electroporation, with vectors RCASBP(A) and RCASBP(C) to generate viral titers of subgroups A and C respectively [59]. ALV-J virus [60] was generated from homogenates of tumors with ALV-J integrations, by passing it through a 0.22 micrometer pore size filter. The collected supernatant from tumors was in turn used to infect CEFs. CEFs were grown at approximately 40% confluency and were infected with ALV subgroup A, C or J at an MOI of 1–2. The cells were collected at 48 hours and 120 hours post infection for DNA isolation. DT-40 cells were cultured in Dulbecco’s modified eagle medium (Thermo Fisher Scientific), 10% fetal calf serum, 5% chicken serum, 5% tryptose phosphate, and 1% antibiotic at 37°C and 5% CO2. DT-40s were grown at approximately 40% confluency and were infected with ALV subgroup C at an MOI of 1–2. The cells were collected at 48 hours post infection for DNA isolation. HeLa cells (ATCC) were cultured in Dulbecco’s modified eagle medium, 10% fetal bovine serum (FBS), and 1% antibiotic at 37°C and 5% CO_2._ To generate ALV pseudo-typed with vesicular stomatitis virus glycoprotein (VSV-G), CEFs were co-transfected via electroporation, with pMD.G (VSV-G envelope plasmid) and RCASBP(C) plasmid [61]. Viral supernatant was collected after 48 h, filtered through a 0.22-micrometer filter, and concentrated by polyethylene glycol (PEG) precipitation (10% PEG8000) [62]. This concentrate of pseudo-typed ALV was used to infect HeLa cells and cells were collected 48 hours post infection for DNA isolation.

### Tumor induction and tissue isolation

5 or 10-day-old chicken embryos were injected with ALV-LR9, ALV- ΔLR9, ALV-G919A, or ALV-U916A as described previously [10,17]. Chickens were observed daily and were euthanized when apparently ill or at 10–12 weeks after hatching. A total of 72 tissues were selected for characterization by high-throughput sequencing (S2 Table). Three uninfected tissues and several non-tumor tissues from infected birds were sequenced to serve as controls. All of the B-cell lymphomas included in the study were rapid-onset lymphomas, arising within 10–12 weeks. LR-9 is an ALV subgroup A recombinant virus consisting of *gag*, *pol*, and *env* genes derived from UR2-associated virus and LTRs derived from ring-necked pheasant virus [63]. ALV-ΔLR-9 contains a deletion in the *gag* gene, causing increased splicing to downstream genes [11]. ALV-G919A contains a silent mutation in the NRS [10]. Tumors were collected from primary bursal (B) tissue or metastasized liver (L), kidney (K) or spleen (S) tissues.

### Ethics statement

Five- and ten-day old chicken embryos were injected with virus. Chickens injected include inbred SC White Leghorn line embryos (Hy-Line International, Dallas Center, IA), and SPAFAS embryos (Charles River). Chickens were euthanized at 10–12 weeks post hatching. Institutional Animal Care and Use Committee (IACUC) approval was obtained at the University of Delaware and the Fred Hutchinson Cancer Research Center.

### Integration site mapping and quantification

DNA from ALV infected cultured cells or tumor samples were isolated. The sequencing libraries were prepared as described previously [16]. Five micrograms of purified genomic DNA was sonicated with a Bioruptor UCD-200. End repair, A-tailing, and adapter ligation were performed as described previously [21] (adapter short arm, P-GATCGGAAGAGCAAAAAAAAAAAAAAAA, and adapter long arm, CAAGCAGAAGACGGCATACGAGATXXXXXXGTGACTGGAGTTCAGACGTGTGCTCTTCCGATC*T, where “X’s” denote the barcode sequence, “P” denotes phosphorylation, and “*” denotes a phosphorothioate bond). Nested PCR was performed to enrich the library for proviral junctions. The first PCR step had 23 cycles and employed an ALV-specific primer (CGCGAGGAGCGTAAGAAATTTCAGG) between the 3’ LTR and *env* and a primer (CAAGCAGAAGACGGCATACGAGAT) within the adapter that was attached by ligation in the previous step. In the second round of PCR, a primer (AATGATACGGCGACCACCGAGATCTACACTCGACGACTACGAGCACATGCATGAAG) near the 3’ end of the LTR was used. This primer ended 12 nucleotides short of the junction between viral and genomic DNA. This primer was paired with an adapter-specific primer on the opposite side of the fragment, which overlapped the adaptor’s bar code sequence (CAAGCAGAAGACGGCATACGAGATXXXXXX). Libraries were quantified by quantitative PCR (qPCR) and then under- went single-end 100-bp multiplexed sequencing on the Illumina Hi-Seq 2000. A custom sequencing primer (ACGACTACGAGCACATGCATGAAGCAGAAGG) was used, which hybridized near the end of the viral 3’ LTR, 5 nucleotides short of the proviral/genomic DNA junction. The resulting reads could be validated as genuine integrations by verifying that they began with the last 5 nucleotides of the proviral DNA, CTTCA. The last two nucleotides of the unintegrated proviral DNA, TT, are cleaved by ALV integrase upon integration, so the lack of these 2 nucleotides in the read acted as further validation of a true viral integration.

### Sequence analysis

Reads were first curated with a custom python script to remove sequences that did not begin with the last five nucleotides of viral DNA, “CTTCA” [16,17]. The files were then uploaded to Galaxy [64–66], which was used to perform downstream analyses. In Galaxy, first the quality scores were converted to Sanger format with FastQ Groomer v1.0.4 [67]. CTTCA and adapter sequences were then trimmed using the Galaxy Clip tool v1.0.1. This tool also removed reads containing an N and reads less than 20 nucleotides in length after adapter removal. The remaining reads were mapped with bowtie [66] using the *Gallus gallus* 4.0 genome (Nov. 2011). Sequences were aligned using a seed length of 28 nucleotides, with a maximum of 2 mismatches permitted in the seed. All alignments for a read were suppressed if more than one reportable alignment existed. This was done to prevent multiple mapping and ensure that reads correspond to only unique integration sites. 100,000 random mapped reads were selected from each sample to be used for further analysis. If less than 100,000 reads were present for a sample, all available reads were used. A custom Perl pipeline developed in the lab was used to analyze the aligned reads output from bowtie [16,17]. This custom pipeline identified unique integration locations, and calculated the number of reads and sonication breakpoints for each integration site. It also identified hotspots of integration and common integration sites among multiple samples. Integrations from two unrelated barcodes on the same sequencing lanes were omitted via our pipeline. The pipeline source code is available upon request. The integration sites identified in our work are deposited at the NCI Retrovirus Integration Database (RID) (https://rid.ncifcrf.gov/) [68].

### Analysis of ALV integrations with respect to different genome annotations and features

Reads for the junctions of proviral integration and genomic DNA were mapped with Bowtie [69]. Only reads that mapped uniquely to the genome were utilized for further analysis. This step filtered out reads that originate from repetitive elements. Mapped reads from all samples were then combined into a single file and analyzed with HOMER [23]. HOMER calculates the enriched features at each integration locus as well the proximity to closest transcription start site. A random integration control data set was generated with Bedtools Random [70]. The genomic DNA sequences corresponding to the genomic coordinates obtained from Bedtools Random were extracted from the Gallus gallus 4 genome using the Galaxy tool Extract Genomic DNA. Control sequences were mapped with Bowtie and analyzed with HOMER using the same parameters as for ALV integrations. Proximity to CpG islands was determined using the WindowBed tool in Galaxy [66].

We note that our calculations are subject to certain biases. This includes, but is not limited to, an underestimate of the chicken or human genome sizes due to unsequenced gaps or overlapping sequences. Furthermore, an aberrant karyotype, which might exist in the transformed HeLa [71] or the DT-40 cells [72], was not taken into account for our analysis. However, as previously determined by Narezkina et al. (2004), despite the aberrant karyotype in HeLa cells, the ratio between the genome size and the gene number in HeLa cells is equivalent to that of the normal human genome [4].

### Analysis of ALV integrations with respect to gene expression and levels of splicing

The ensembl *Gallus gallus* 4 genome was utilized to obtain reference information for the transcript count and number of introns for all transcriptional units in the chicken genome. If an integration occurs within a gene, then the corresponding gene is used for all subsequent analysis. If an integration occurs in an intergenic region, then the nearest gene is used for all subsequent analysis. RNA-seq data for analysis of CEFs was downloaded from the public Sequence Read Archive (SRA) database (SRA accession no. SRP107761) [73]. A custom Python script was utilized to associate the expression, transcript count and number of introns of a gene with the number of ALV integrations proximal to or within the given gene. A matched random control set, generated as mentioned above, was used as a control. The Python source code is available upon request.

### Proviral load quantification

PVL was measured by quantitative polymerase chain reaction (qPCR) of ALV-LR9 for *env* (primers CCTGAAACCCAGTGCATAAGG and CTAGCTGTGCAGTTCACCGT), *gag* (primers GTTTAGAGAGGTTGCCCGAC and GTCAATGATCACCGGAGCCC) and LTR (CGAACCACTGAATTCCGCAT and GAATCAACGGTCCGGCCATC); and *HMG14b* (primers ACTGAAGAGACAAACCAAGAGC and CCAGCTGTTTTAGACCAAAGAATAC) using Q SYBR green Supermix (Bio-Rad) according to the manufacturer’s protocol on a Bio-Rad C1000 thermal cycler/CFX96 Real-Time System. We assumed a single copy of *env* and *gag* and 2 copies each of *HMG14b* and the LTR per cell. *HMG14b* is a known single copy gene in the chicken genome and thus, was used as a housekeeping reference gene [74]. Thermal cycling conditions were 95°C for 20 seconds and 40 cycles each of 95°C for 1 second followed by 53°C for 30 seconds. Quantitative PCR (qPCR) was performed in duplicate, with each sample present in technical duplicate during each run. The results were normalized to those for normal bursa using the comparative threshold cycle (*CT*) method.

### Statistical analysis for oligoclonality index calculations

Statistical analysis for clonality index was carried out using R version 2.15.2 (http://www.R-project.org/). The oligoclonality index (OCI; Gini coefficient) was calculated using the R package sonicLength (http://soniclength.r-forge.r-project.org/) as described previously [21,22].

### Gene ontology analysis

Functional profiling of genes and ontology analysis for the clonally expanded integrations was conducted with g:profiler, using an ordered query option (http://biit.cs.ut.ee/gprofiler/) [30].

## Supporting information

S1 Fig Association of ALV integrations within genes with the expression of transcriptional units. Expression levels of the chicken RefSeq transcriptional units (6,060) in CEFs were analyzed using available RNA-seq data sets, as described in materials and methods. Gene expression levels were divided, based on FPKM (Fragments Per Kilobase of transcript per Million mapped reads) expression values, into 13 bins. Numbers of integrations that only occur within genes were then plotted into the bins as a percentage of the total and compared to random events.
(PDF)

S2 Fig Distribution of ALV integrations relative to the expression of transcriptional units in tumors. Expression levels of the chicken RefSeq transcriptional units (6,060) in tumors C7L and D2L were analyzed using available RNA-seq data sets, as described in materials and methods. Gene expression levels were divided, based on FPKM (Fragments Per Kilobase of transcript per Million mapped reads) expression values, into 13 bins. Numbers of integrations that occur near or within genes were then plotted into the bins as a percentage of the total and compared to random events.
(PDF)

S3 Fig Correlation between ALV integrations and the number of alternative transcripts in HeLa cells. Each transcription unit is assigned to a group based on the number of known transcripts that originate from it. Percentage of random and ALV integration events are plotted within each group for comparison.
(PDF)

S4 Fig Pie charts depicting ALV integrations in all the neoplasms analyzed. Pie charts are categorized as primary tumors (bursas), metastases (liver, kidney and spleen), and neoplastic follicles and inflammation. Non-tumors are depicted as controls. Each pie represents an individual tumor with approximately the top 200 breakpoints observed for ALV integrations. Each slice of the pie represents a unique integration with the corresponding number of sonication breakpoints observed for that integration. In case of fewer than 200 breakpoints, all the breakpoints are depicted. For the most clonally expanded integrations, the known biological function of the gene player is described on the slices.
(PDF)

S5 Fig Comparisons of primary (bursa) and secondary (liver, spleen or kidney) tumors from the same birds. The top 200 breakpoints of clonally expanded unique integration sites are depicted within each pie chart, along with the list of most proximal host genes.
(PDF)

S6 Fig Clonally expanded integrations from different slices of a tumor suggest homogeneity of metastases. Top 10 most clonally expanded integrations from different slices of primary tumor (C2B) and liver metastases (C6L, C7L and D2L) are illustrated. Individual pie charts represent UISs and corresponding extent of clonal expansion (as breakpoints) from a different slice of the tissue. Slices were chosen randomly from three distinct portions of tumor mass, including peripheral and interior regions.
(PDF)

S7 Fig Proviral load values are calculated for different tissues, using the env, gag or LTR regions of ALV. Uninfected normal tissues of normal bursa (NB) and normal liver (NL) are depicted as controls.
(PDF)

S1 Table Number of ALV integrations analyzed in tissue culture and tumors. The different ALV subgroups (A, C and J) and cell types (CEF, DT40 and HeLa) used for analysis are denoted.
(PDF)

S2 Table List of different normal and infected tissues analyzed for ALV integrations. (PDF)

S3 Table List of gene players (described with respective ensemble IDs) involved in the corresponding biological processes in the different stages of tumor progression. (XLSX)

S4 Table Gene players that might cooperate with *TERT* and/or *MYB* in tumorigenesis. (PDF)

S5 Table Common transcription factor target gene networks are enriched ALV integration sites in many tumors. (XLSX)
