# Supplementary figures and images for: Selection for avian leukosis virus integration sites determines the clonal progression of B-cell lymphomas
Source: PLoS Pathog. 2017 Nov 3;13(11):e1006708. doi: 10.1371/journal.ppat.1006708 (PMC5687753; doi:10.1371/journal.ppat.1006708)

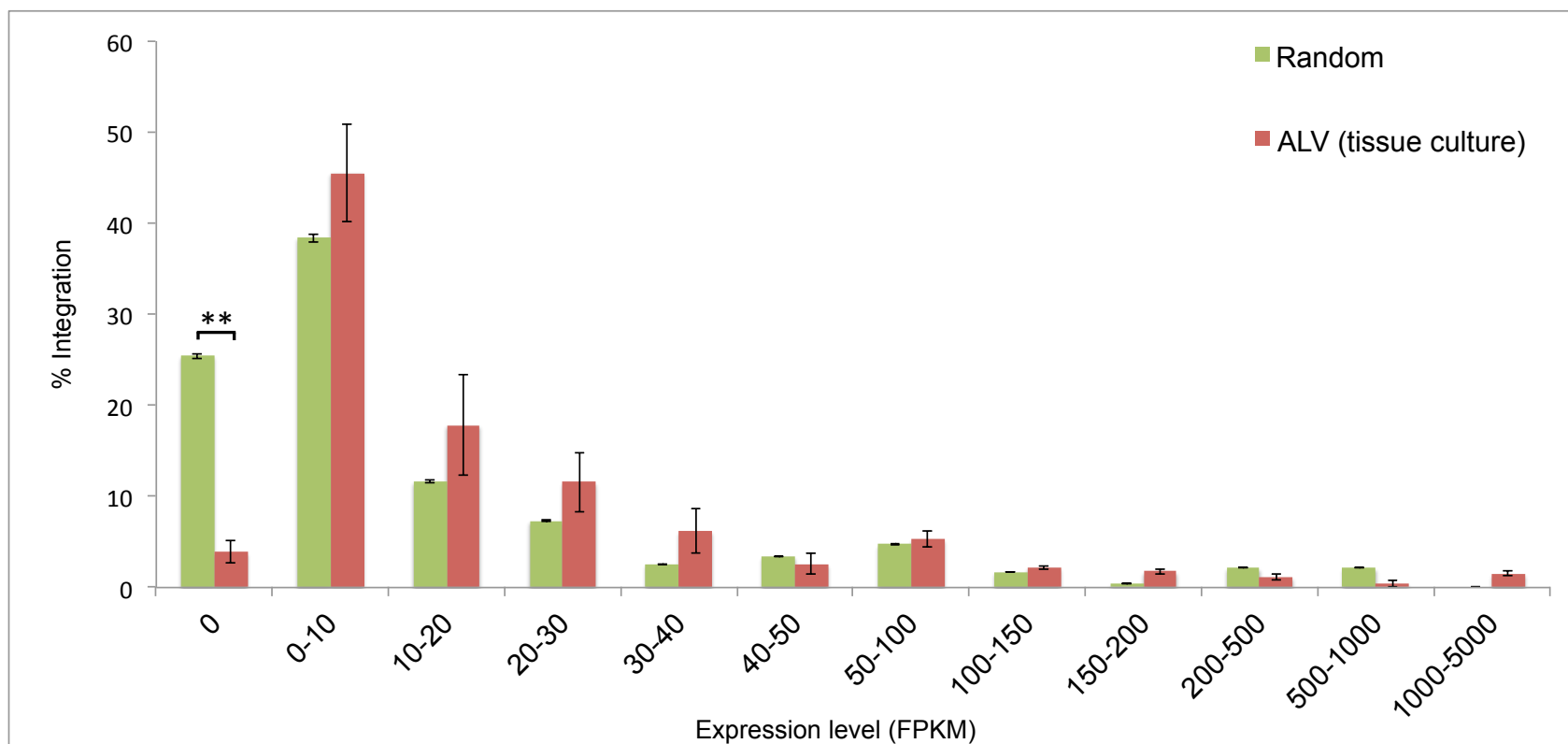

\*\* p < 0.01

Supplement: S1 Fig — Expression levels of the chicken RefSeq transcriptional units (6,060) in CEFs were analyzed using available RNA-seq data sets, as described in materials and methods. Gene expression levels were divided, based on FPKM (Fragments Per Kilobase of transcript per Million mapped reads) expression values, into 13 bins. Numbers of integrations that only occur within genes were then plotted into the bins as a percentage of the total and compared to random events. (PDF) [file ppat.1006708.s001.pdf]

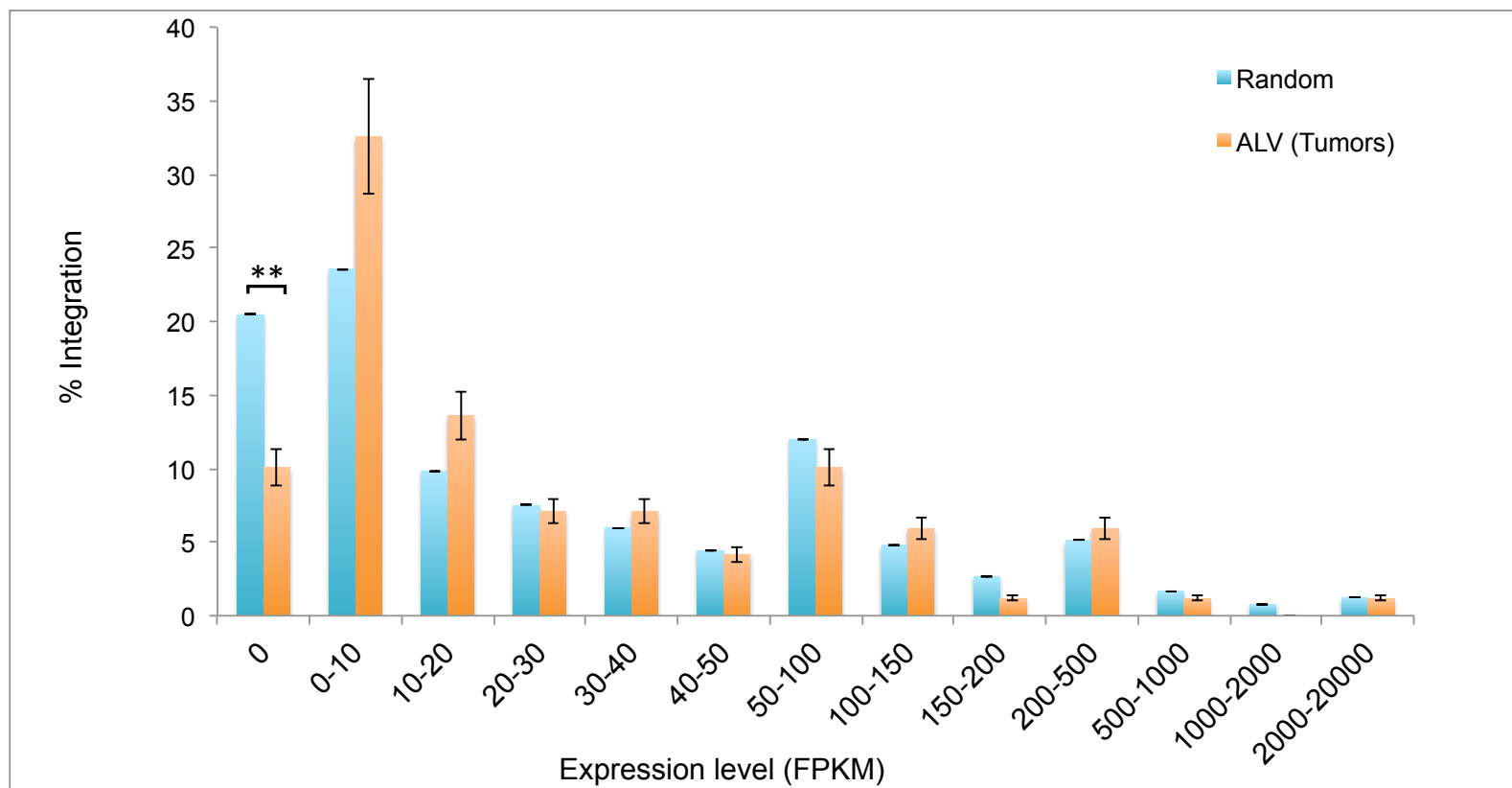

\*\* p < 0.01

Supplement: S2 Fig — Expression levels of the chicken RefSeq transcriptional units (6,060) in tumors C7L and D2L were analyzed using available RNA-seq data sets, as described in materials and methods. Gene expression levels were divided, based on FPKM (Fragments Per Kilobase of transcript per Million mapped reads) expression values, into 13 bins. Numbers of integrations that occur near or within genes were then plotted into the bins as a percentage of the total and compared to random events. (PDF) [file ppat.1006708.s002.pdf]

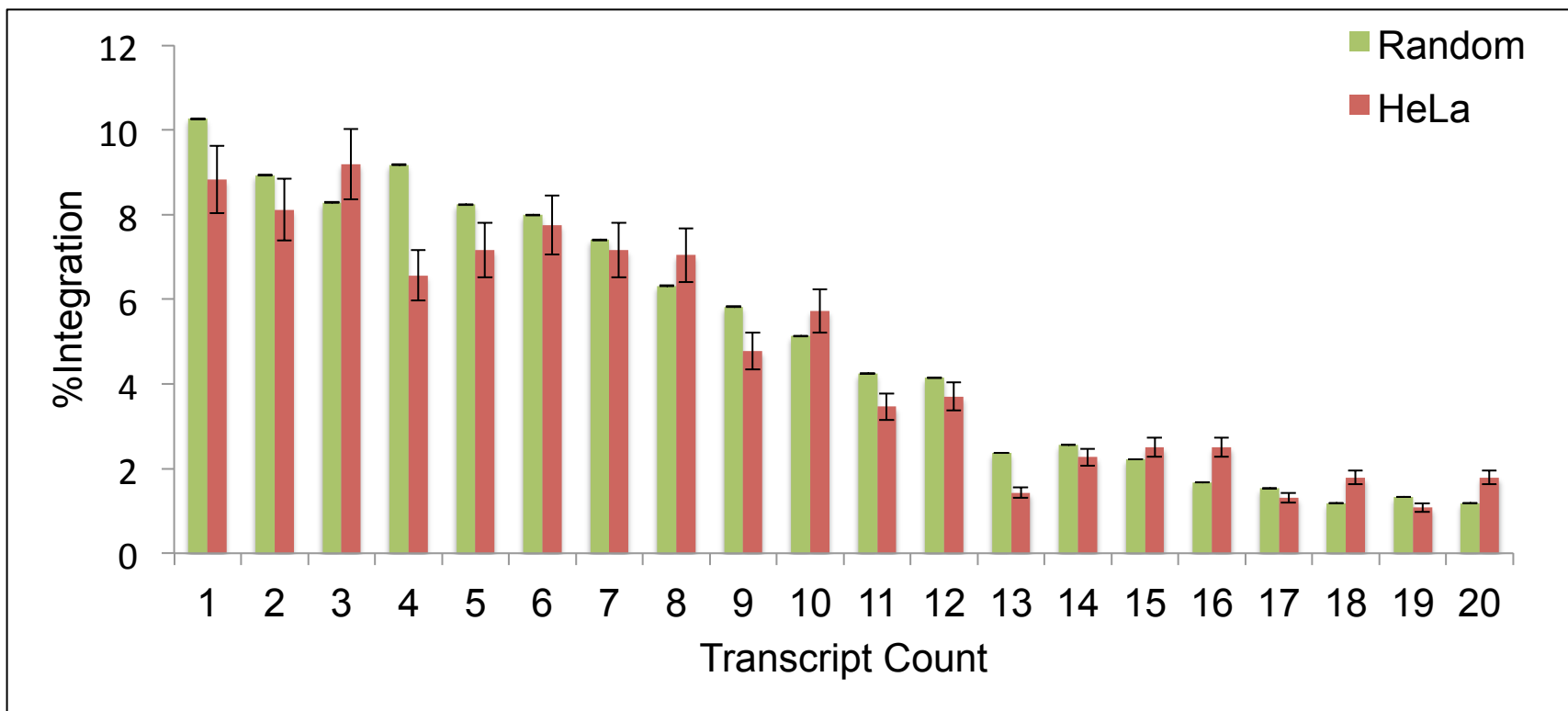

Supplement: S3 Fig — Each transcription unit is assigned to a group based on the number of known transcripts that originate from it. Percentage of random and ALV integration events are plotted within each group for comparison. (PDF) [file ppat.1006708.s003.pdf]

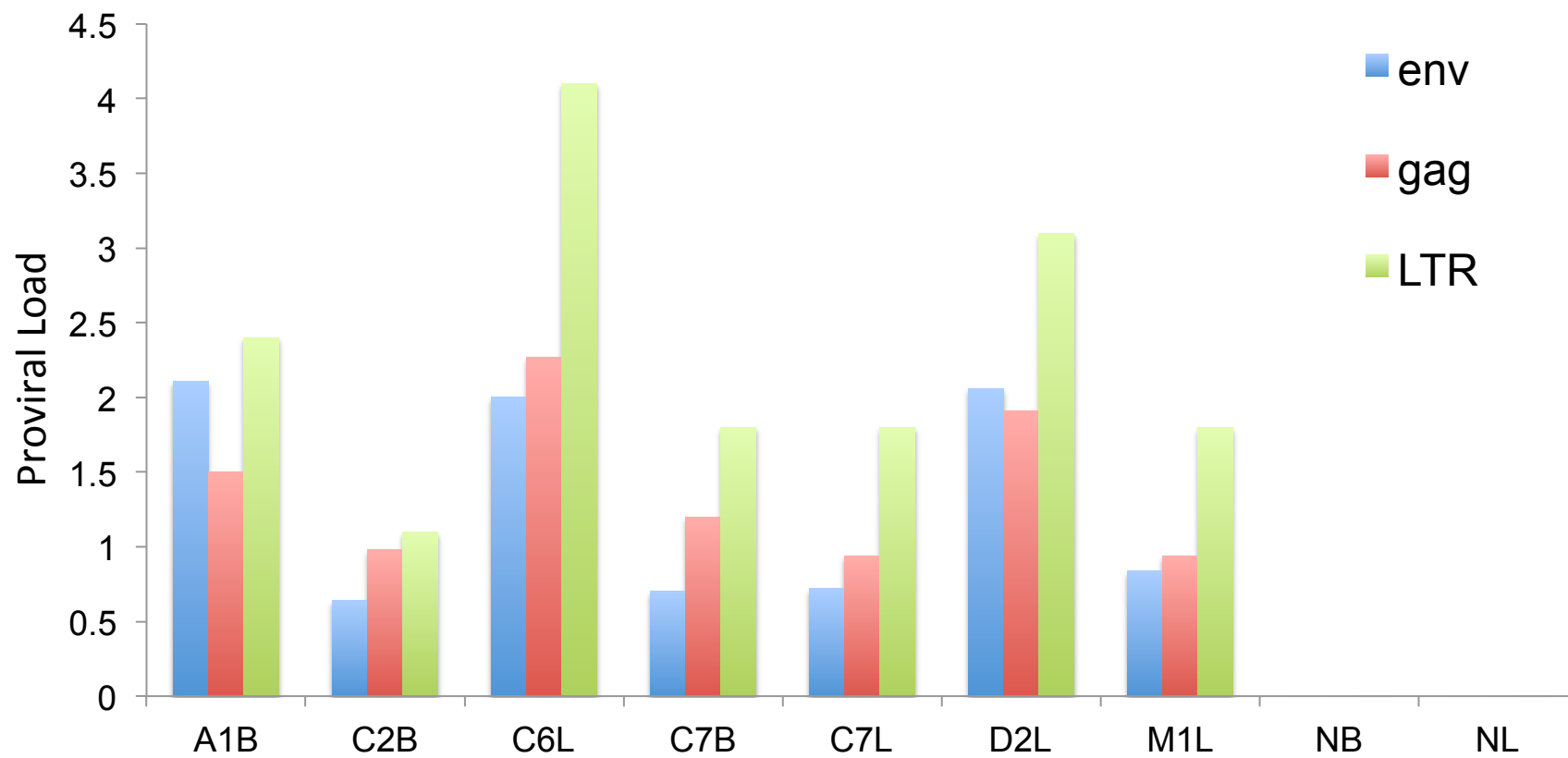

Supplement: S7 Fig — Uninfected normal tissues of normal bursa (NB) and normal liver (NL) are depicted as controls. (PDF) [file ppat.1006708.s007.pdf]
