# Supplementary material for: Selection for avian leukosis virus integration sites determines the clonal progression of B-cell lymphomas
Source: PLoS Pathog. 2017 Nov 3;13(11):e1006708. doi: 10.1371/journal.ppat.1006708 (PMC5687753; doi:10.1371/journal.ppat.1006708)
Supplement: S4 Fig — Pie charts are categorized as primary tumors (bursas), metastases (liver, kidney and spleen), and neoplastic follicles and inflammation. Non-tumors are depicted as controls. Each pie represents an individual tumor with approximately the top 200 breakpoints observed for ALV integrations. Each slice of the pie represents a unique integration with the corresponding number of sonication breakpoints observed for that integration. In case of fewer than 200 breakpoints, all the breakpoints are depicted. For the most clonally expanded integrations, the known biological function of the gene player is described on the slices. (PDF) [file ppat.1006708.s004.pdf]

Non-Tumor

C2K Non-Tumor

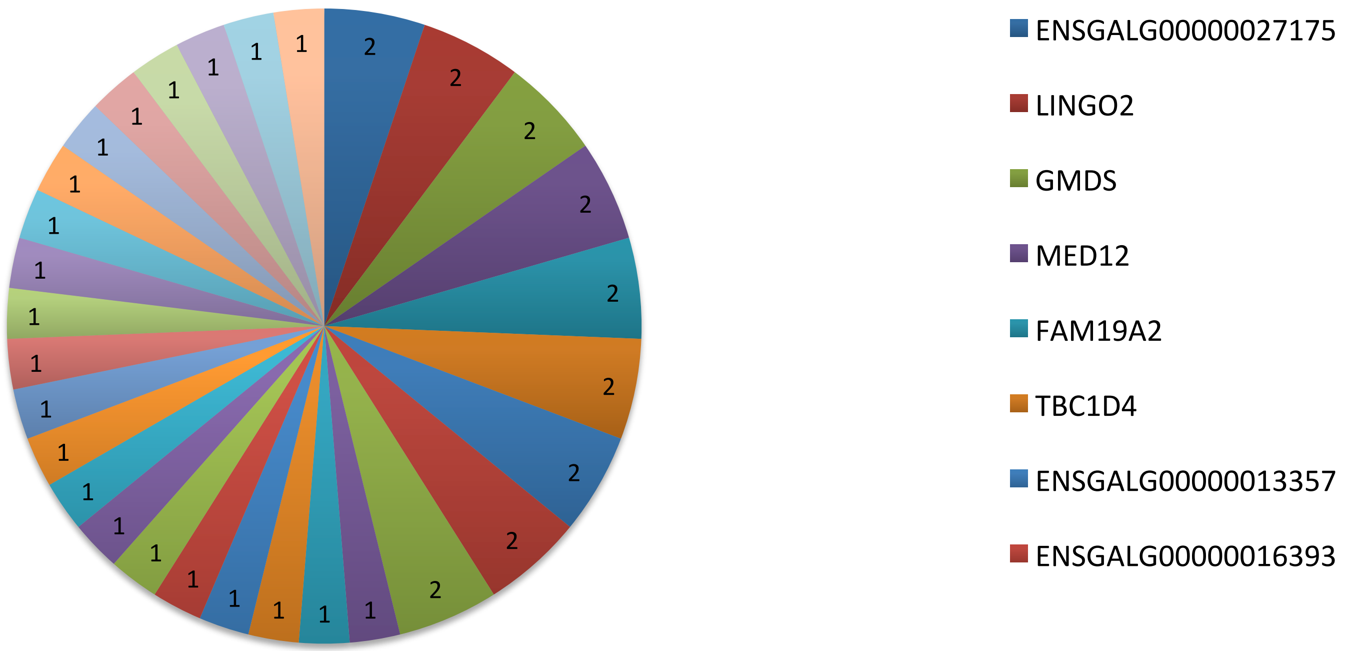

C2Br Non-Tumor

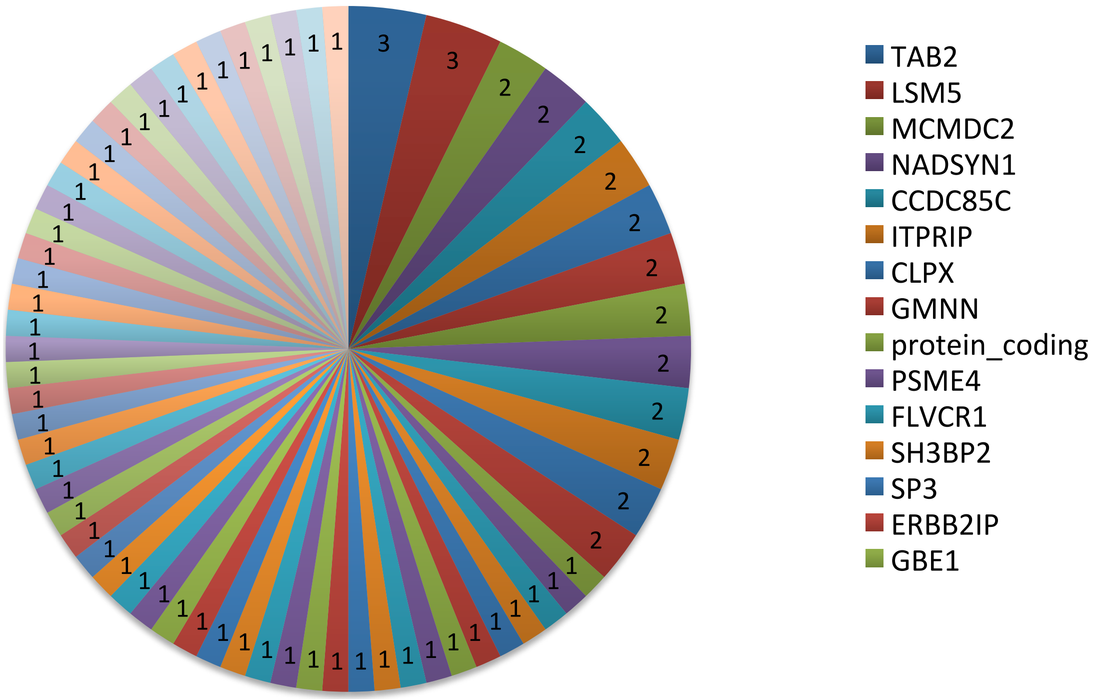

C4Br Non-Tumor

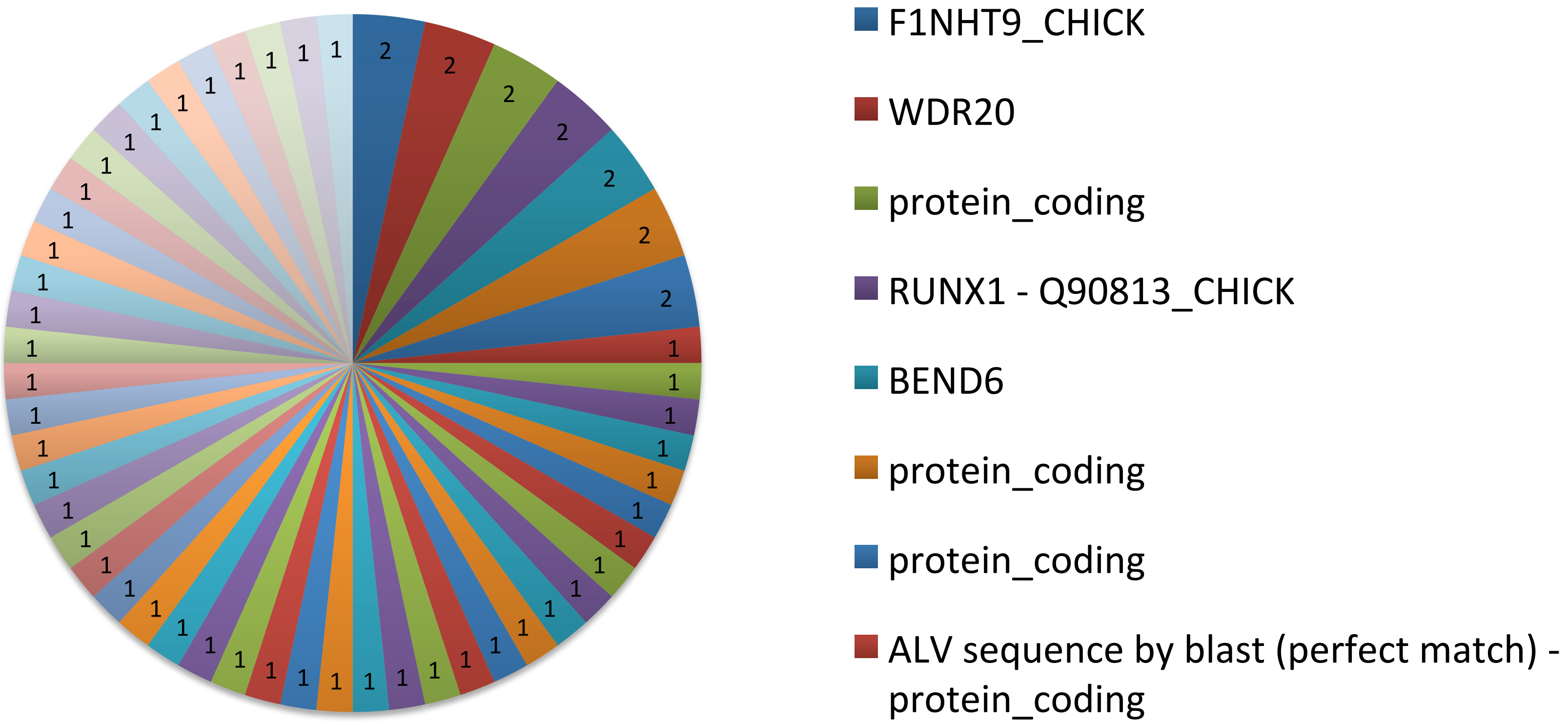

D1K Non-Tumor

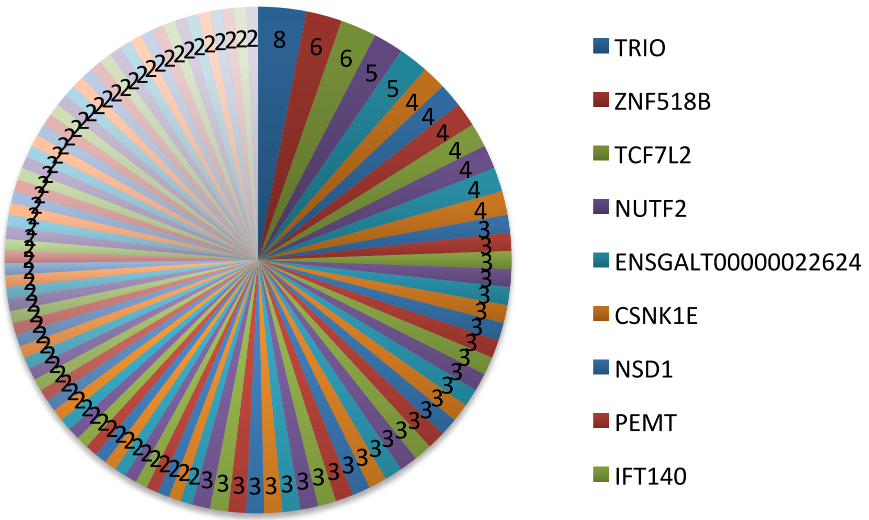

D3K Non-Tumor

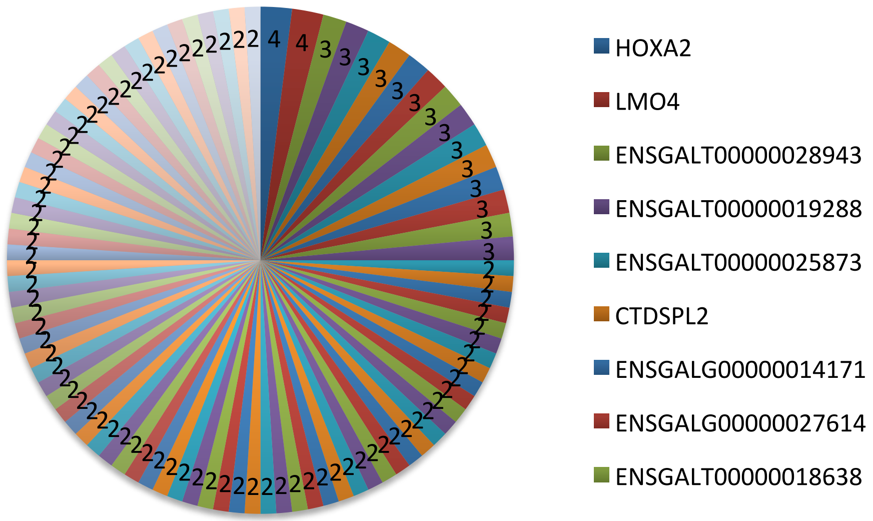

Inflammation

B8L Inflammation

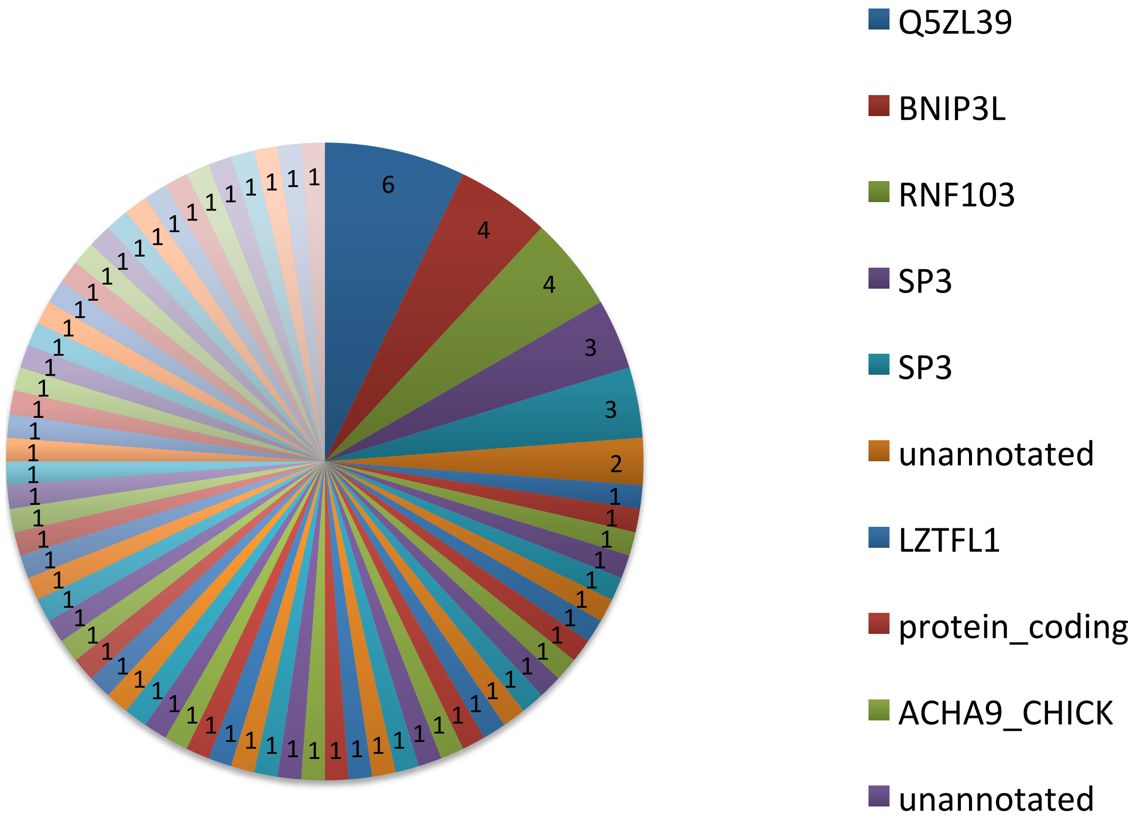

D4L Inflammation

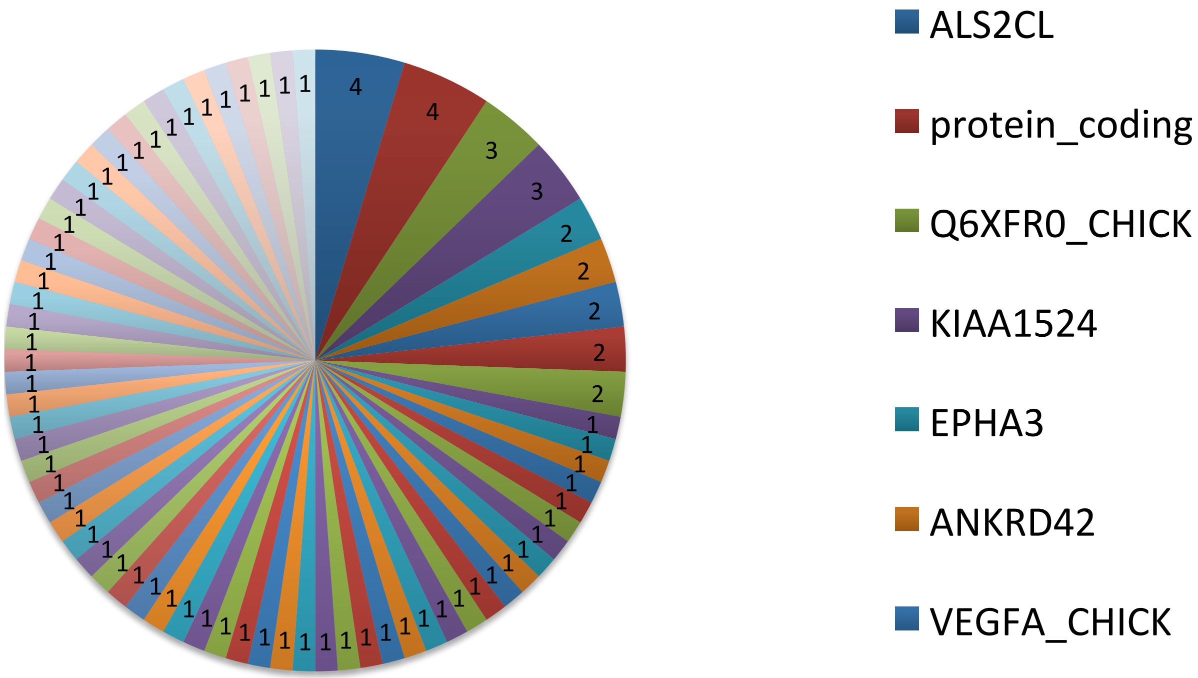

Neoplastic follicle

A6B Neoplastic Follicle

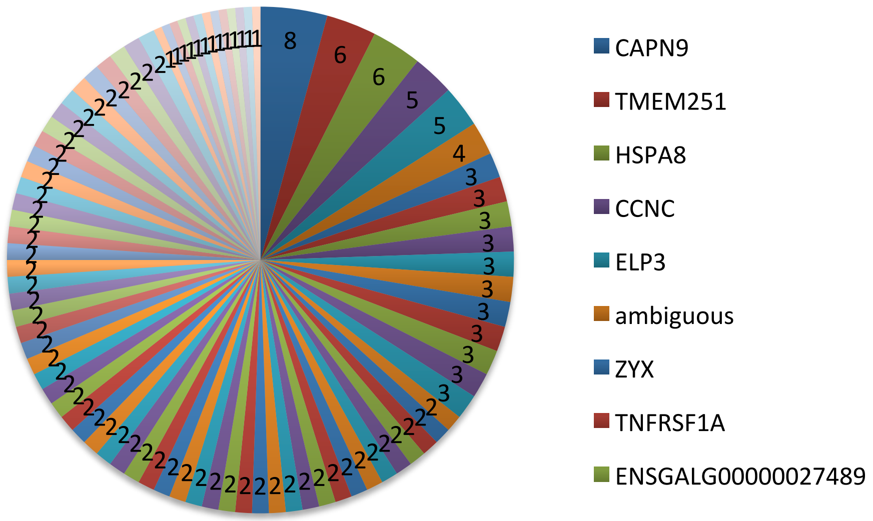

A9B Neoplastic Follicle

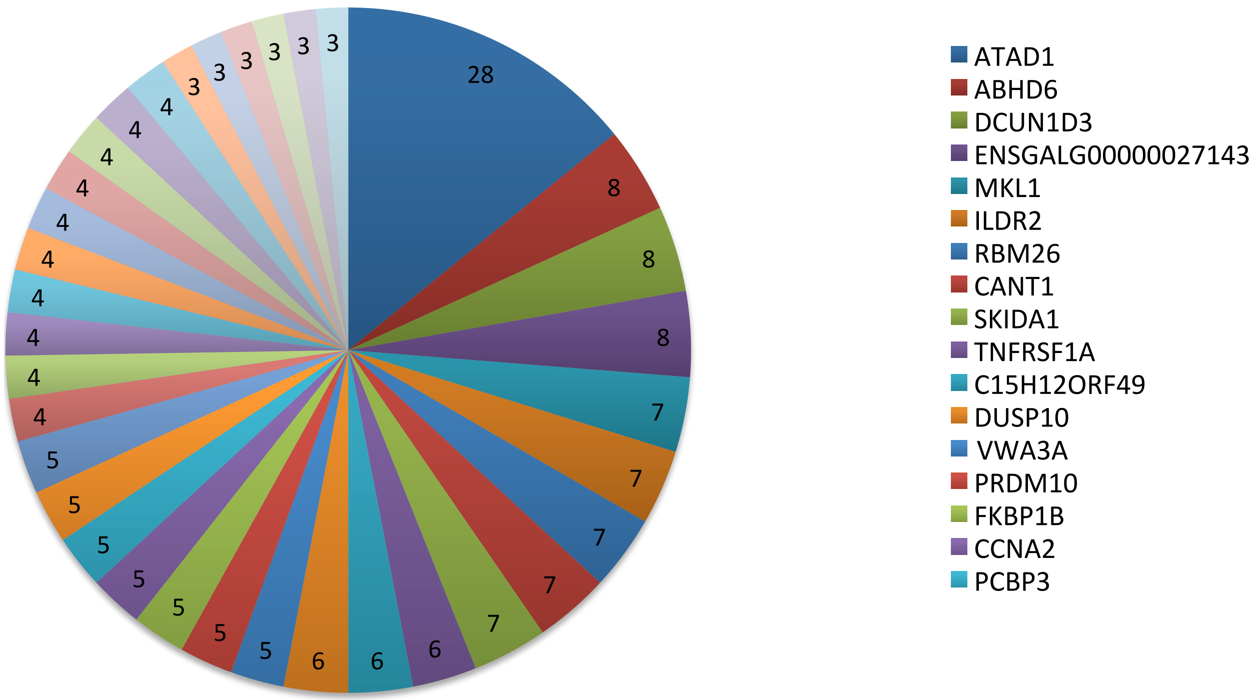

D1B Neoplastic Follicle

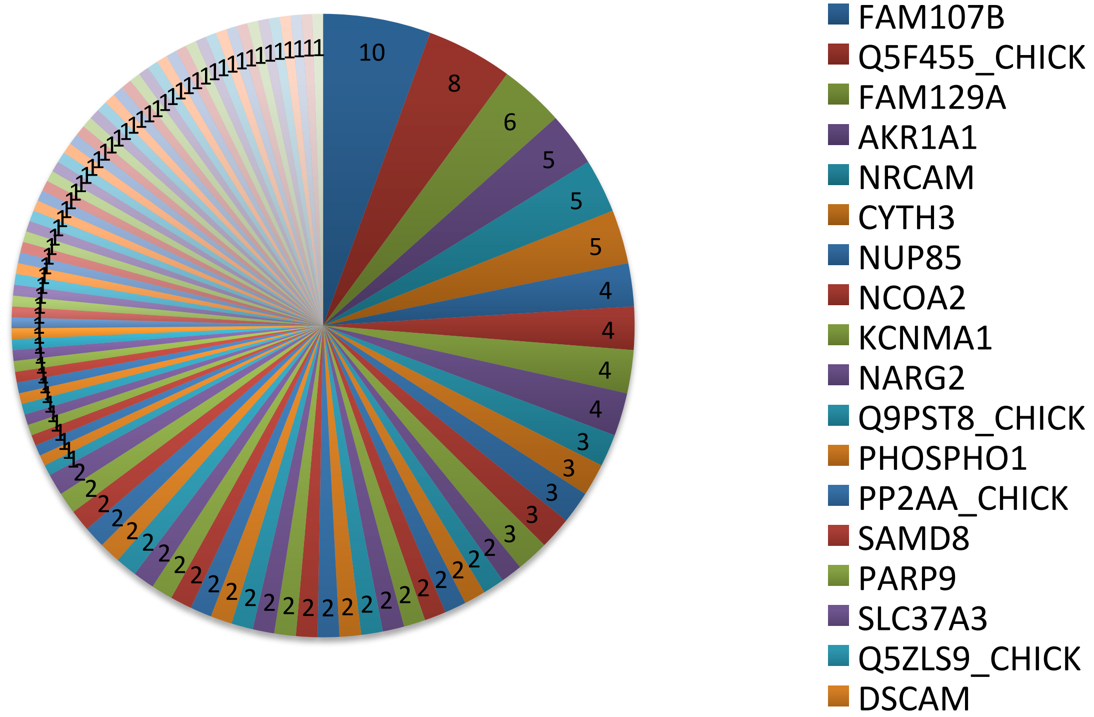

D10B Neoplastic Follicle

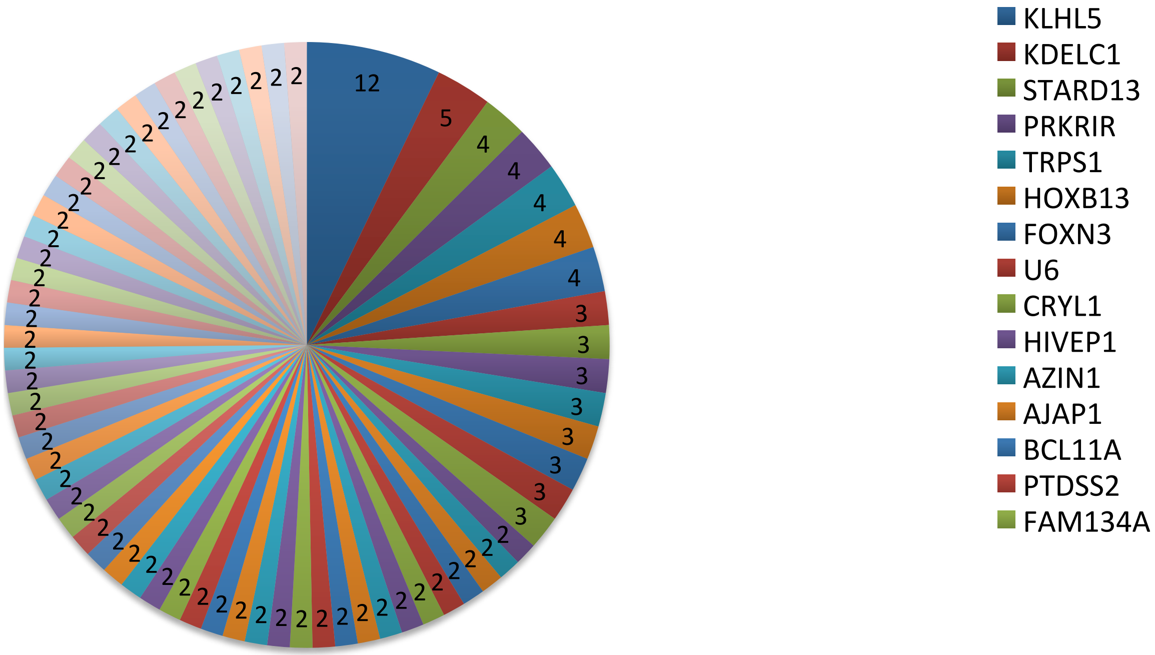

D6B Neoplastic Follicle

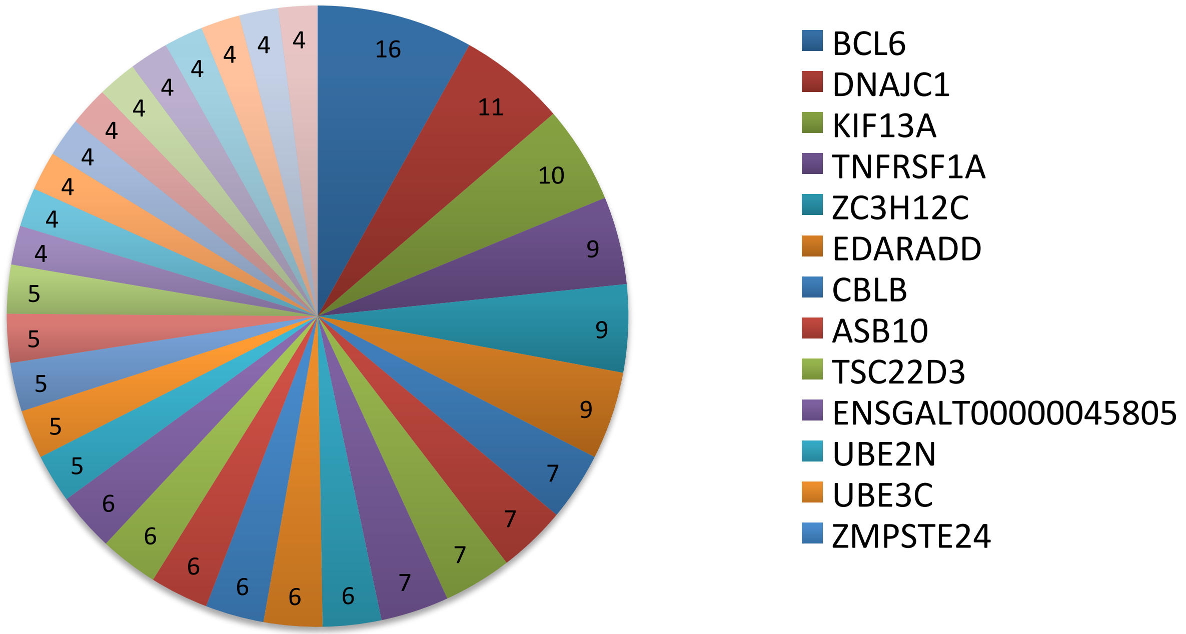

Bursa Tumors

A1B Tumor

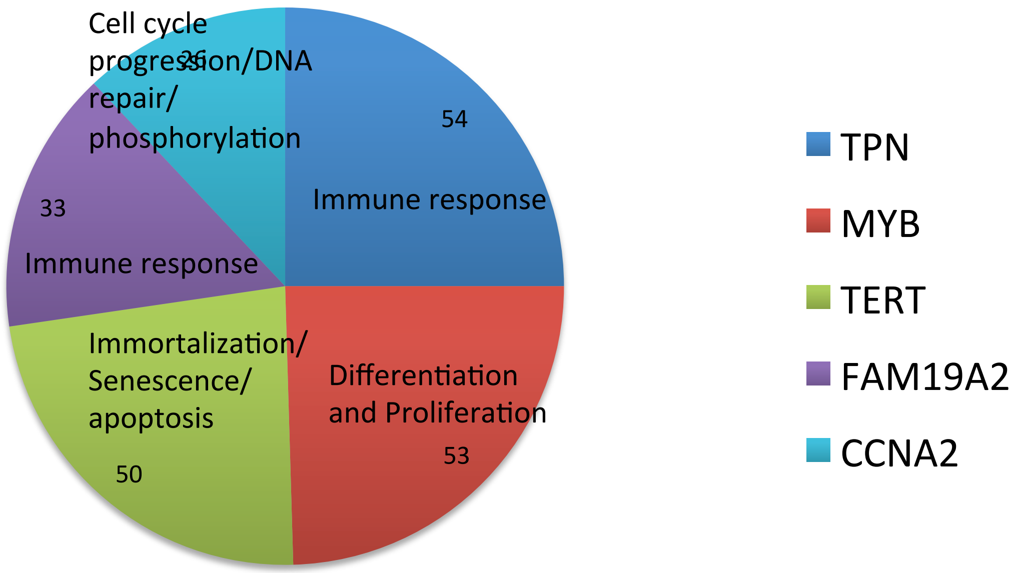

Cell cycle progression/DNA repair/ phosphorylation
26
54
Immune response
33
Immune response
Immortalization/ Senescence/ apoptosis
50
Differentiation and Proliferation
53
TPN
MYB
TERT
FAM19A2
CCNA2

A2B Tumor

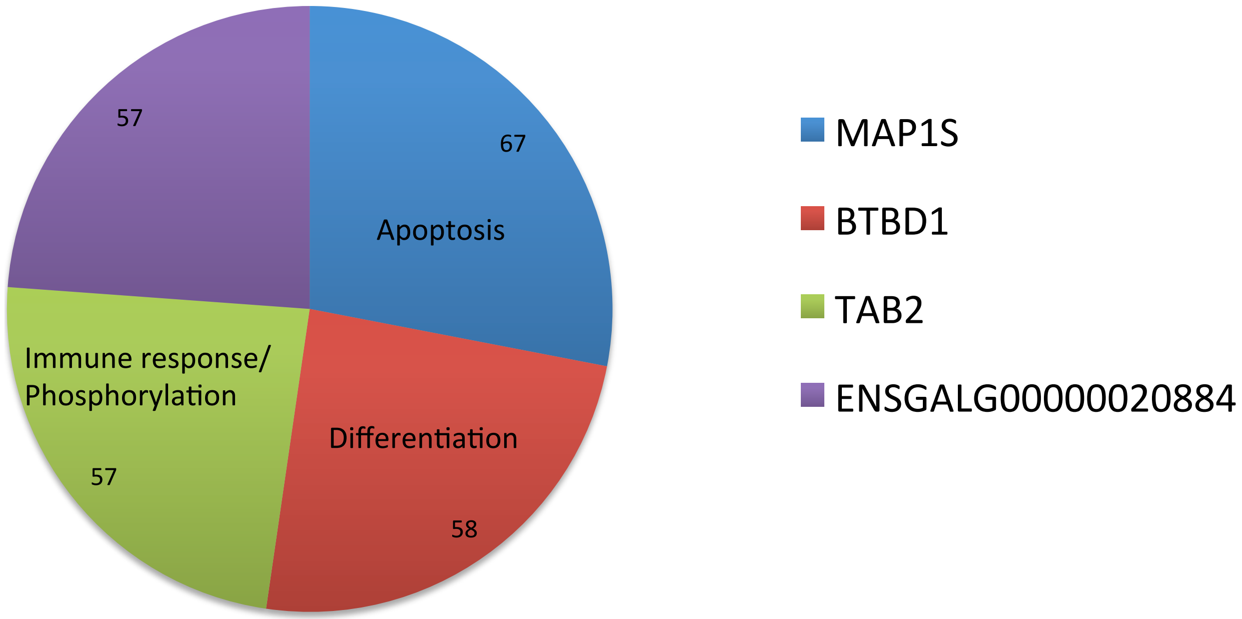
57
67
Apoptosis
Immune response/
Phosphorylation
Differentiation
57
58
MAP1S
BTBD1
TAB2
ENSGALG00000020884

A4B Tumor

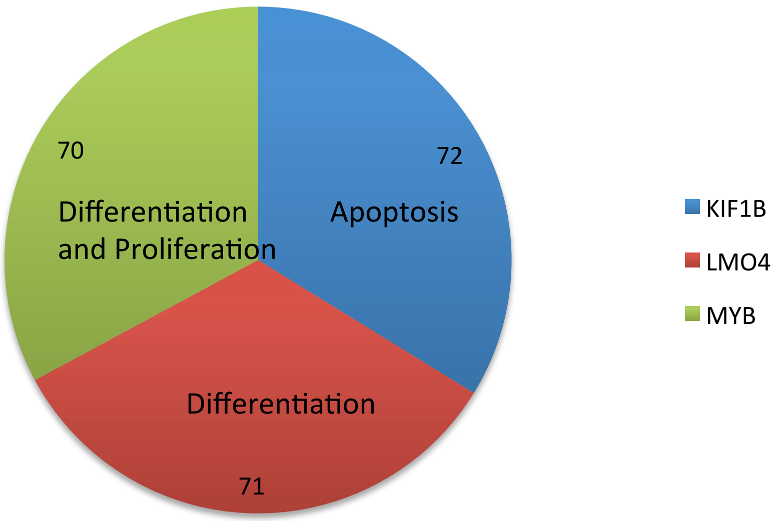
70
72
Differentiation
and Proliferation
Apoptosis
Differentiation
71
KIF1B
LMO4
MYB

A5B Tumor

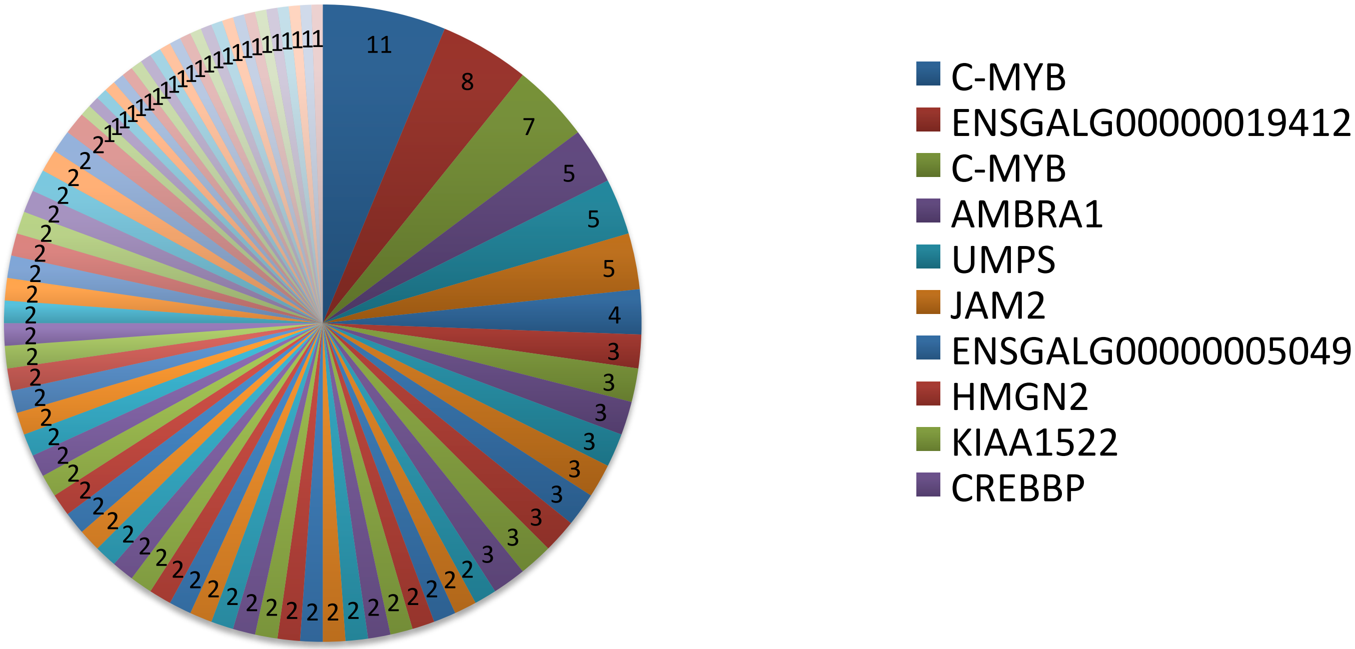
C-MYB
ENSGALG00000019412
C-MYB
AMBRA1
UMPS
JAM2
ENSGALG00000005049
HMGN2
KIAA1522
CREBBP

A8B Tumor

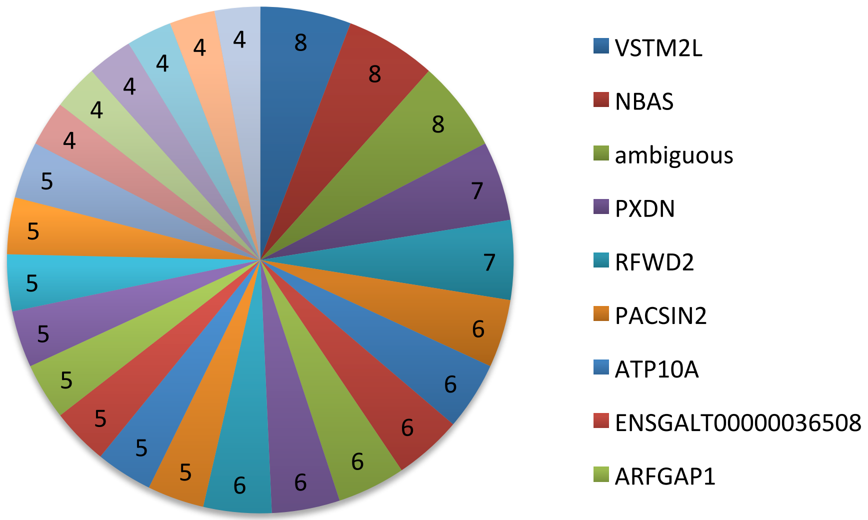
8
8
8
7
7
6
6
6
6
6
6
5
5
5
5
5
5
5
5
4
4
4
4
4
4
VSTM2L
NBAS
ambiguous
PXDN
RFWD2
PACSIN2
ATP10A
ENSGALT00000036508
ARFGAP1

B3B Tumor

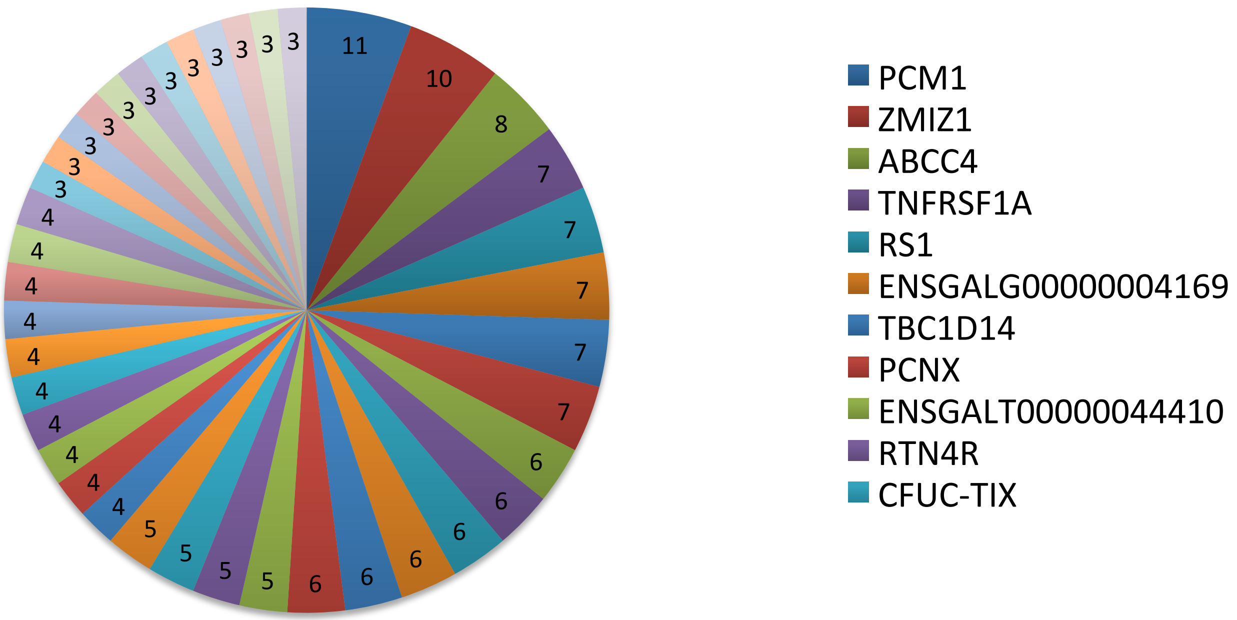

11
10
8
7
7
7
7
7
6
6
6
6
6
6
5
5
5
5
4
4
4
4
4
4
4
4
4
4
4
3
3
3
3
3
3
3
3
3
3
3
3
PCM1
ZMIZ1
ABCC4
TNFRSF1A
RS1
ENSGALG00000004169
TBC1D14
PCNX
ENSGALT00000044410
RTN4R
CFUC-TIX

B4B Tumor

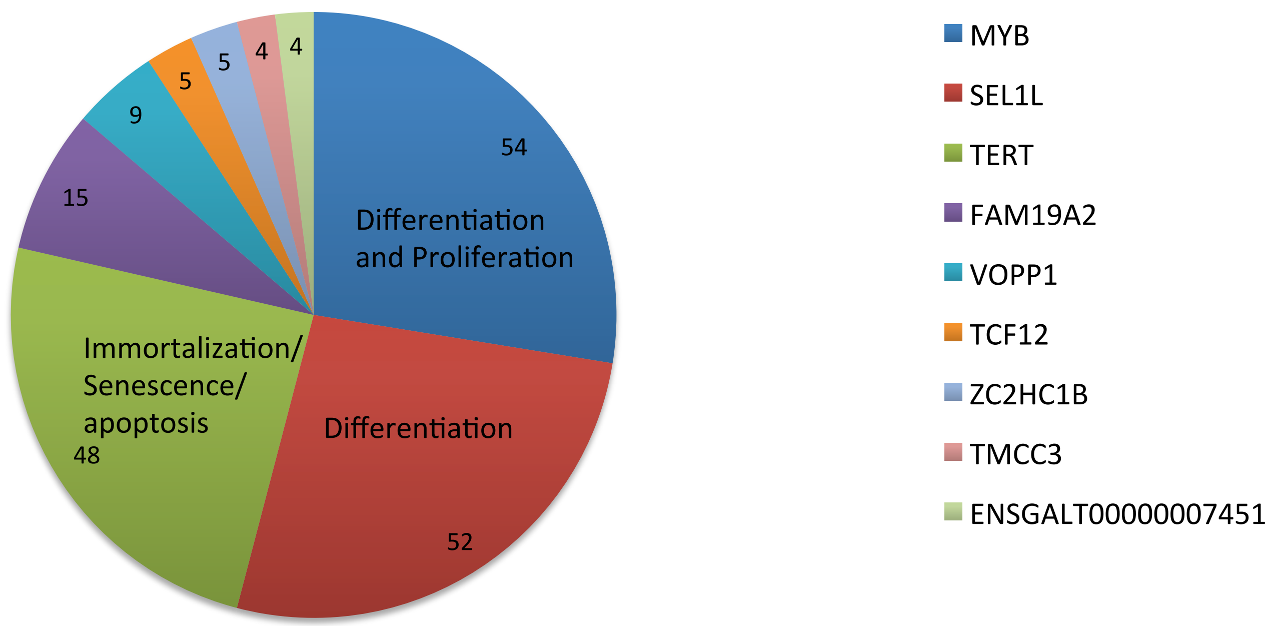

54
Differentiation and Proliferation
52
Differentiation
48
Immortalization/ Senescence/ apoptosis
15
9
5
5
4
4
MYB
SEL1L
TERT
FAM19A2
VOPP1
TCF12
ZC2HC1B
TMCC3
ENSGALT00000007451

B6B Tumor

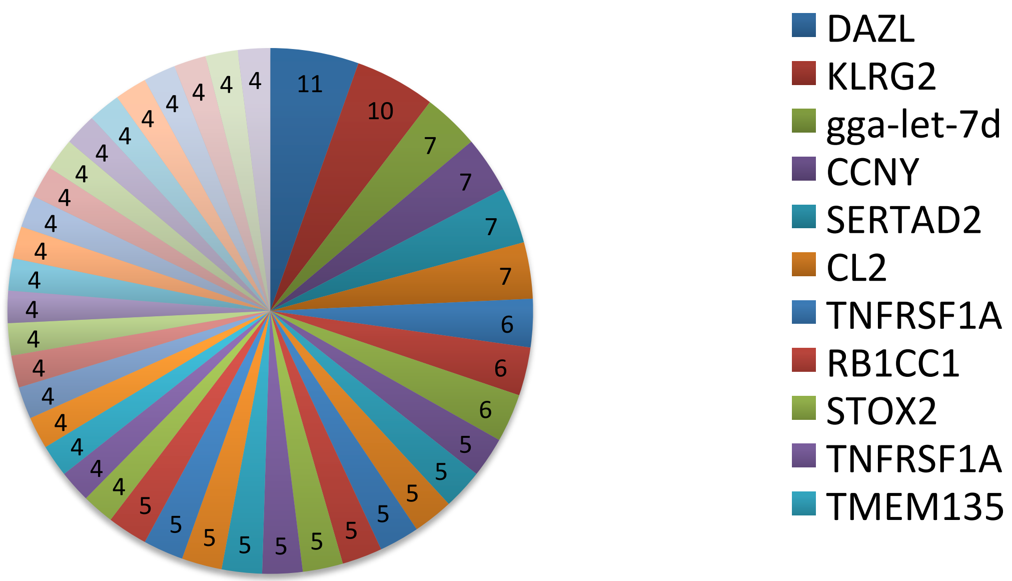

11
10
7
7
7
7
6
6
6
5
5
5
5
5
5
5
5
5
5
5
4
4
4
4
4
4
4
4
4
4
4
4
4
4
4
4
4
4
4
DAZL
KLRG2
gga-let-7d
CCNY
SERTAD2
CL2
TNFRSF1A
RB1CC1
STOX2
TNFRSF1A
TMEM135

B8B Tumor

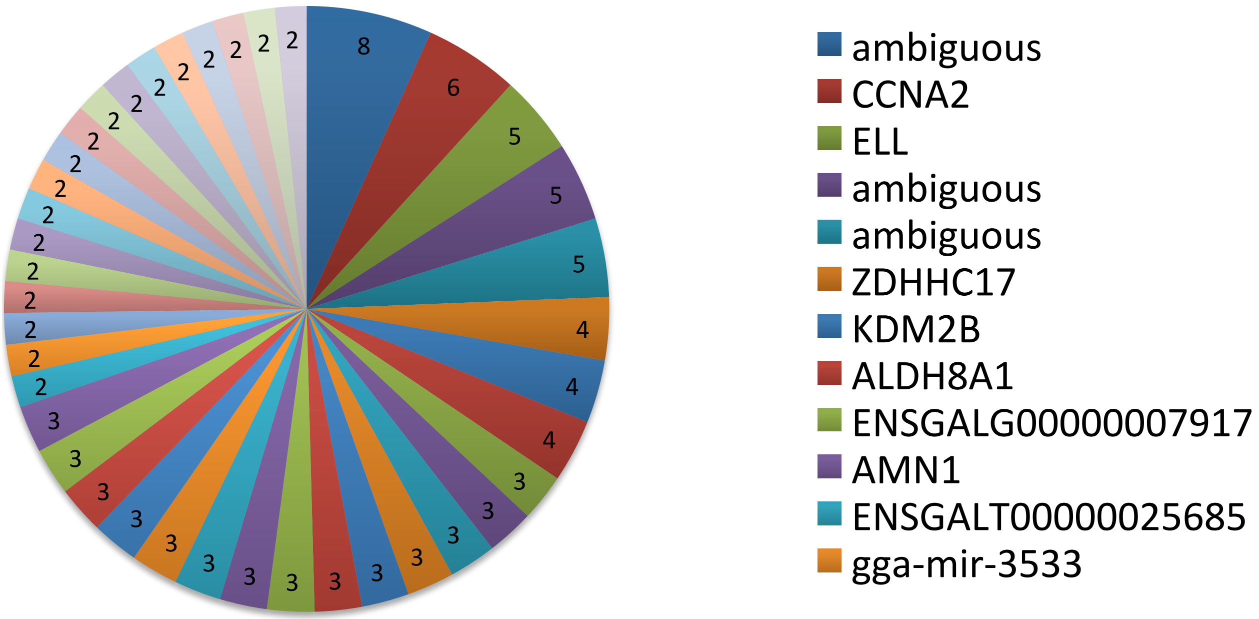

8
6
5
5
5
4
4
4
3
3
3
3
3
3
3
3
3
3
3
3
3
2
2
2
2
2
2
2
2
2
2
2
2
2
2
2
2
2
2
ambiguous
CCNA2
ELL
ambiguous
ambiguous
ZDHHC17
KDM2B
ALDH8A1
ENSGALG00000007917
AMN1
ENSGALT00000025685
gga-mir-3533

C2B Tumor

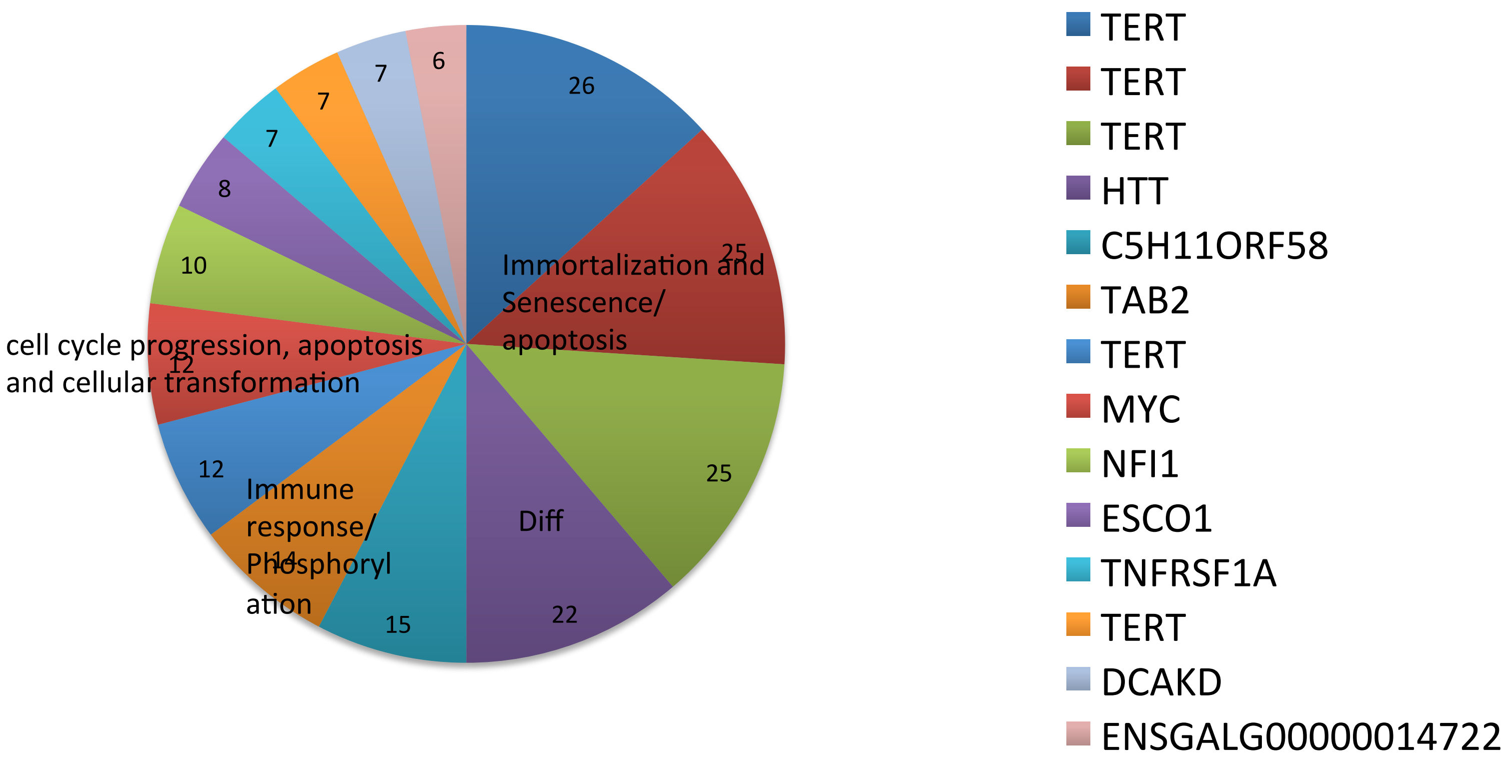
26
25
25
22
15
14
12
12
10
8
7
7
7
6
Immortalization and Senescence/ apoptosis
cell cycle progression, apoptosis and cellular transformation
Immune response/ Phosphorylation
Diff
TERT
TERT
TERT
HTT
C5H11ORF58
TAB2
TERT
MYC
NFI1
ESCO1
TNFRSF1A
TERT
DCAKD
ENSGALG00000014722

C5B Tumor

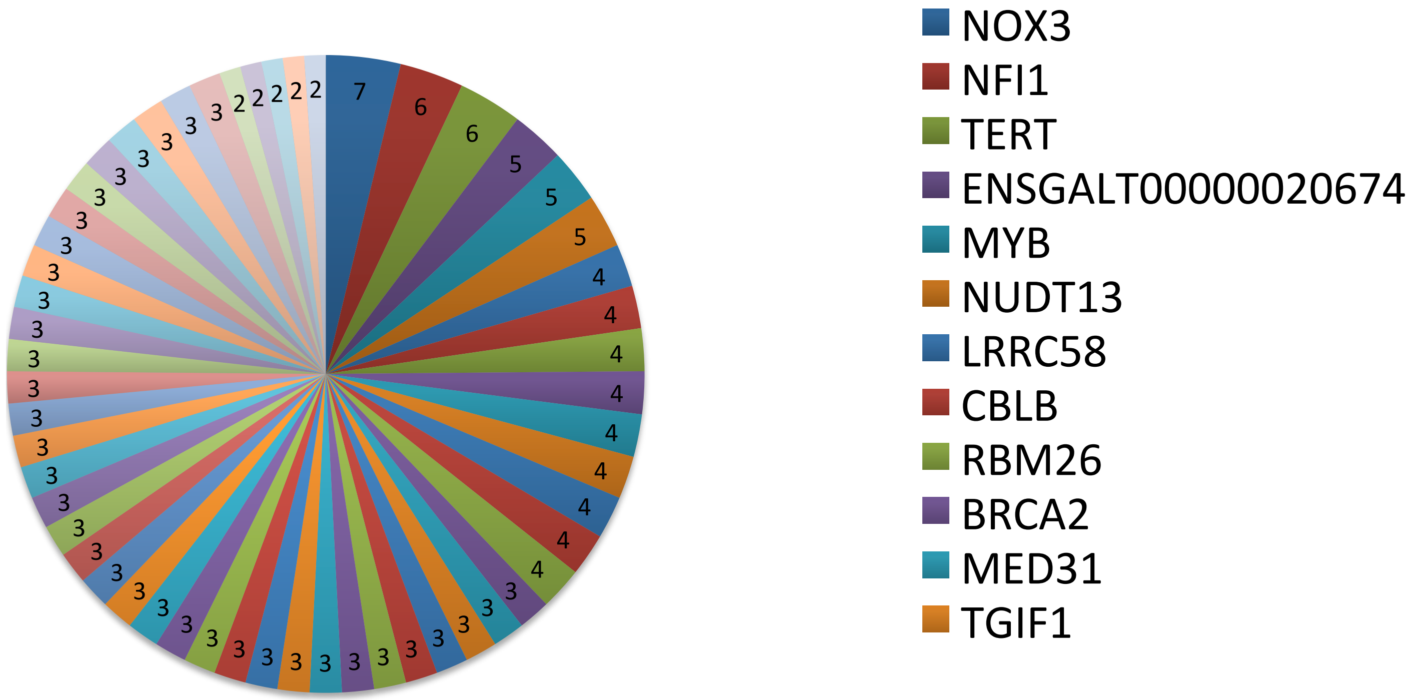
NOX3
NFI1
TERT
ENSGALT00000020674
MYB
NUDT13
LRRC58
CBLB
RBM26
BRCA2
MED31
TGIF1

C6B Tumor

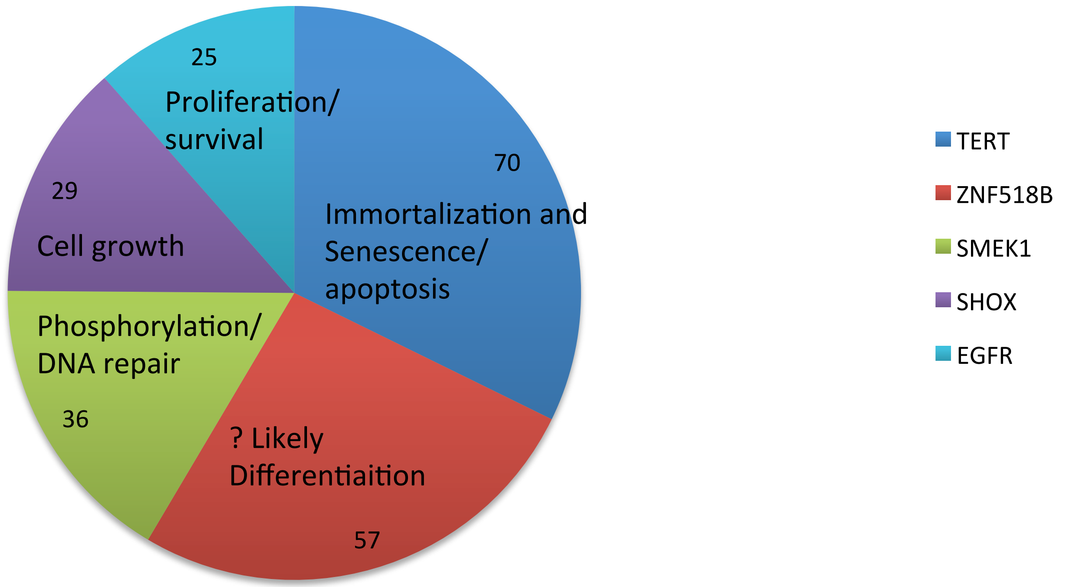

25
Proliferation/ survival
70
Immortalization and Senescence/ apoptosis
29
Cell growth
Phosphorylation/ DNA repair
36
? Likely Differentiaition
57
TERT
ZNF518B
SMEK1
SHOX
EGFR

C7B Tumor

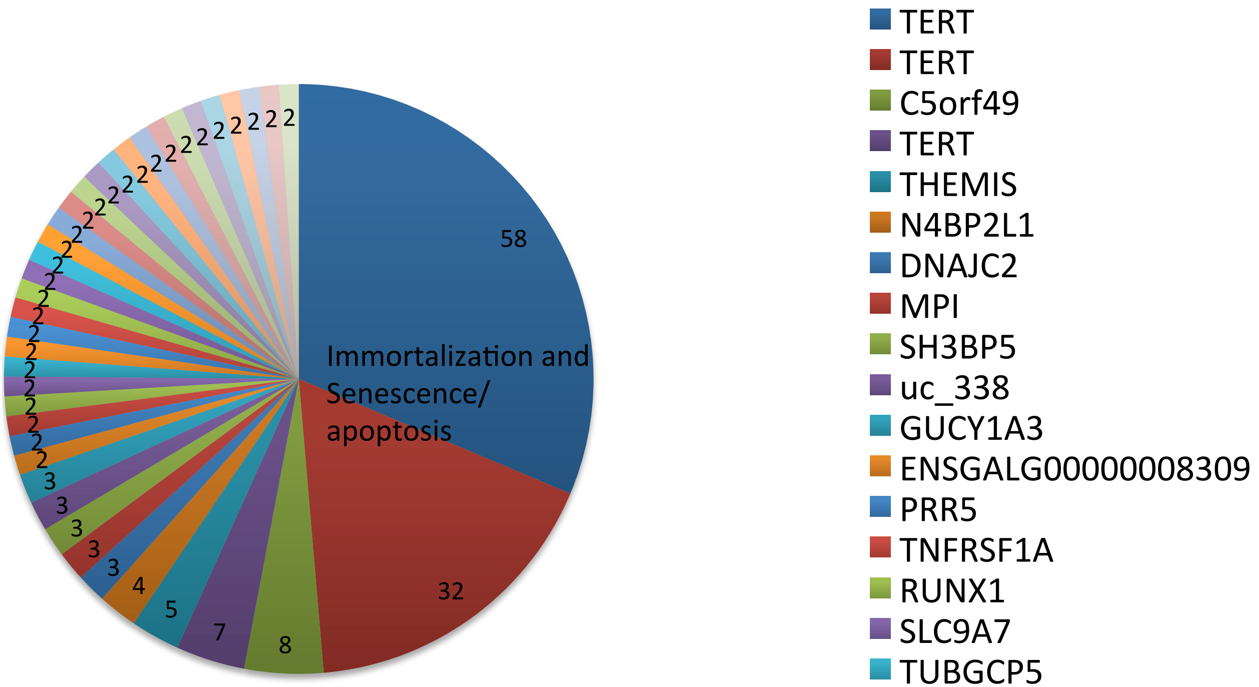
58
32
8
7
5
4
3
3
3
3
3
2
Immortalization and Senescence/ apoptosis
TERT
TERT
C5orf49
TERT
THEMIS
N4BP2L1
DNAJC2
MPI
SH3BP5
uc_338
GUCY1A3
ENSGALG00000008309
PRR5
TNFRSF1A
RUNX1
SLC9A7
TUBGCP5
ENSGALG00000016987

D2B Tumor

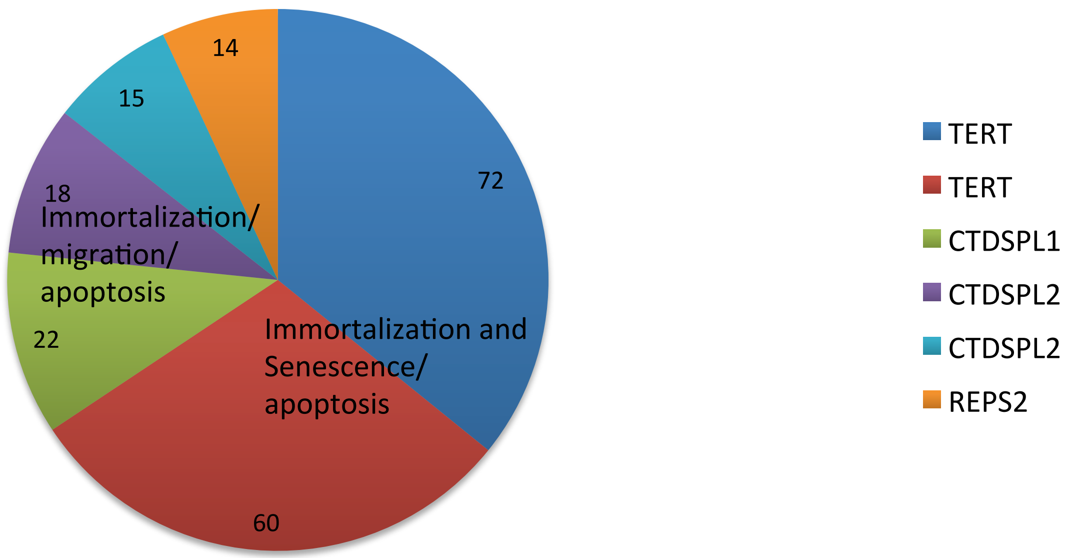

14
15
72
18
Immortalization/
migration/
apoptosis
22
Immortalization and
Senescence/
apoptosis
60
TERT
TERT
CTDSPL1
CTDSPL2
CTDSPL2
REPS2

D4B Tumor

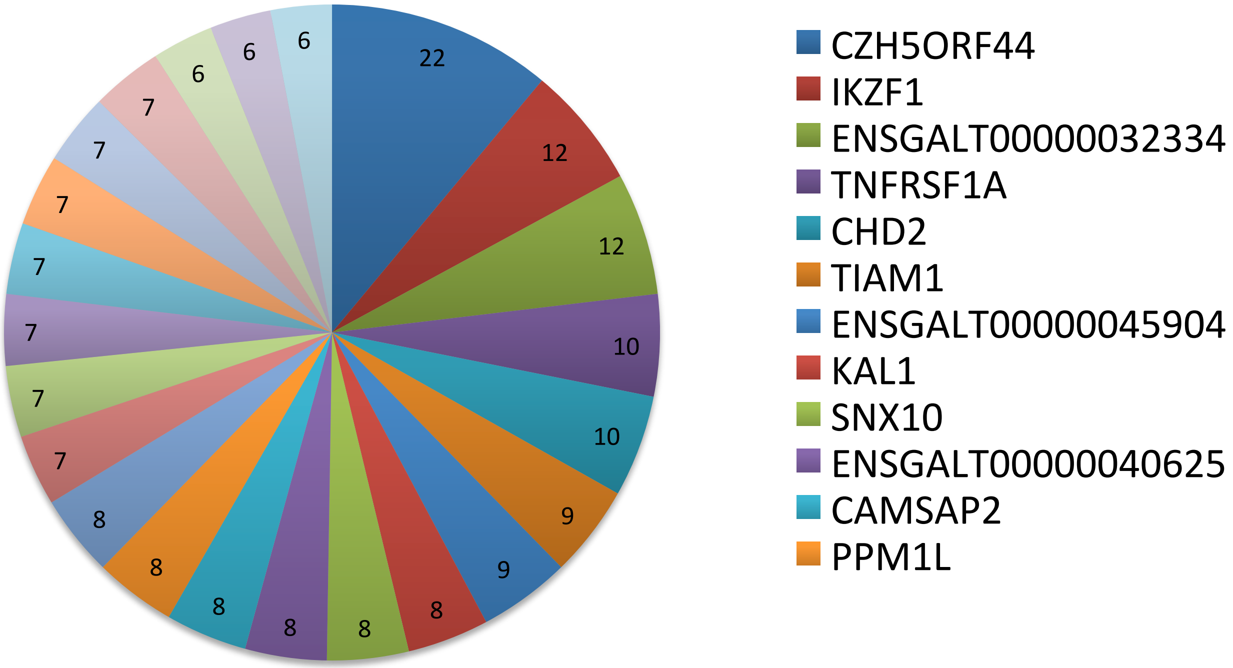

22
12
12
10
10
9
9
8
8
8
8
8
8
7
7
7
7
7
7
7
6
6
6
CZH5ORF44
IKZF1
ENSGALT00000032334
TNFRSF1A
CHD2
TIAM1
ENSGALT00000045904
KAL1
SNX10
ENSGALT00000040625
CAMSAP2
PPM1L

D5B Tumor

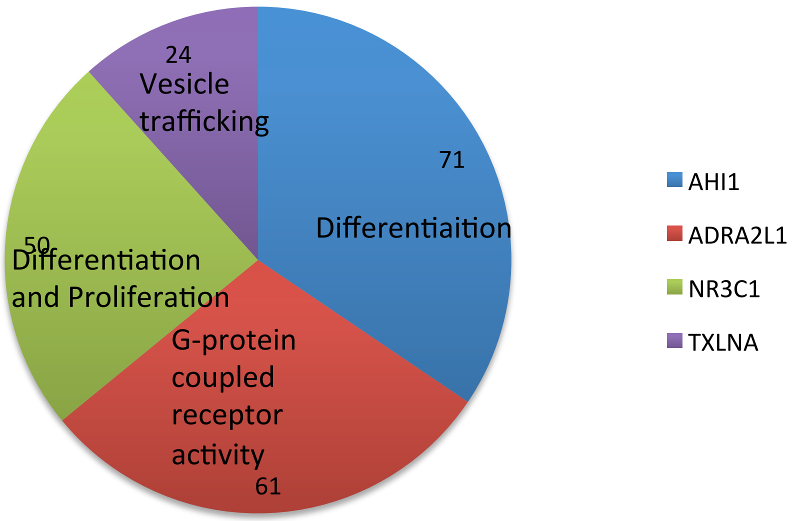
24
Vesicle trafficking
71
Differentiaition
50
Differentiation and Proliferation
G-protein coupled receptor activity
61
AHI1
ADRA2L1
NR3C1
TXLNA

D7B Tumor

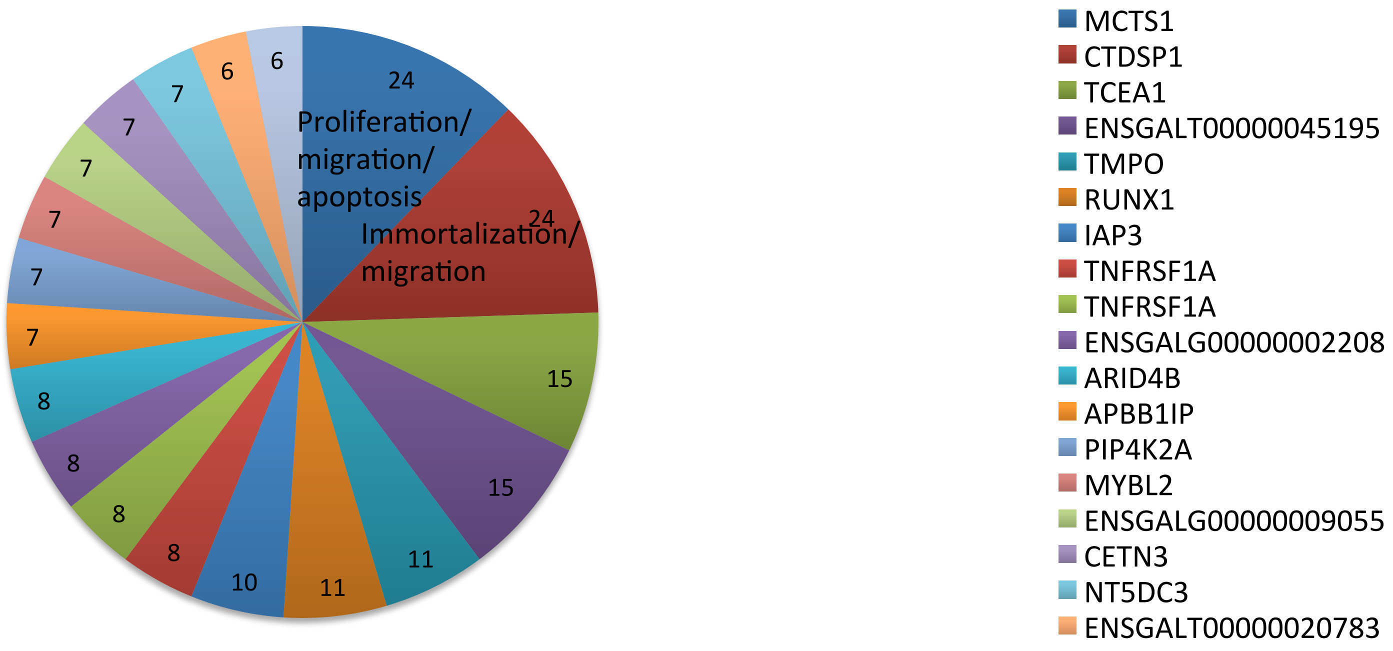

24
Proliferation/
migration/
apoptosis
24
Immortalization/
migration
15
15
11
11
10
8
8
8
8
7
7
7
7
7
7
6
6
MCTS1
CTDSP1
TCEA1
ENSGALT00000045195
TMPO
RUNX1
IAP3
TNFRSF1A
TNFRSF1A
ENSGALG00000002208
ARID4B
APBB1IP
PIP4K2A
MYBL2
ENSGALG00000009055
CETN3
NT5DC3
ENSGALT00000020783

D9B Tumor

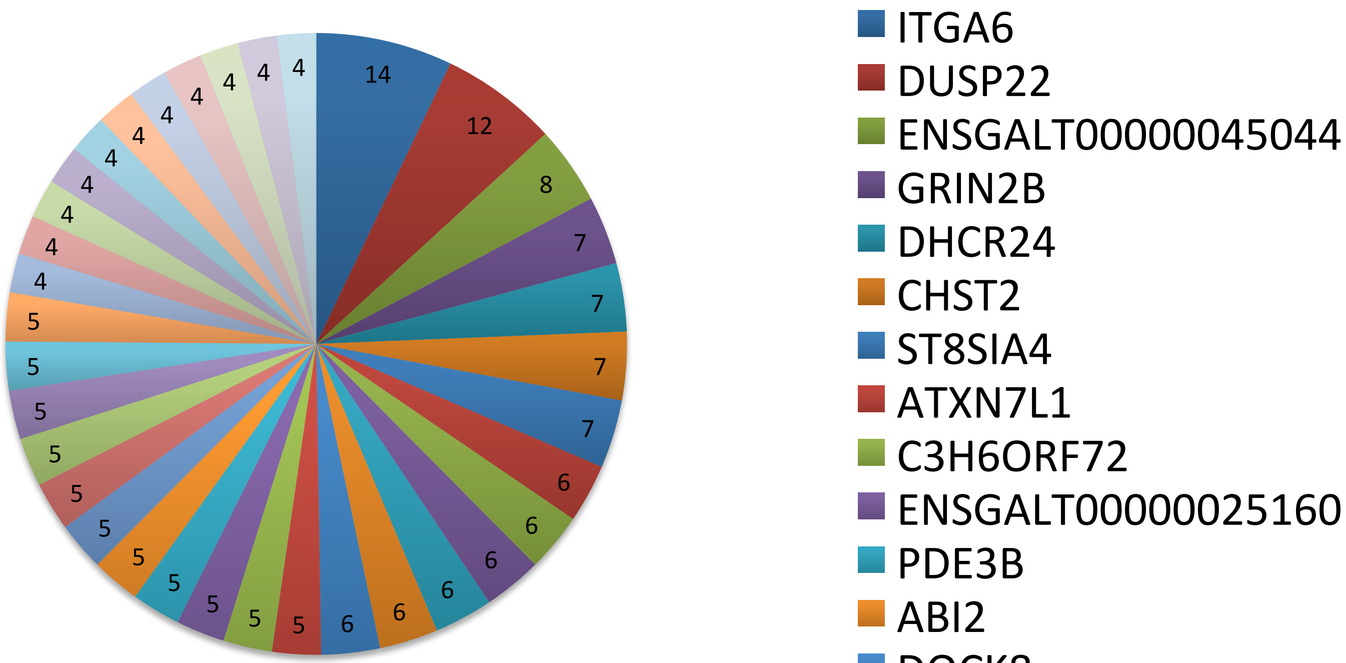

14
12
8
7
7
7
7
6
6
6
6
6
6
5
5
5
5
5
5
5
5
5
5
5
4
4
4
4
4
4
4
4
4
4
4
ITGA6
DUSP22
ENSGALT00000045044
GRIN2B
DHCR24
CHST2
ST8SIA4
ATXN7L1
C3H6ORF72
ENSGALT00000025160
PDE3B
ABI2
DOCK8

D8B Tumor

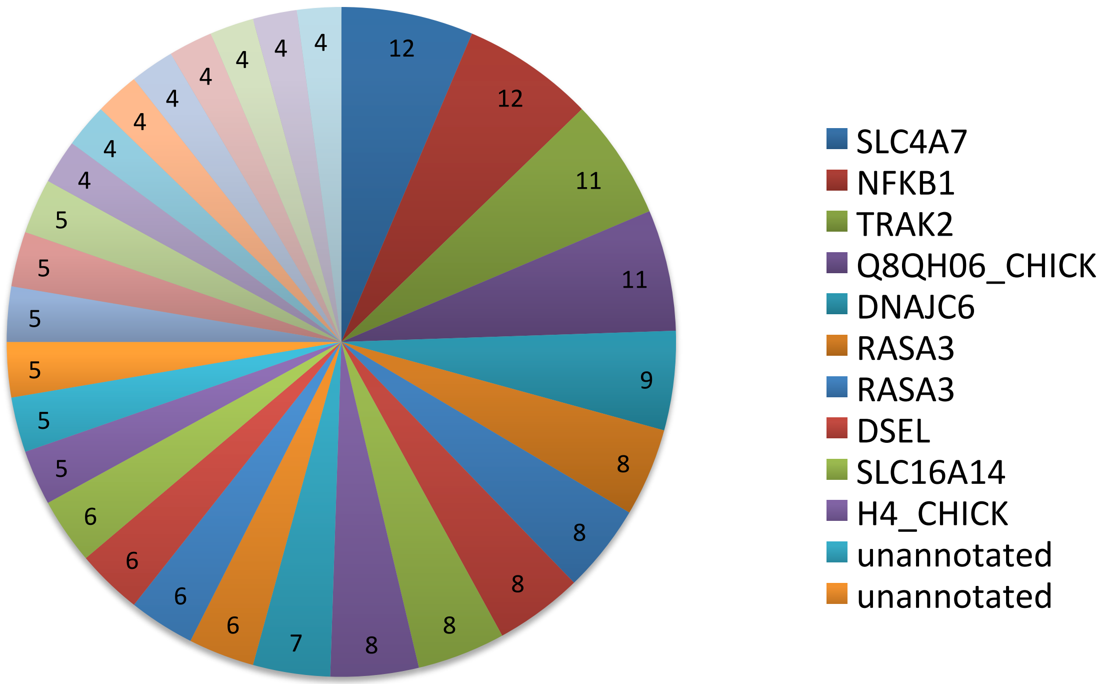
12
12
11
11
9
8
8
8
8
8
7
6
6
6
6
5
5
5
5
5
5
4
4
4
4
4
4
4
4
SLC4A7
NFKB1
TRAK2
Q8QH06_CHICK
DNAJC6
RASA3
RASA3
DSEL
SLC16A14
H4_CHICK
unannotated
unannotated

E5B Tumor

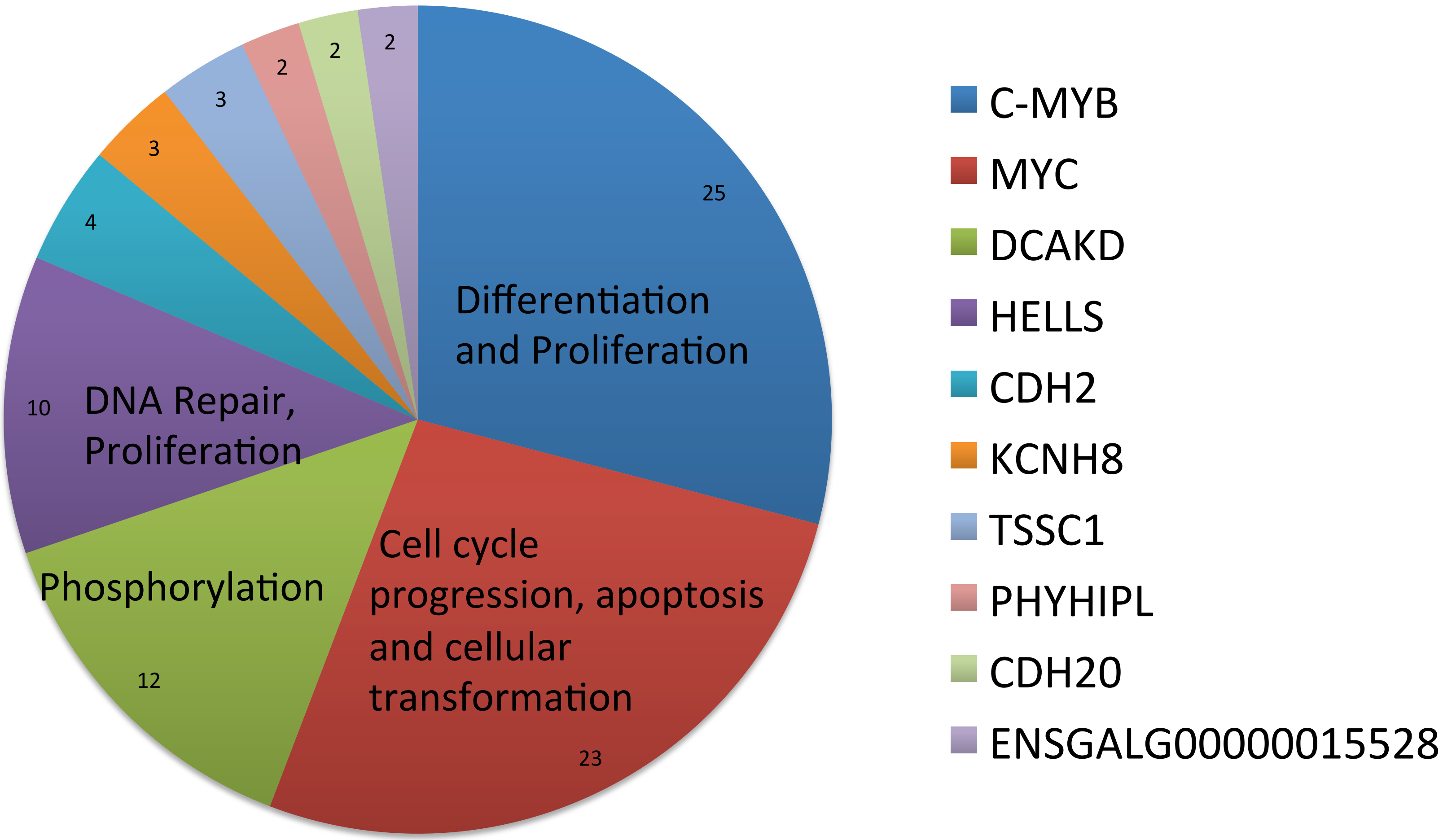
25
Differentiation and Proliferation
23
Cell cycle progression, apoptosis and cellular transformation
12
Phosphorylation
10
DNA Repair, Proliferation
4
3
3
2
2
2
C-MYB
MYC
DCAKD
HELLS
CDH2
KCNH8
TSSC1
PHYHIPL
CDH20
ENSGALG00000015528

F1B Tumor

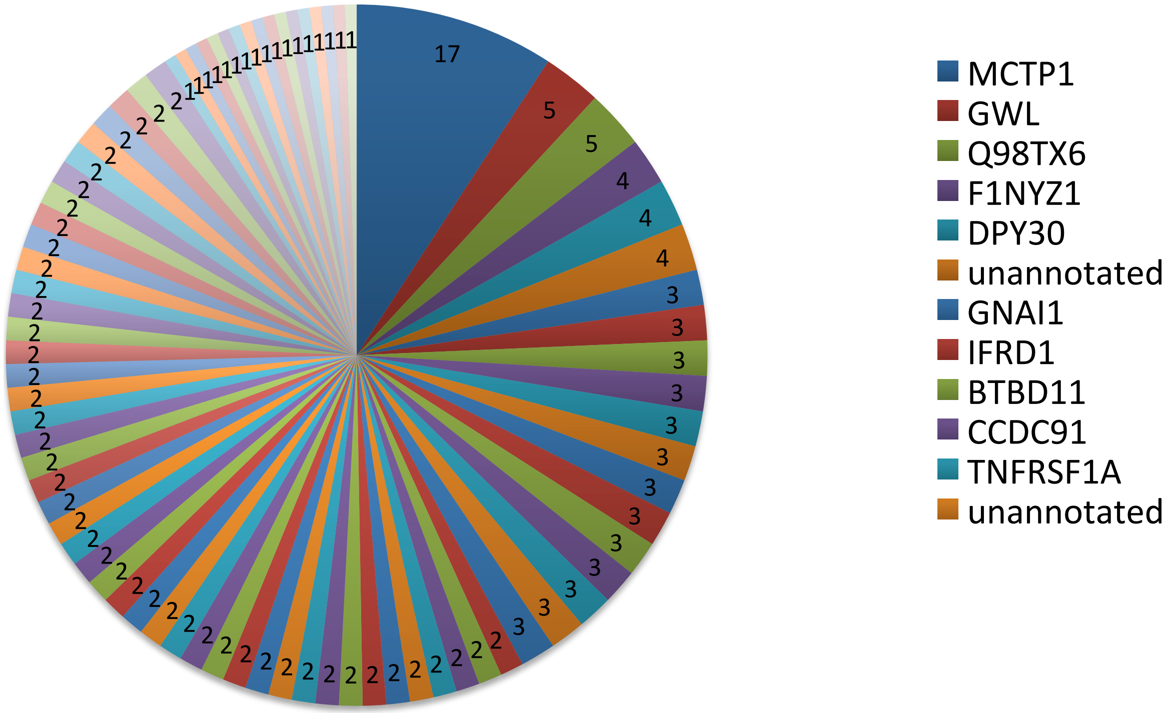
MCTP1
GWL
Q98TX6
F1NYZ1
DPY30
unannotated
GNAI1
IFRD1
BTBD11
CCDC91
TNFRSF1A
unannotated

F2B Tumor

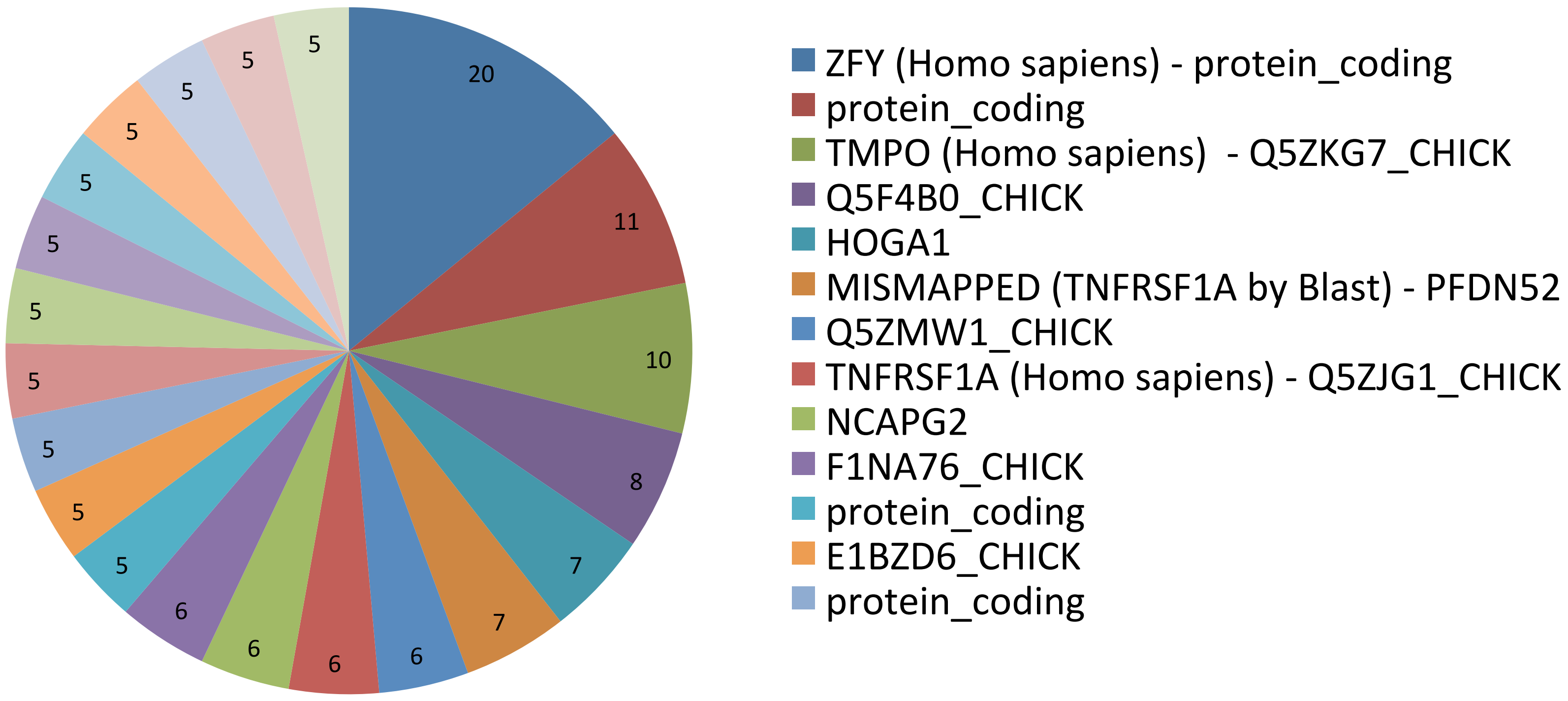
20
11
10
8
7
7
6
6
6
6
5
5
5
5
5
5
5
5
5
5
5
ZFY (Homo sapiens) - protein_coding
protein_coding
TMPO (Homo sapiens) - Q5ZKG7_CHICK
Q5F4B0_CHICK
HOGA1
MISMAPPED (TNFRSF1A by Blast) - PFDN52
Q5ZMW1_CHICK
TNFRSF1A (Homo sapiens) - Q5ZJG1_CHICK
NCAPG2
F1NA76_CHICK
protein_coding
E1BZD6_CHICK
protein_coding

F3B Tumor

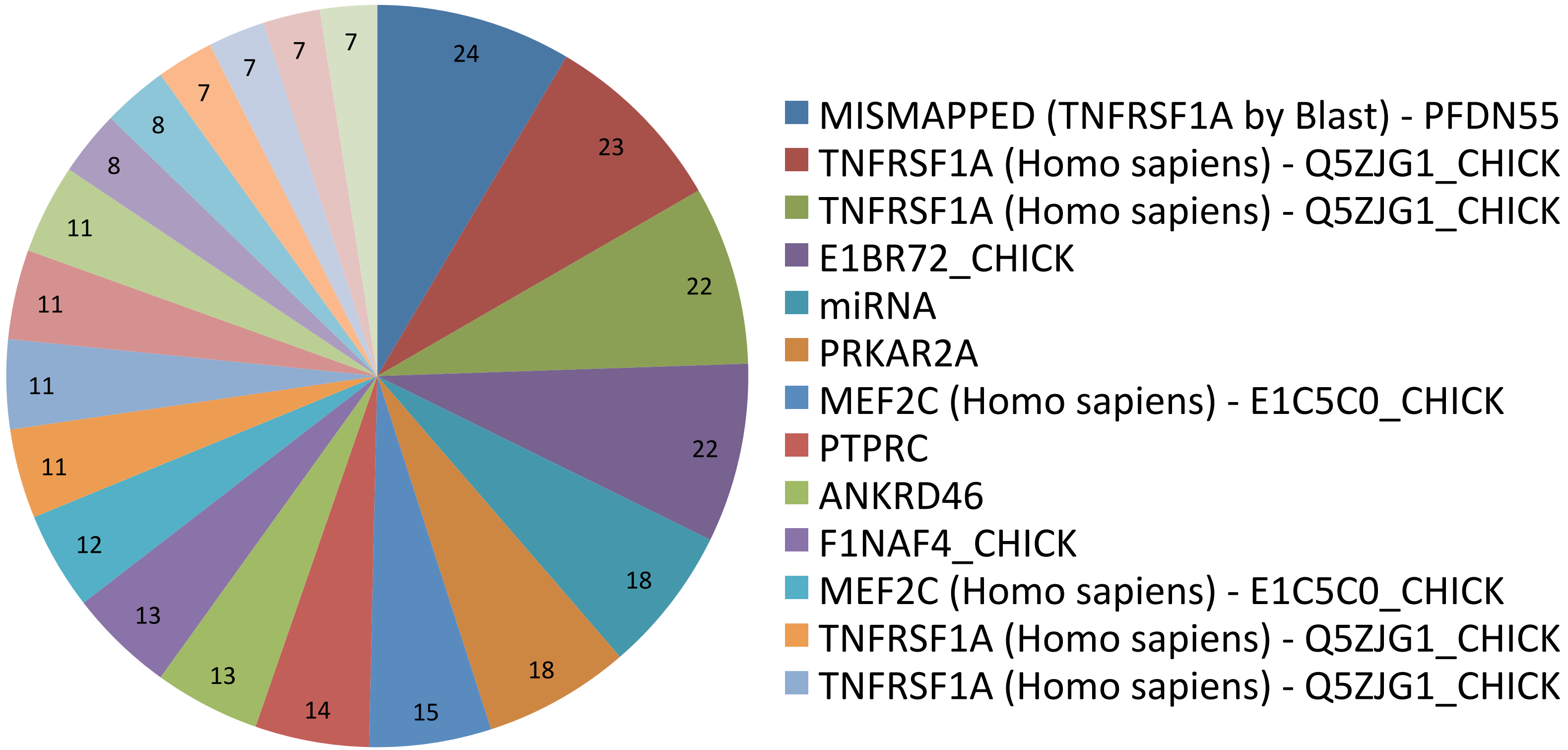
24
23
22
22
18
18
15
14
13
13
12
11
11
11
11
8
8
7
7
7
7
MISMAPPED (TNFRSF1A by Blast) - PFDN55
TNFRSF1A (Homo sapiens) - Q5ZJG1_CHICK
TNFRSF1A (Homo sapiens) - Q5ZJG1_CHICK
E1BR72_CHICK
miRNA
PRKAR2A
MEF2C (Homo sapiens) - E1C5C0_CHICK
PTPRC
ANKRD46
F1NAF4_CHICK
MEF2C (Homo sapiens) - E1C5C0_CHICK
TNFRSF1A (Homo sapiens) - Q5ZJG1_CHICK
TNFRSF1A (Homo sapiens) - Q5ZJG1_CHICK

F4B Tumor

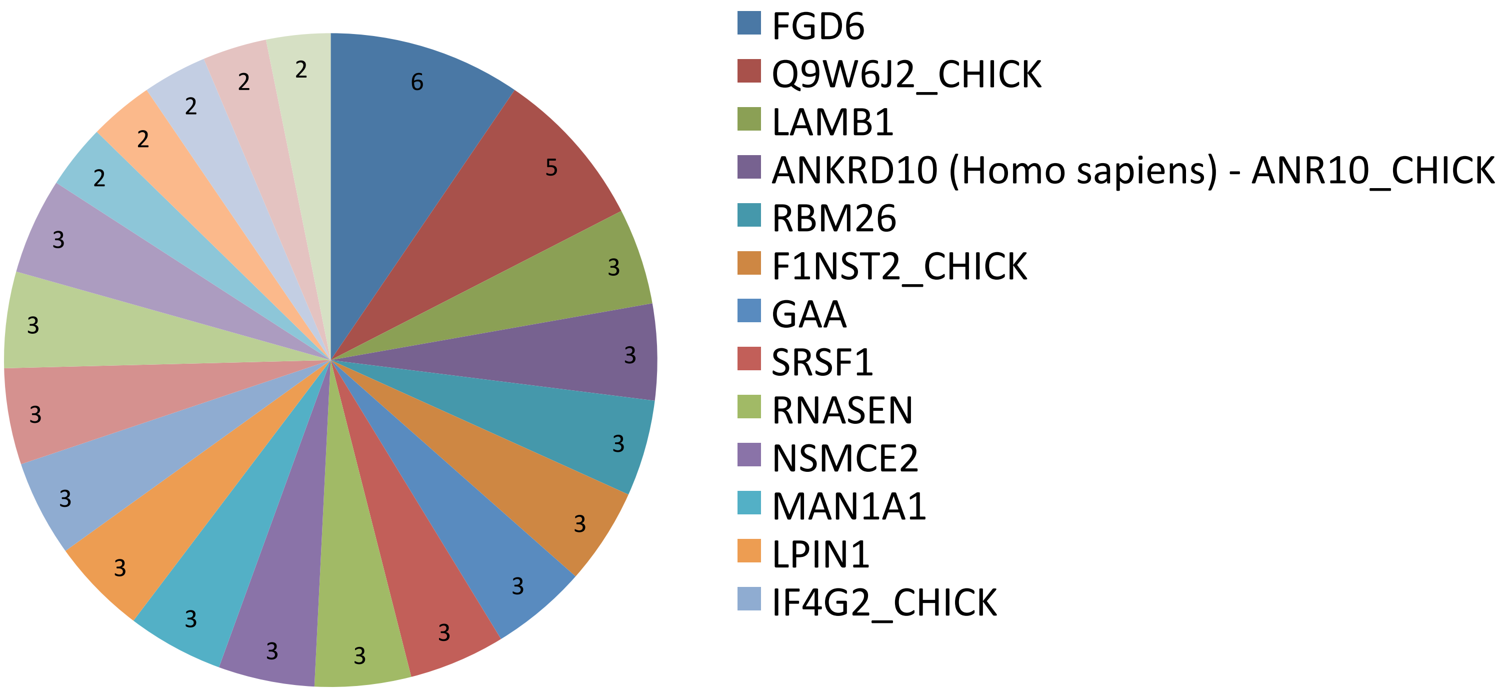
6
5
3
3
3
3
3
3
3
3
3
3
3
3
3
2
2
2
2
2
FGD6
Q9W6J2_CHICK
LAMB1
ANKRD10 (Homo sapiens) - ANR10_CHICK
RBM26
F1NST2_CHICK
GAA
SRSF1
RNASEN
NSMCE2
MAN1A1
LPIN1
IF4G2_CHICK

F5B Tumor

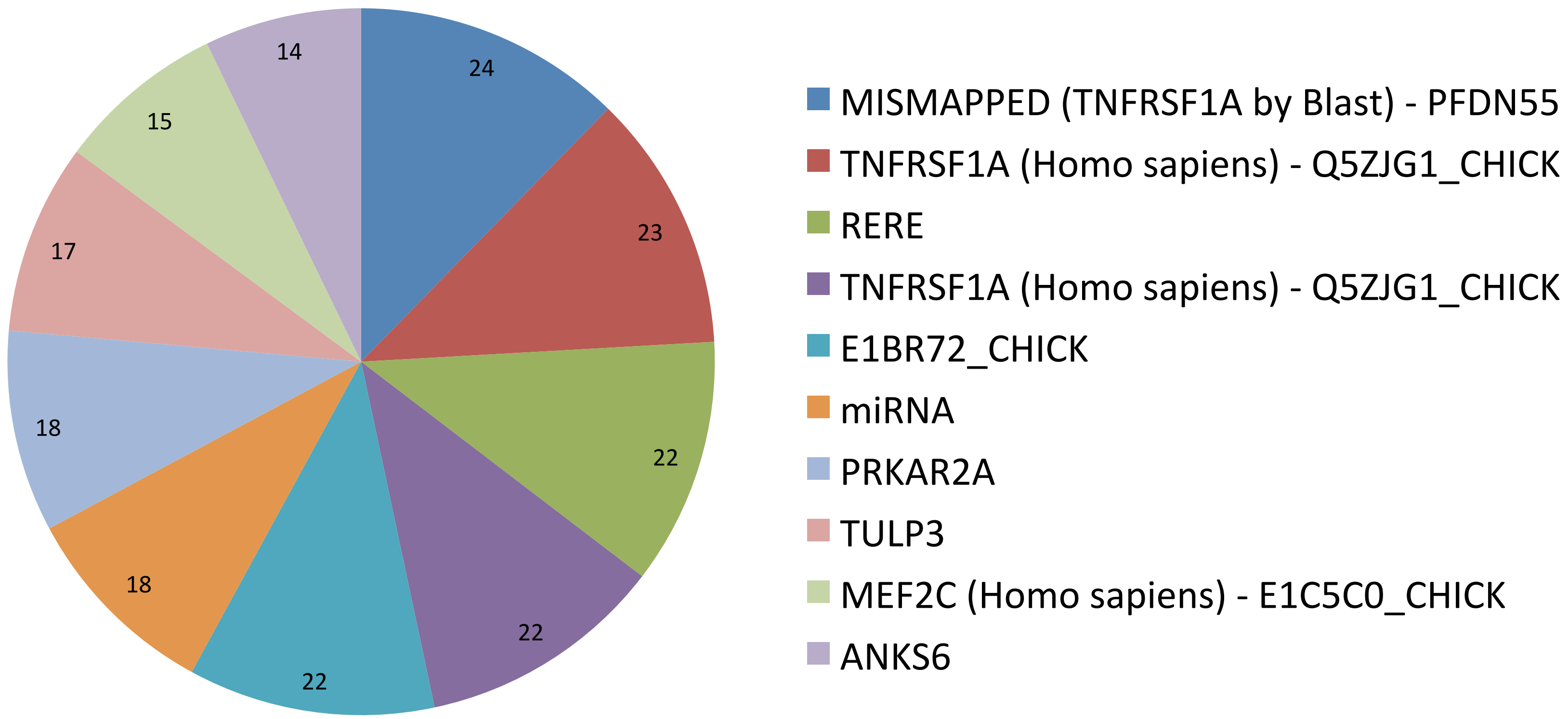
24
23
22
22
22
18
18
17
15
14
MISMAPPED (TNFRSF1A by Blast) - PFDN55
TNFRSF1A (Homo sapiens) - Q5ZJG1_CHICK
RERE
TNFRSF1A (Homo sapiens) - Q5ZJG1_CHICK
E1BR72_CHICK
miRNA
PRKAR2A
TULP3
MEF2C (Homo sapiens) - E1C5C0_CHICK
ANKS6

Liver

A2L Tumor

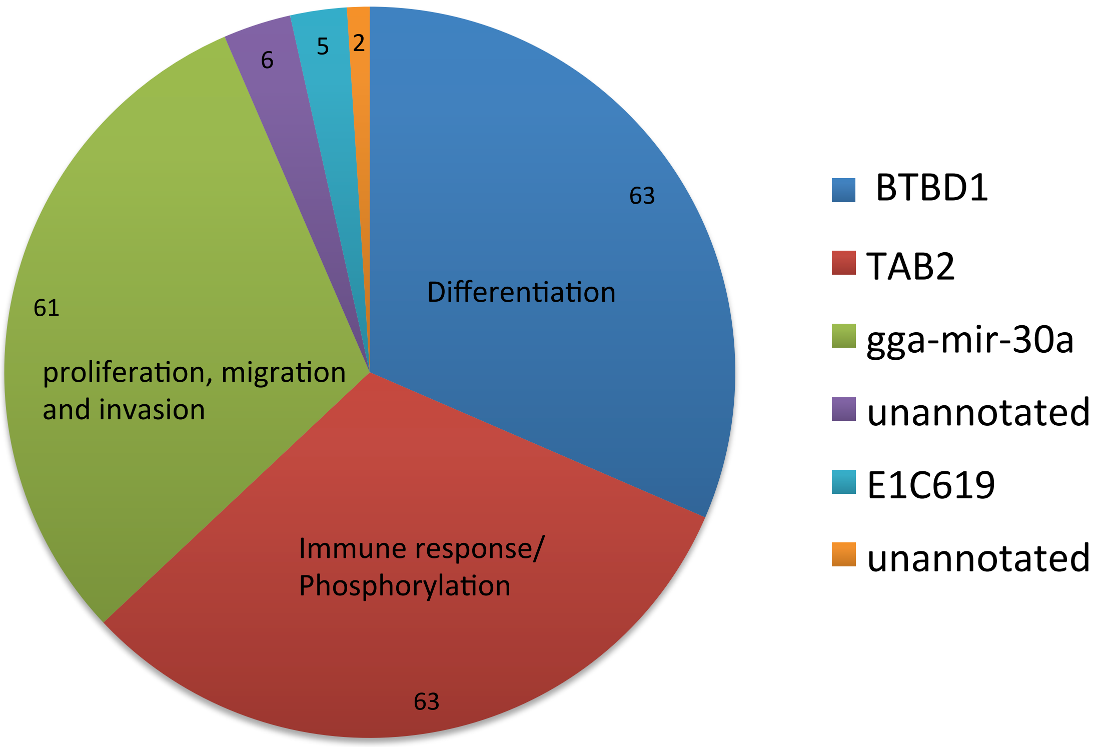

6
5
2
63
Differentiation
61
proliferation, migration and invasion
Immune response/ Phosphorylation
63
BTBD1
TAB2
gga-mir-30a
unannotated
E1C619
unannotated

A4L Tumor

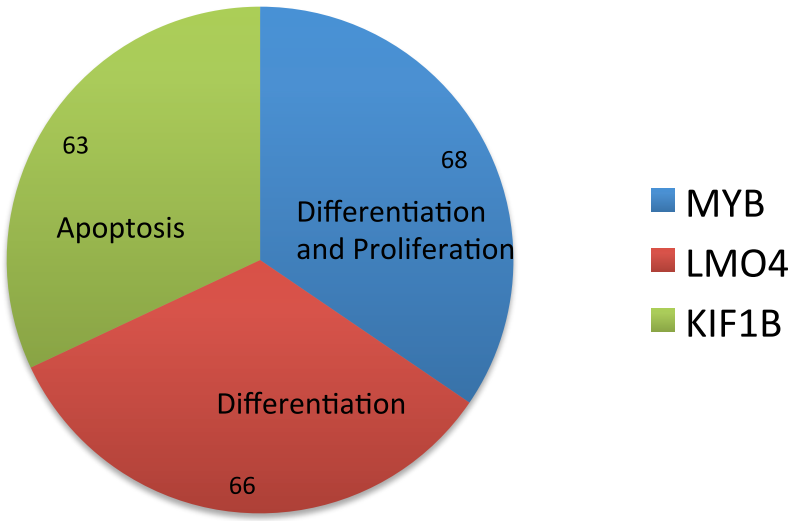

63
Apoptosis
68
Differentiation and Proliferation
Differentiation
66
MYB
LMO4
KIF1B

A5L Tumor

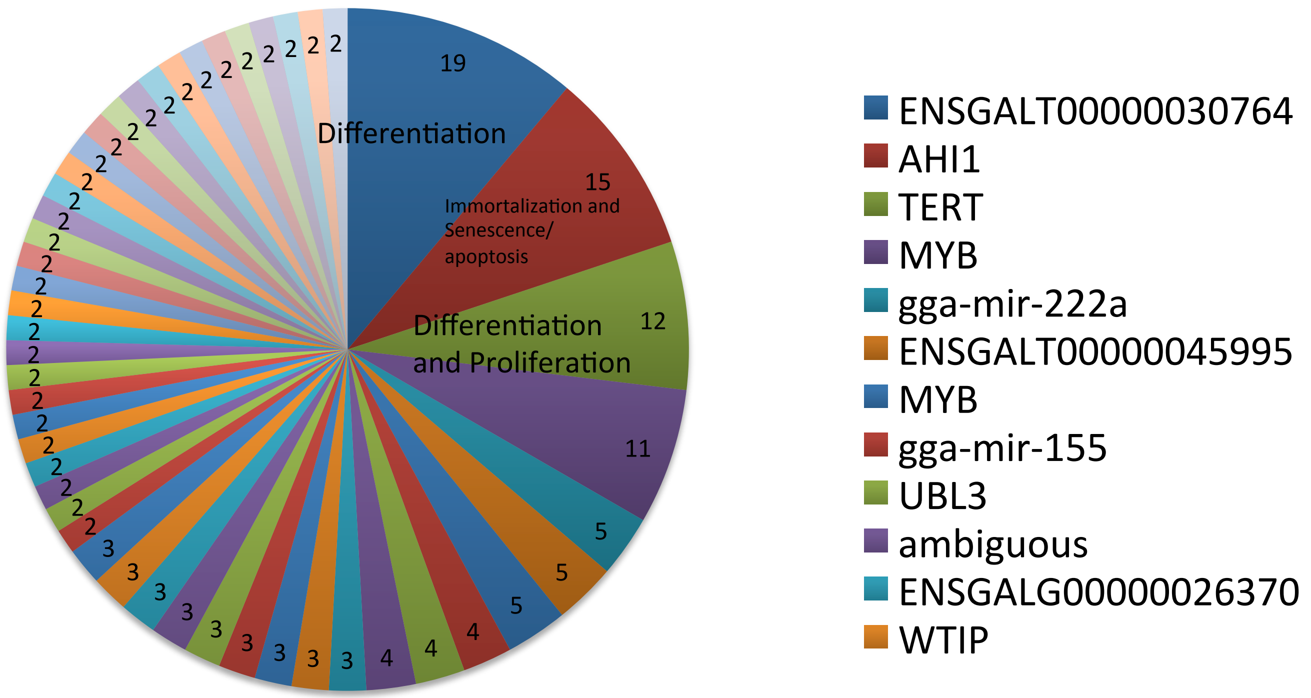
19
Differentiation
15
Immortalization and
Senescence/
apoptosis
12
Differentiation
and Proliferation
11
5
5
5
4
4
4
3
3
3
3
3
3
3
3
3
2
ENSGALT00000030764
AHI1
TERT
MYB
gga-mir-222a
ENSGALT00000045995
MYB
gga-mir-155
UBL3
ambiguous
ENSGALG00000026370
WTIP

C2L Tumor

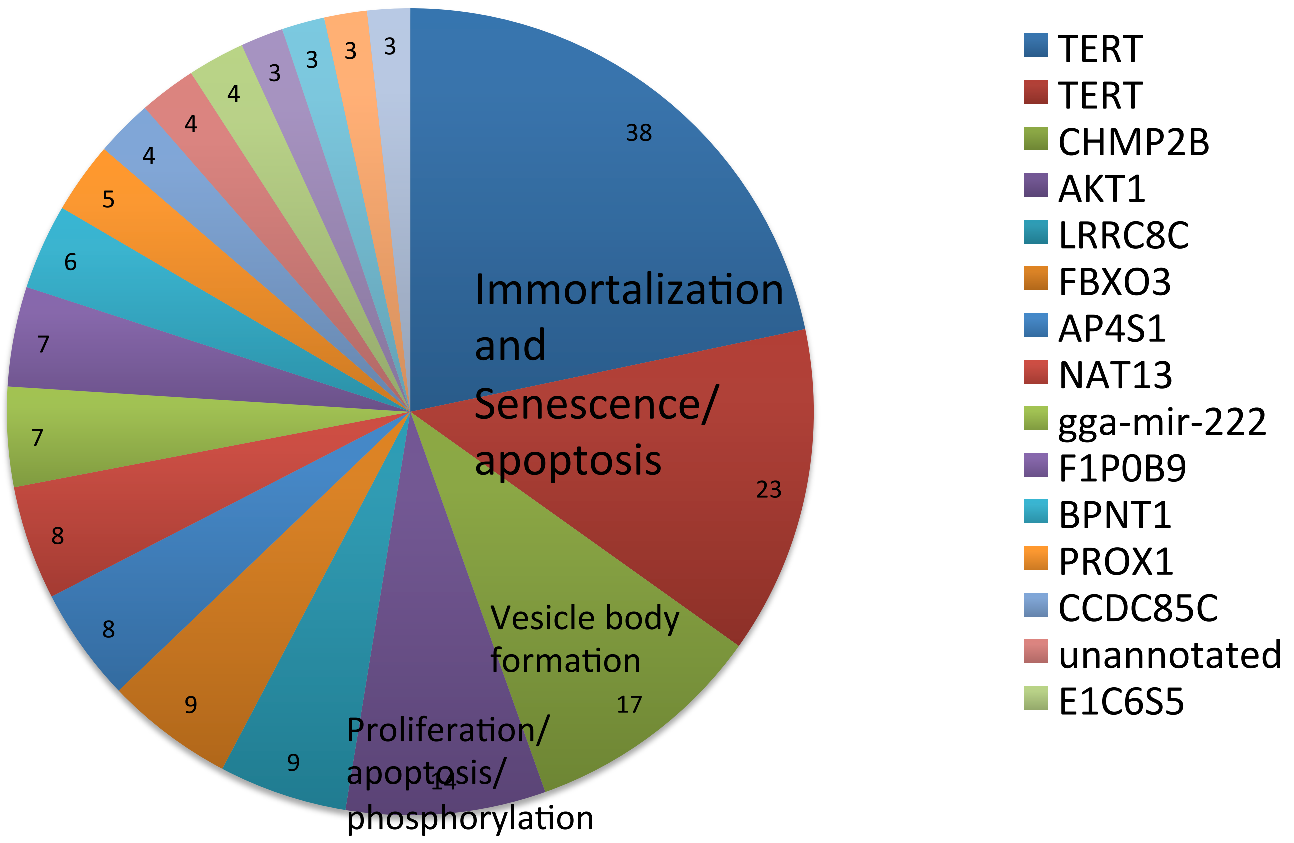

38
23
17
14
9
9
8
8
7
7
6
5
4
4
4
3
3
3
3
Immortalization and Senescence/ apoptosis
Vesicle body formation
Proliferation/ apoptosis/ phosphorylation
TERT
TERT
CHMP2B
AKT1
LRRC8C
FBXO3
AP4S1
NAT13
gga-mir-222
F1P0B9
BPNT1
PROX1
CCDC85C
unannotated
E1C6S5

C3L Tumor

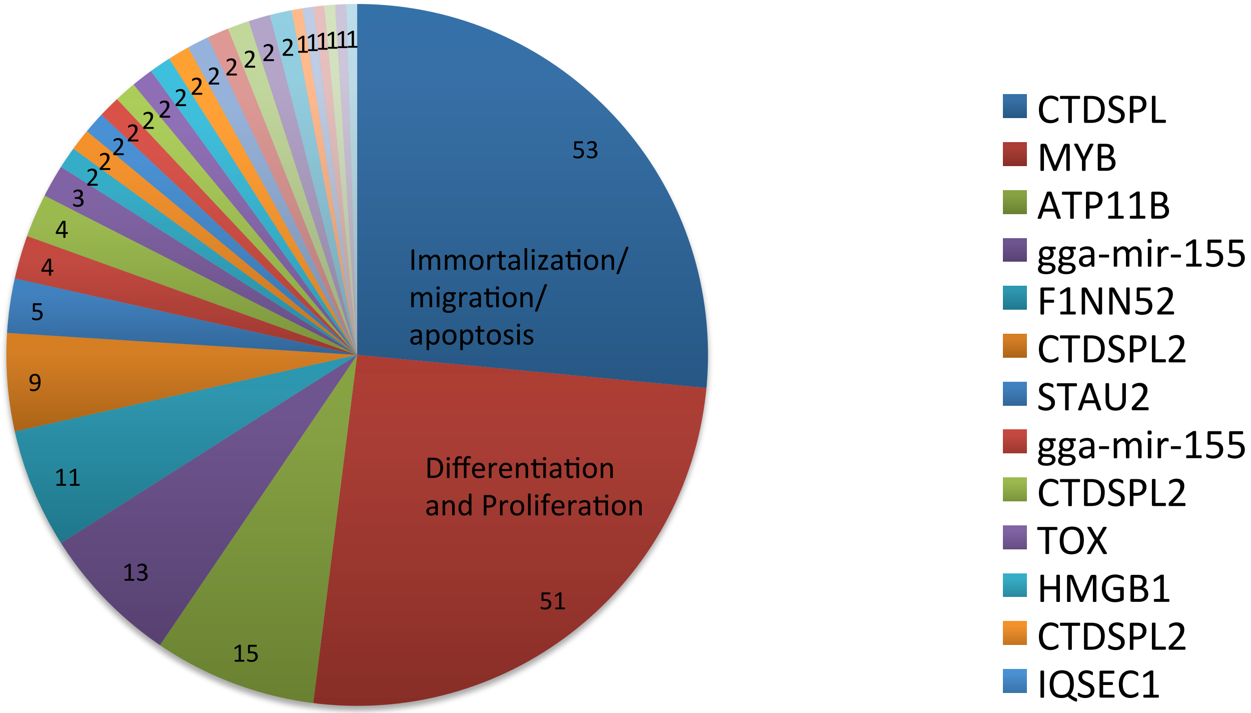
53
Immortalization/ migration/ apoptosis
Differentiation and Proliferation
51
15
13
11
9
5
4
4
3
2
2
2
2
2
2
2
2
2
2
2
2
2
1
1
1
1
1
1
CTDSPL
MYB
ATP11B
gga-mir-155
F1NN52
CTDSPL2
STAU2
gga-mir-155
CTDSPL2
TOX
HMGB1
CTDSPL2
IQSEC1

C4L Tumor

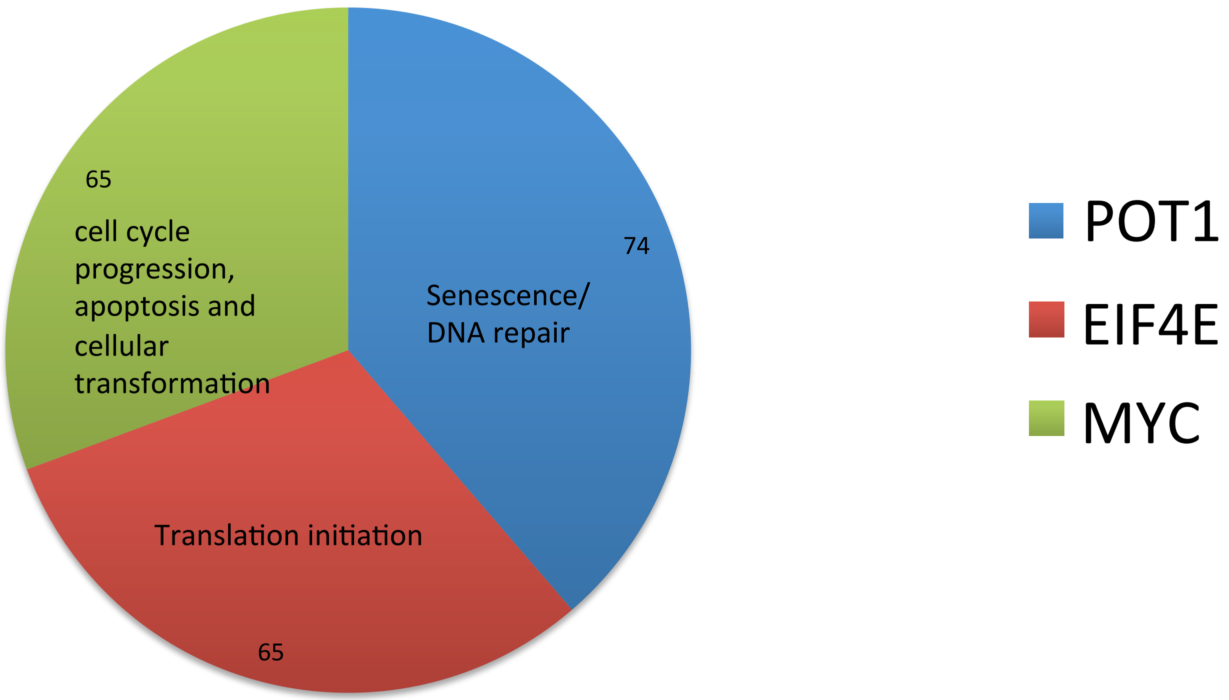

65
cell cycle progression, apoptosis and cellular transformation
74
Senescence/ DNA repair
Translation initiation
65
POT1
EIF4E
MYC

C6L Tumor

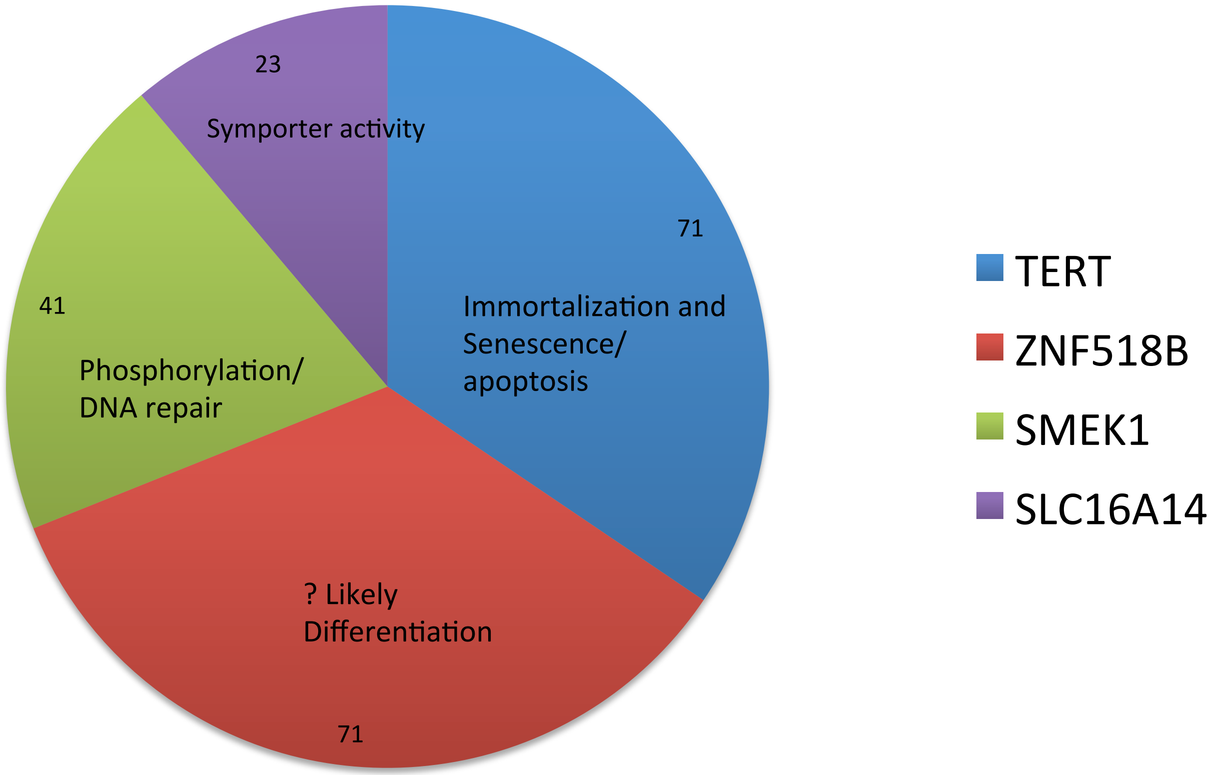
23
Symporter activity
71
Immortalization and
Senescence/
apoptosis
41
Phosphorylation/
DNA repair
? Likely
Differentiation
71
TERT
ZNF518B
SMEK1
SLC16A14

C7L Tumor

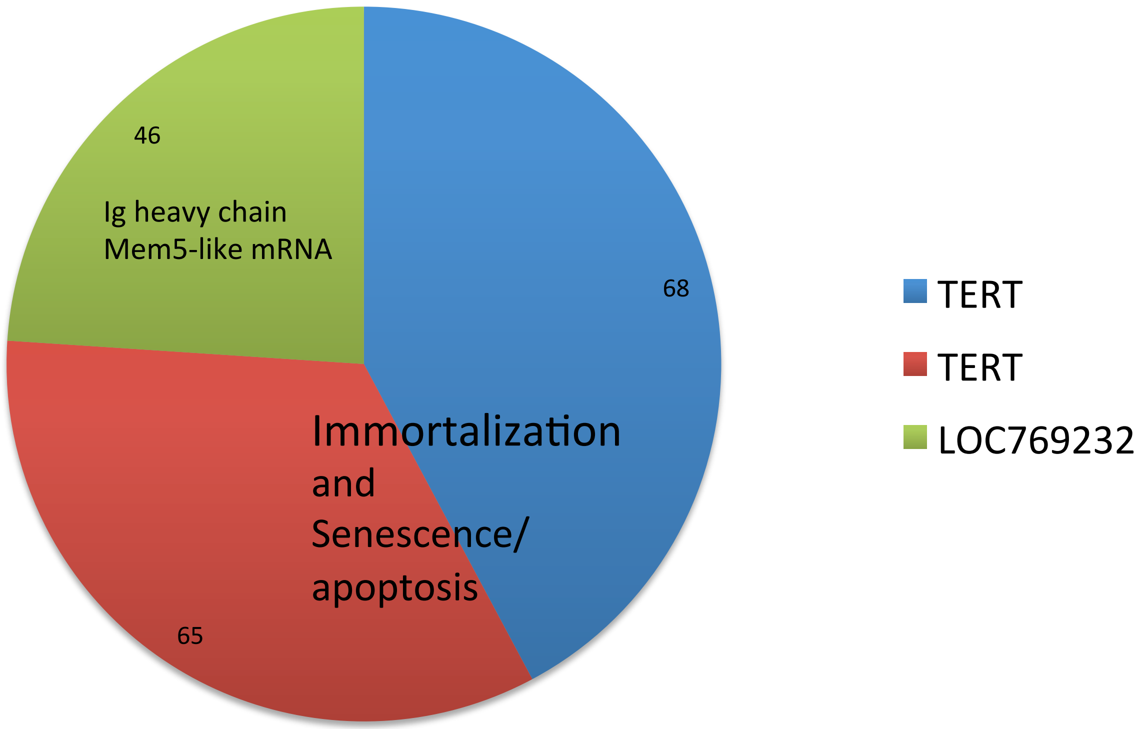
46
Ig heavy chain
Mem5-like mRNA
68
Immortalization
and
Senescence/
apoptosis
65
TERT
TERT
LOC769232

D5L Tumor

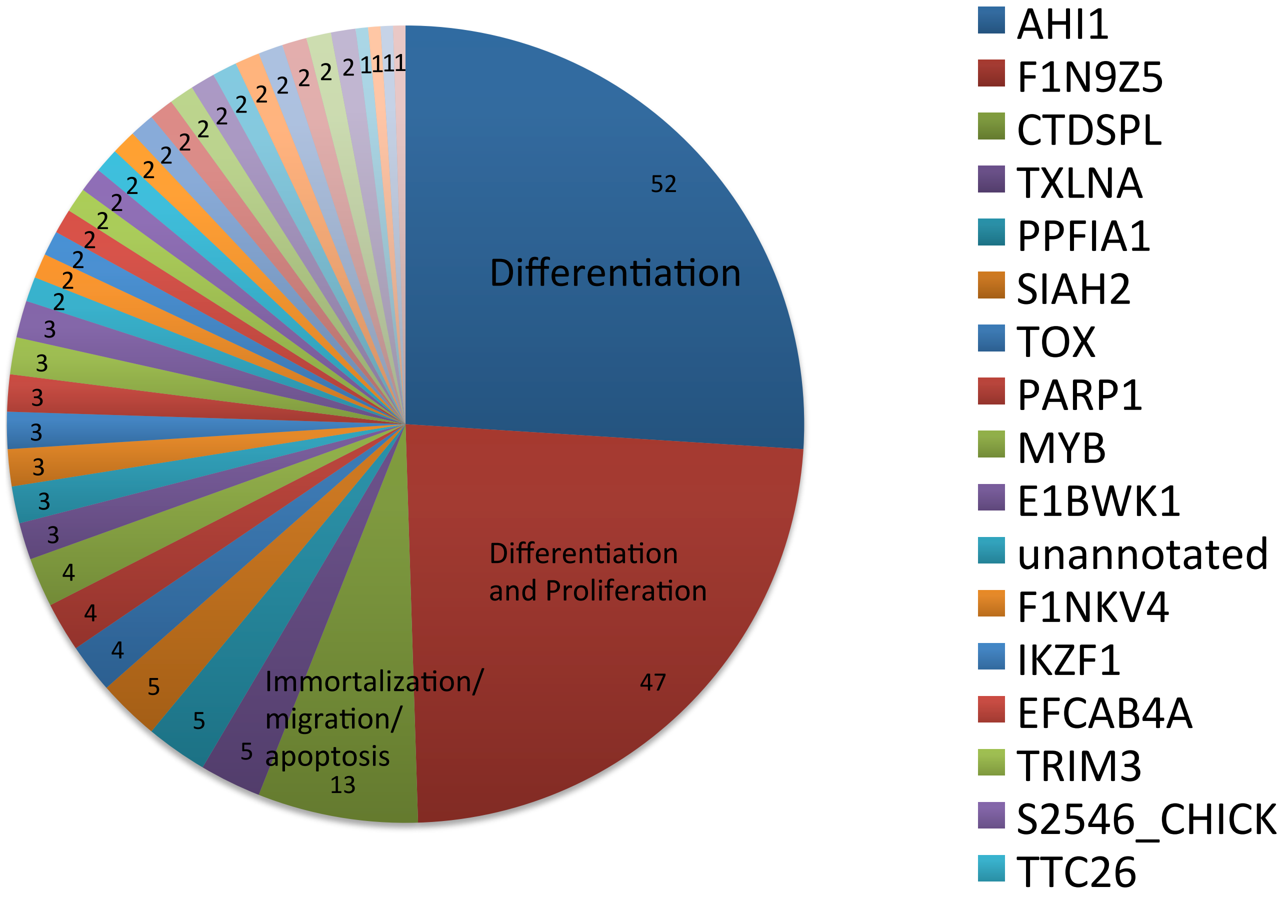

52
Differentiation
Differentiation and Proliferation
47
Immortalization/ migration/ apoptosis
13
5
5
5
4
4
4
3
3
3
3
3
3
3
2
2
2
2
2
2
2
2
2
2
2
2
2
2
2
2
1
1
1
1
AHI1
F1N9Z5
CTDSPL
TXLNA
PPFIA1
SIAH2
TOX
PARP1
MYB
E1BWK1
unannotated
F1NKV4
IKZF1
EFCAB4A
TRIM3
S2546_CHICK
TTC26

D2L Tumor

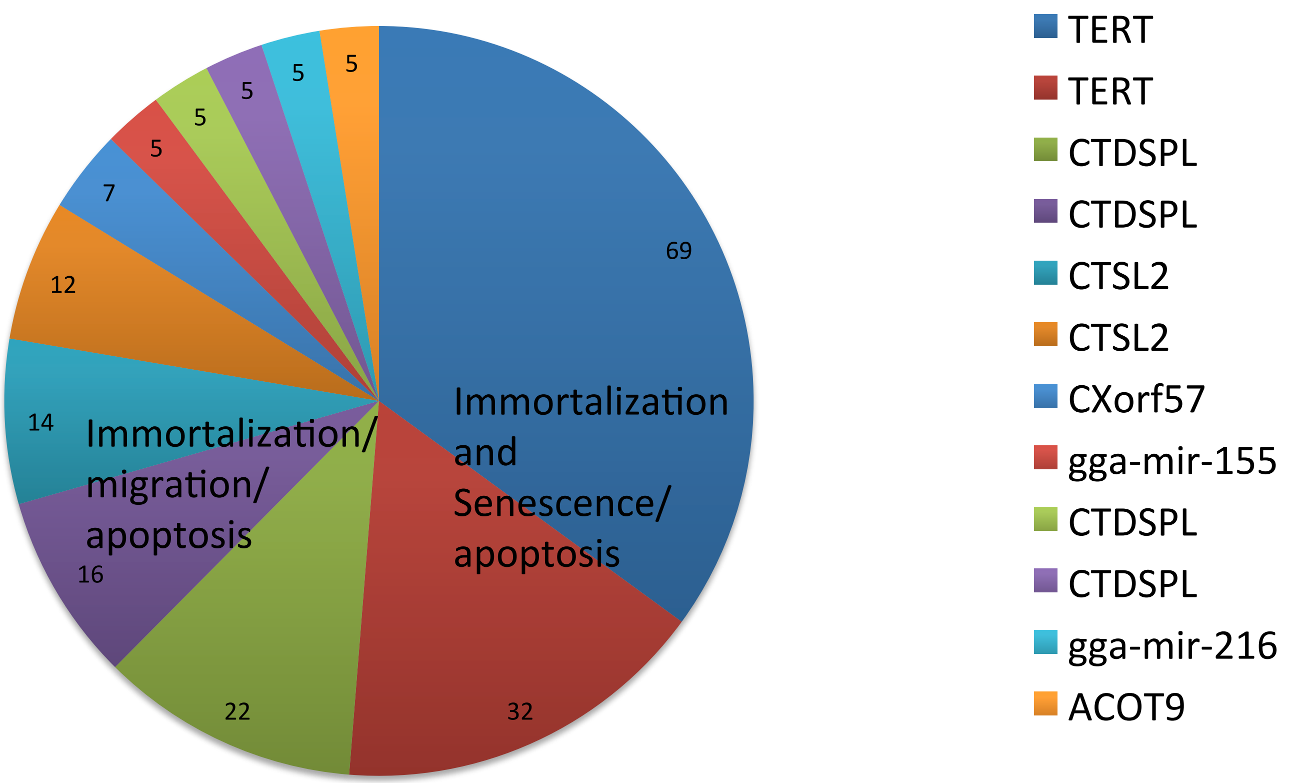

69
32
22
16
14
12
7
5
5
5
5
5
Immortalization and Senescence/ apoptosis
Immortalization/ migration/ apoptosis
TERT
TERT
CTDSPL
CTDSPL
CTSL2
CTSL2
CXorf57
gga-mir-155
CTDSPL
CTDSPL
gga-mir-216
ACOT9

F6L Tumor

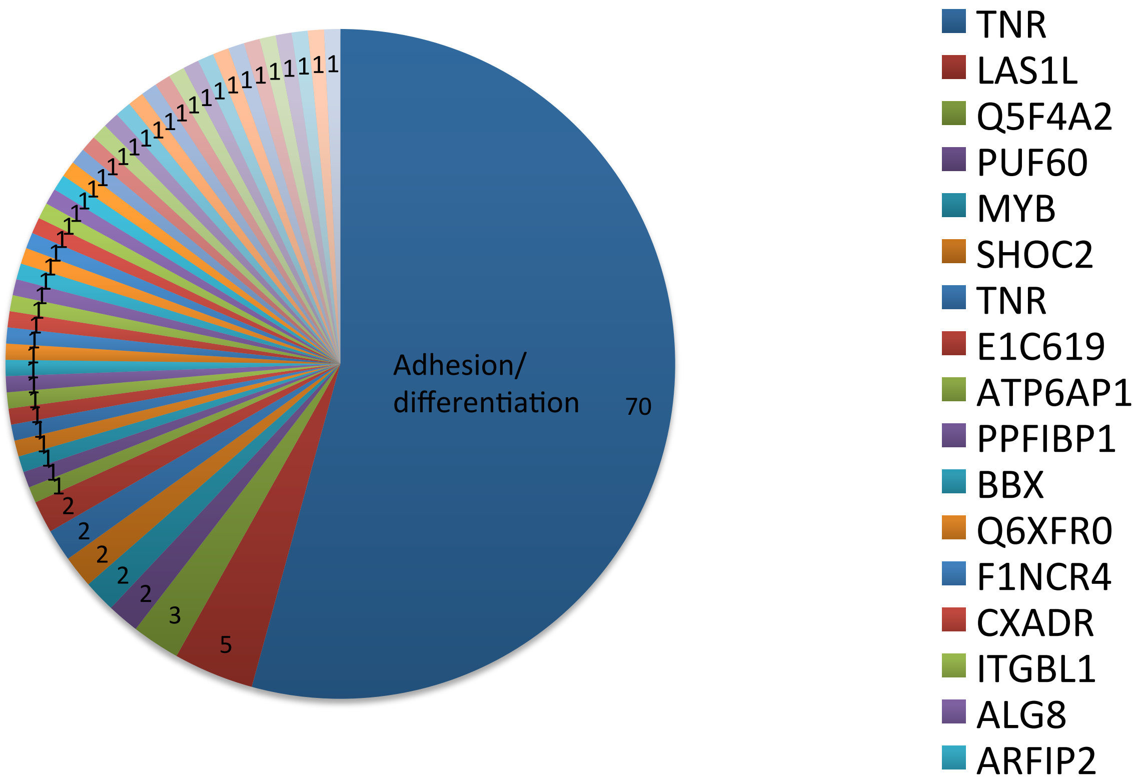
Adhesion/
differentiation
70
5
3
2
2
2
2
2
1
TNR
LAS1L
Q5F4A2
PUF60
MYB
SHOC2
TNR
E1C619
ATP6AP1
PPFIBP1
BBX
Q6XFR0
F1NCR4
CXADR
ITGBL1
ALG8
ARFIP2

F7L Tumor

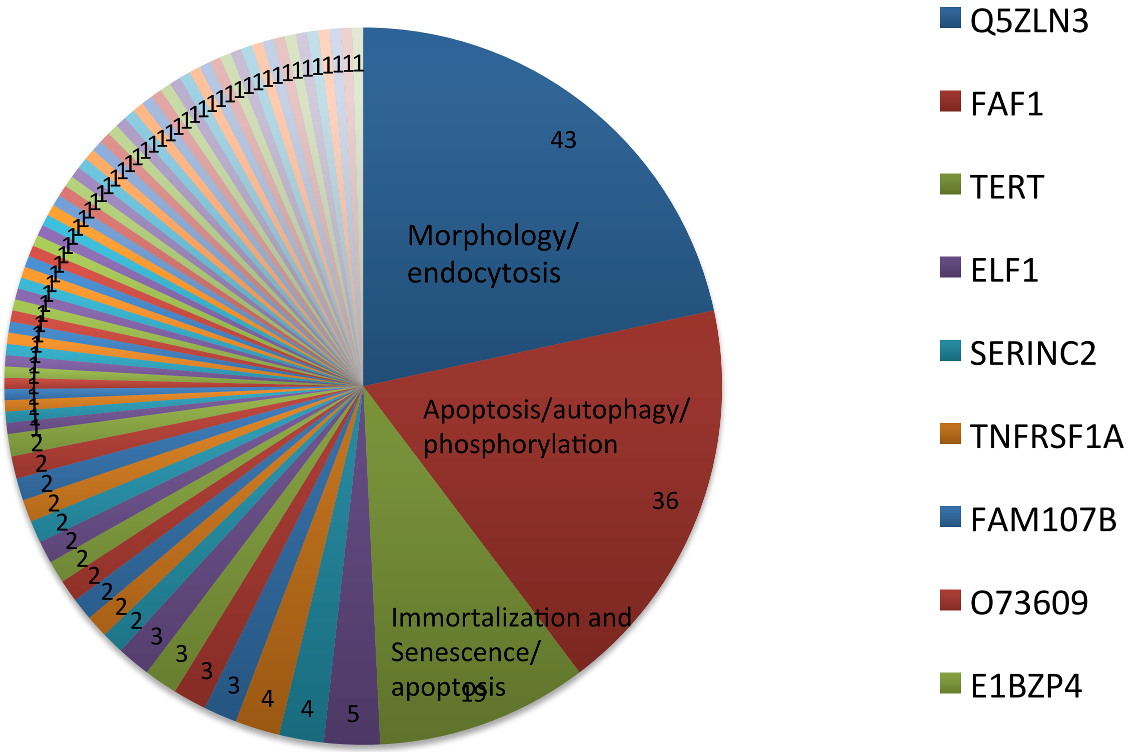
43
Morphology/
endocytosis
Apoptosis/autophagy/
phosphorylation
36
Immortalization and
Senescence/
apoptosis
5
4
4
3
3
3
3
Q5ZLN3
FAF1
TERT
ELF1
SERINC2
TNFRSF1A
FAM107B
O73609
E1BZP4

E1L Tumor

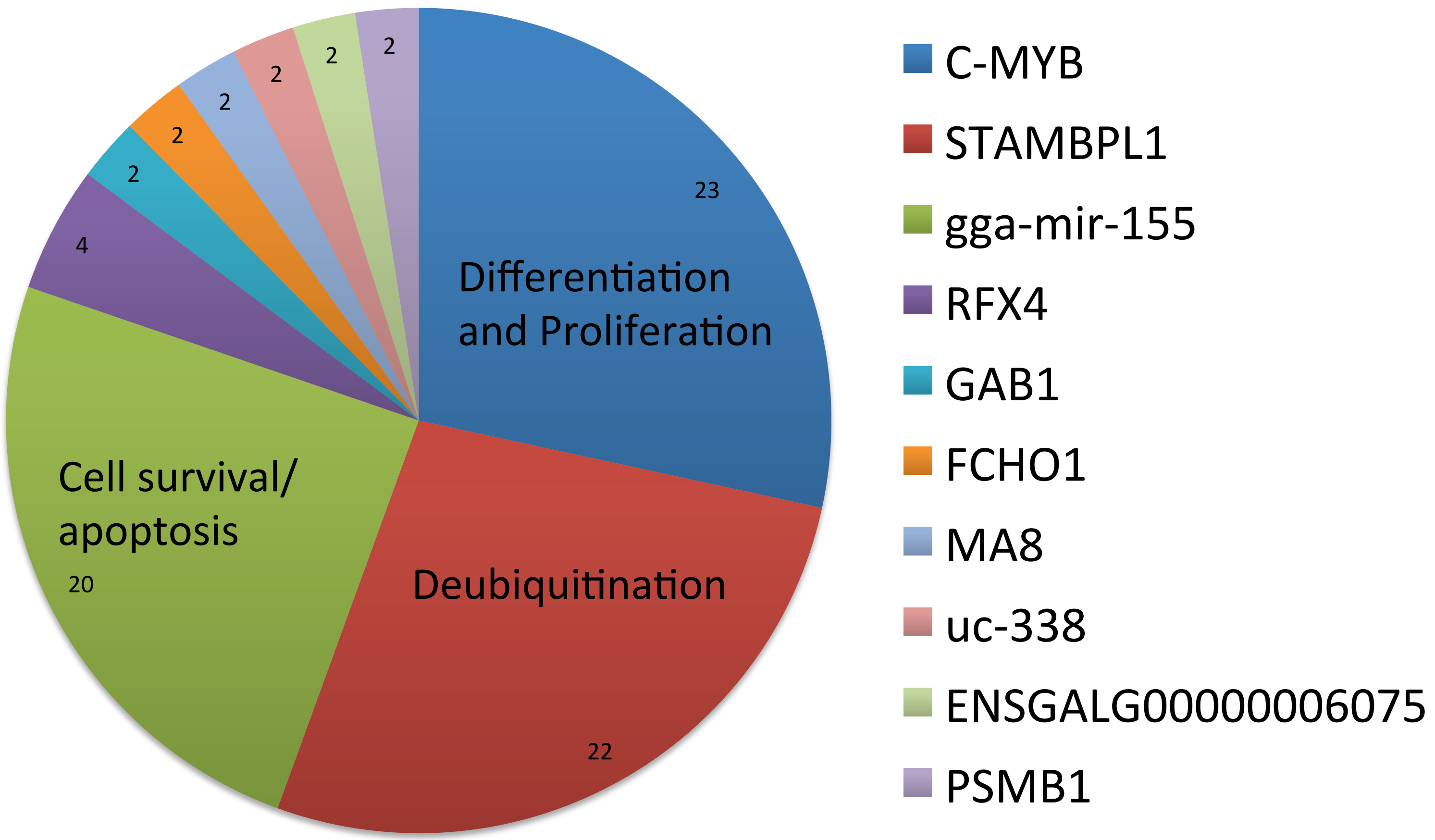
2
2
2
2
2
2
23
4
Differentiation and Proliferation
Cell survival/ apoptosis
20
Deubiquitination
22
C-MYB
STAMBPL1
gga-mir-155
RFX4
GAB1
FCHO1
MA8
uc-338
ENSGALG00000006075
PSMB1

E2L Tumor

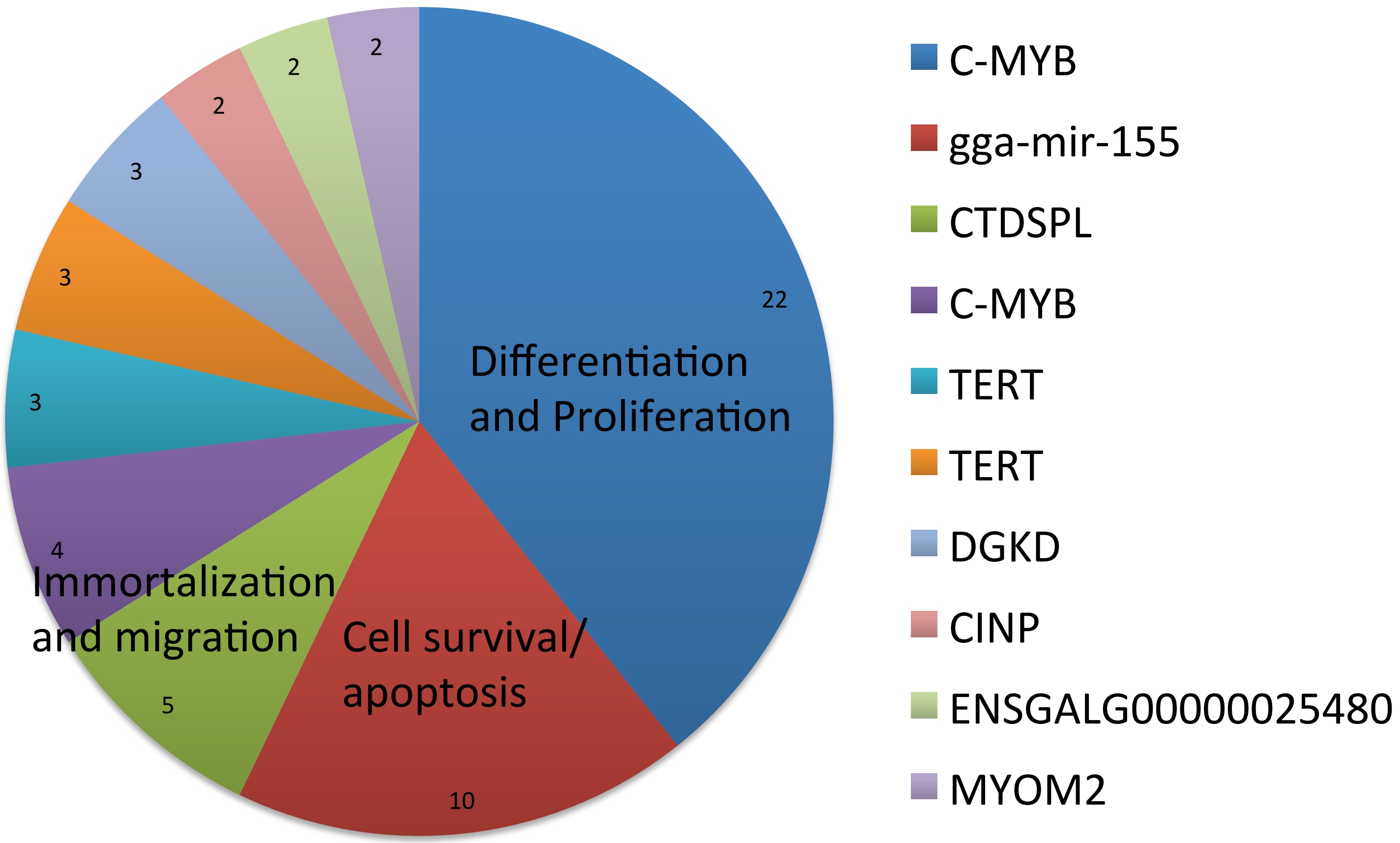
2
2
2
3
3
3
22
Differentiation and Proliferation
4
Immortalization and migration
Cell survival/ apoptosis
5
10
C-MYB
gga-mir-155
CTDSPL
C-MYB
TERT
TERT
DGKD
CINP
ENSGALG00000025480
MYOM2

E3L Tumor

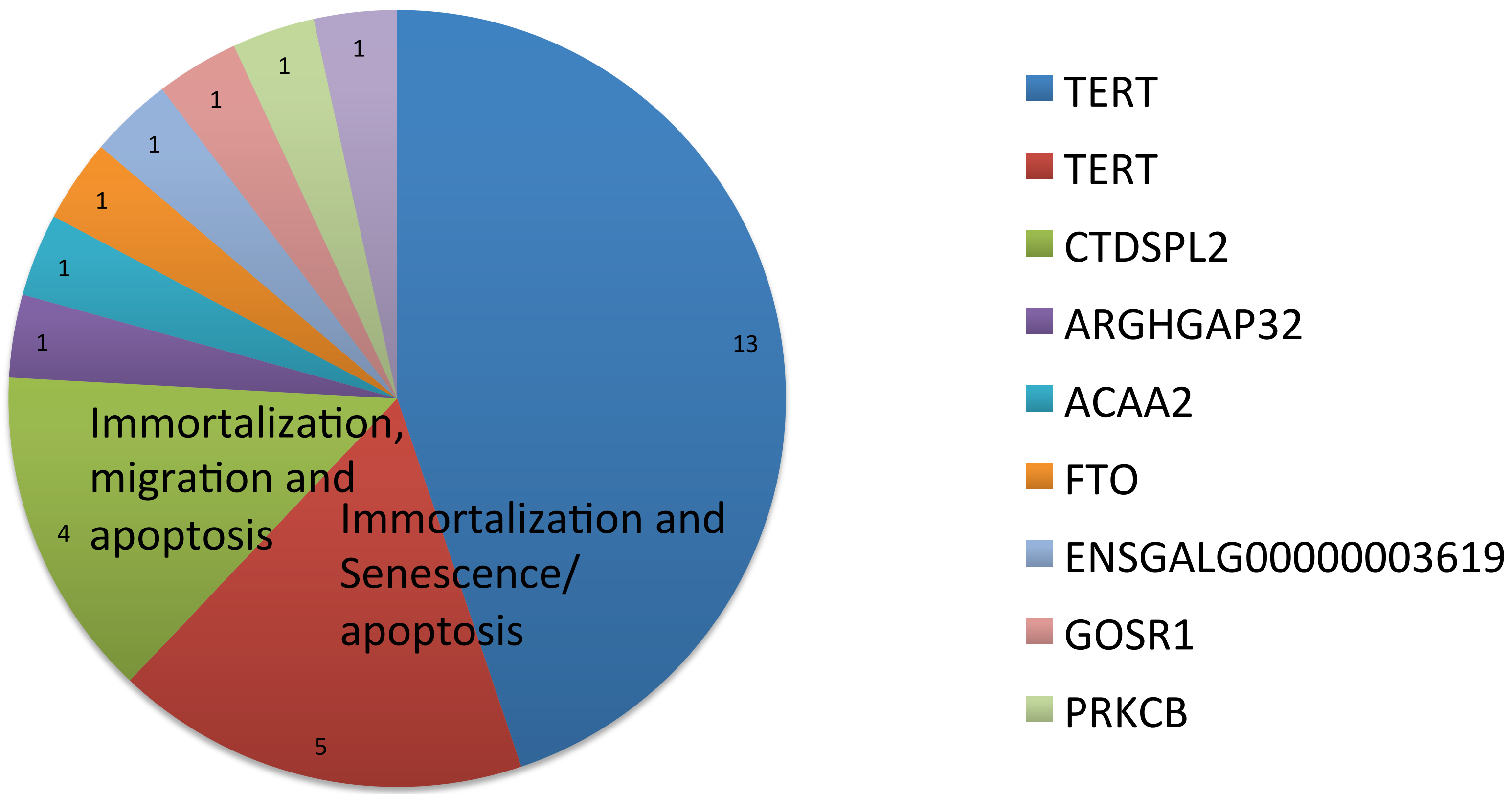
1
1
1
1
1
1
1
13
Immortalization, migration and apoptosis
4
Immortalization and Senescence/ apoptosis
5
TERT
TERT
CTDSPL2
ARGHGAP32
ACAA2
FTO
ENSGALG00000003619
GOSR1
PRKCB

E4L Tumor

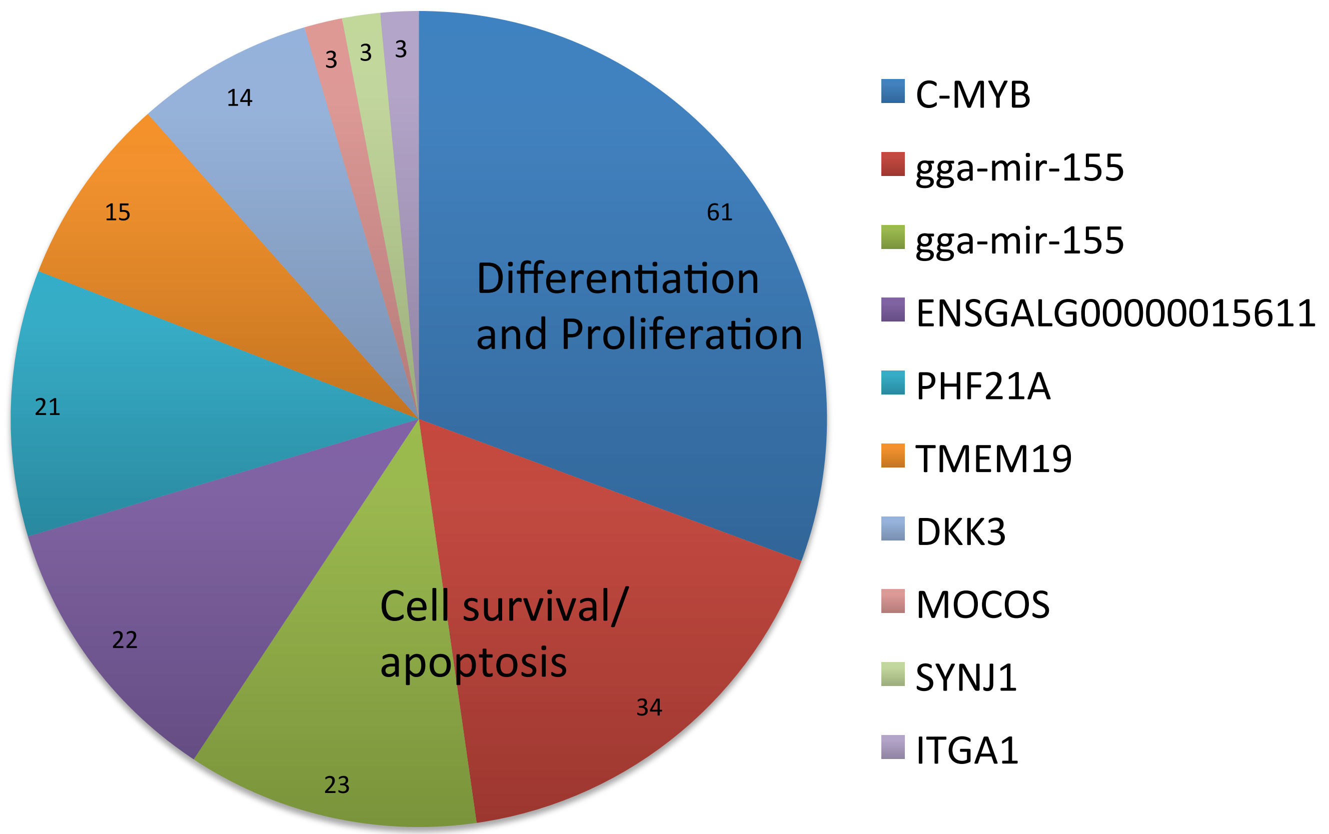
61
34
23
22
21
15
14
3
3
3
Differentiation and Proliferation
Cell survival/ apoptosis
C-MYB
gga-mir-155
gga-mir-155
ENSGALG00000015611
PHF21A
TMEM19
DKK3
MOCOS
SYNJ1
ITGA1

Spleen

A2S Tumor

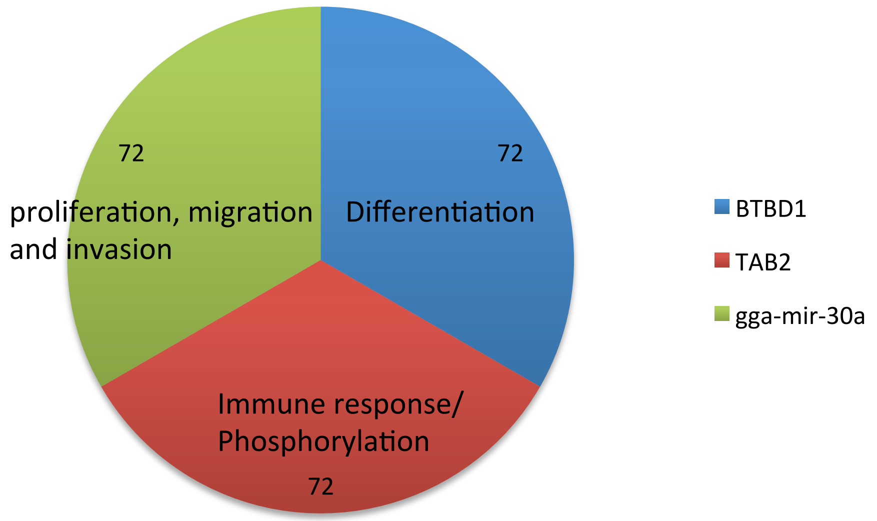
72
72
proliferation, migration and invasion
Differentiation
Immune response/ Phosphorylation
72
BTBD1
TAB2
gga-mir-30a

A6S Tumor

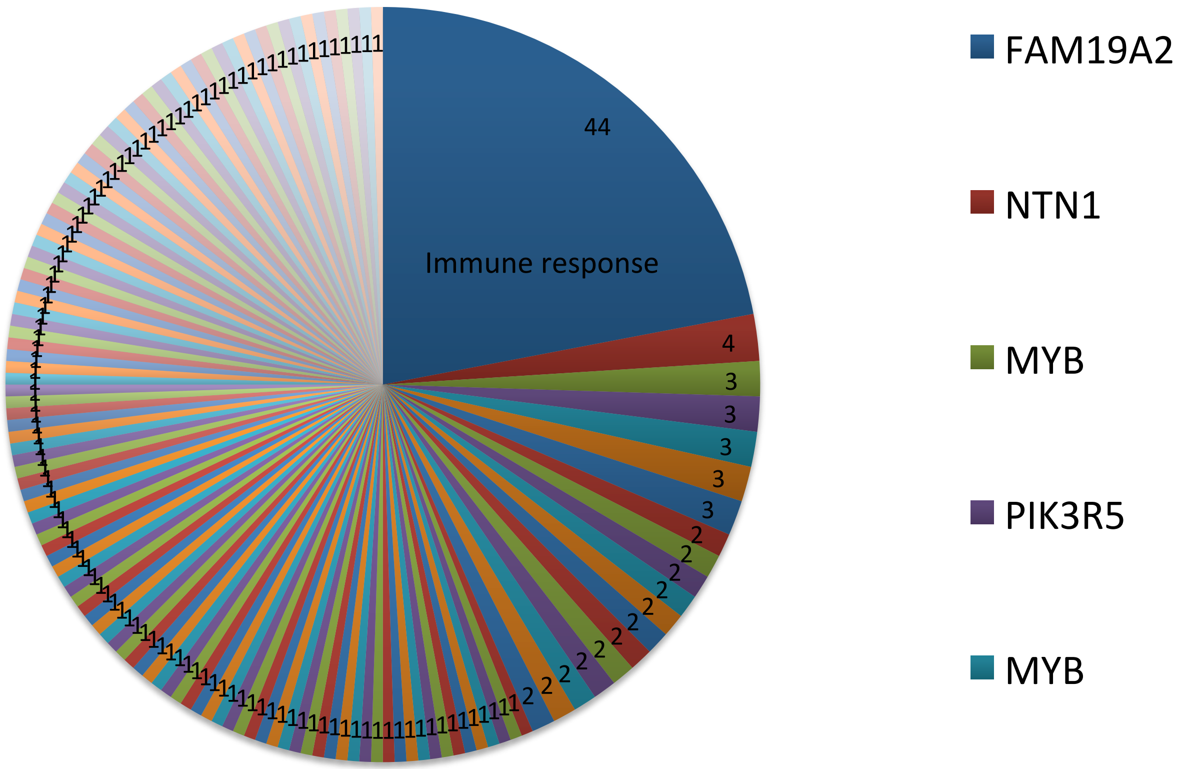

C2S Tumor

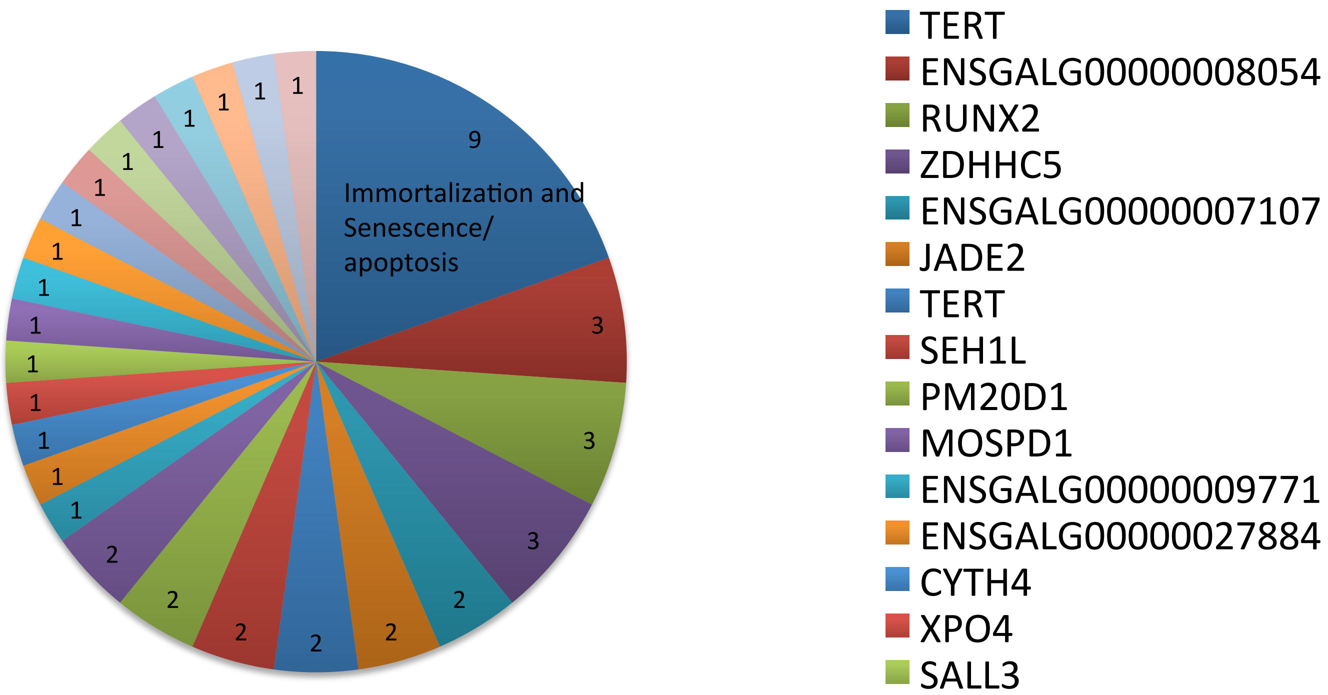

9
Immortalization and Senescence/ apoptosis
3
3
3
2
2
2
2
2
2
1
1
1
1
1
1
1
1
1
1
1
1
1
1
1
TERT
ENSGALG00000008054
RUNX2
ZDHHC5
ENSGALG00000007107
JADE2
TERT
SEH1L
PM20D1
MOSPD1
ENSGALG00000009771
ENSGALG00000027884
CYTH4
XPO4
SALL3

C4S Tumor

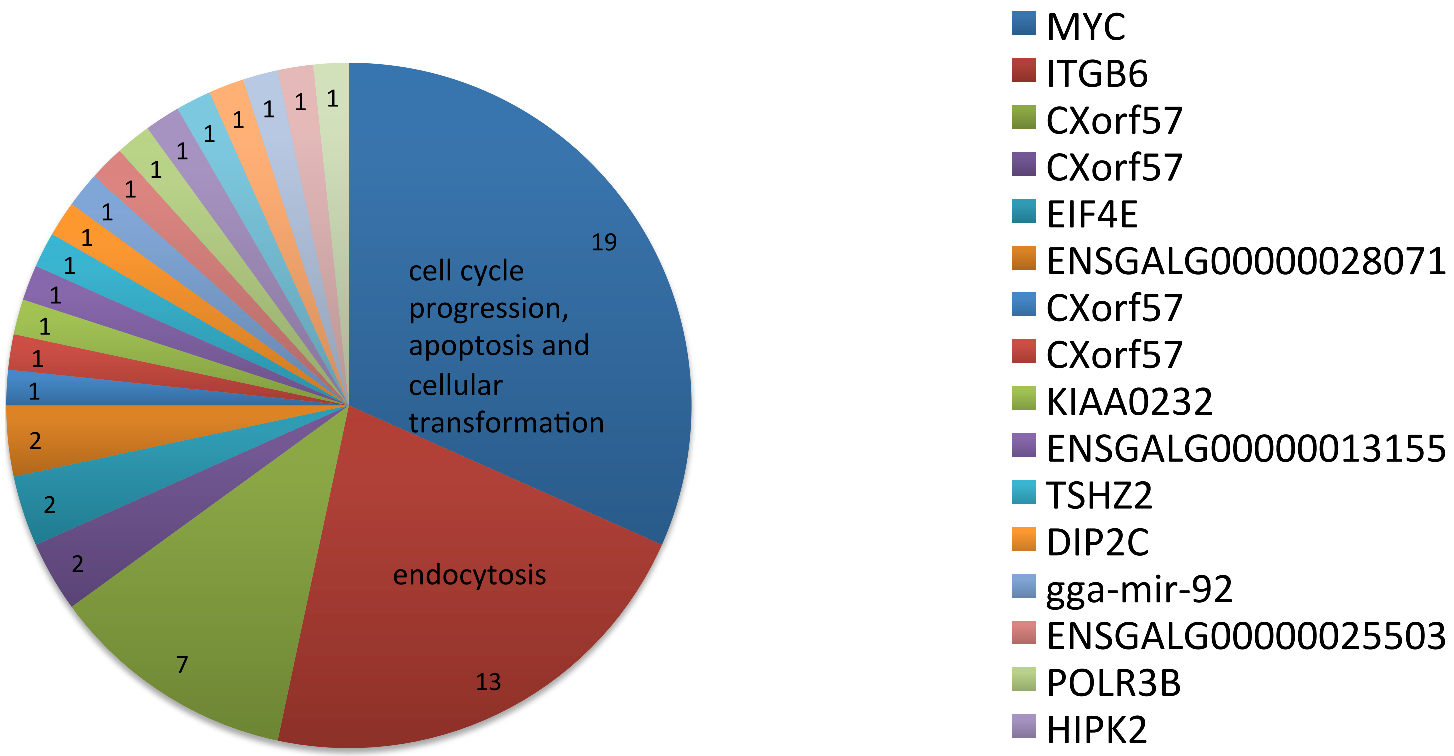

19
cell cycle progression, apoptosis and cellular transformation
endocytosis
13
7
2
2
2
1
1
1
1
1
1
1
1
1
1
1
1
1
1
MYC
ITGB6
CXorf57
CXorf57
EIF4E
ENSGALG00000028071
CXorf57
CXorf57
KIAA0232
ENSGALG00000013155
TSHZ2
DIP2C
gga-mir-92
ENSGALG00000025503
POLR3B
HIPK2

D5S Tumor

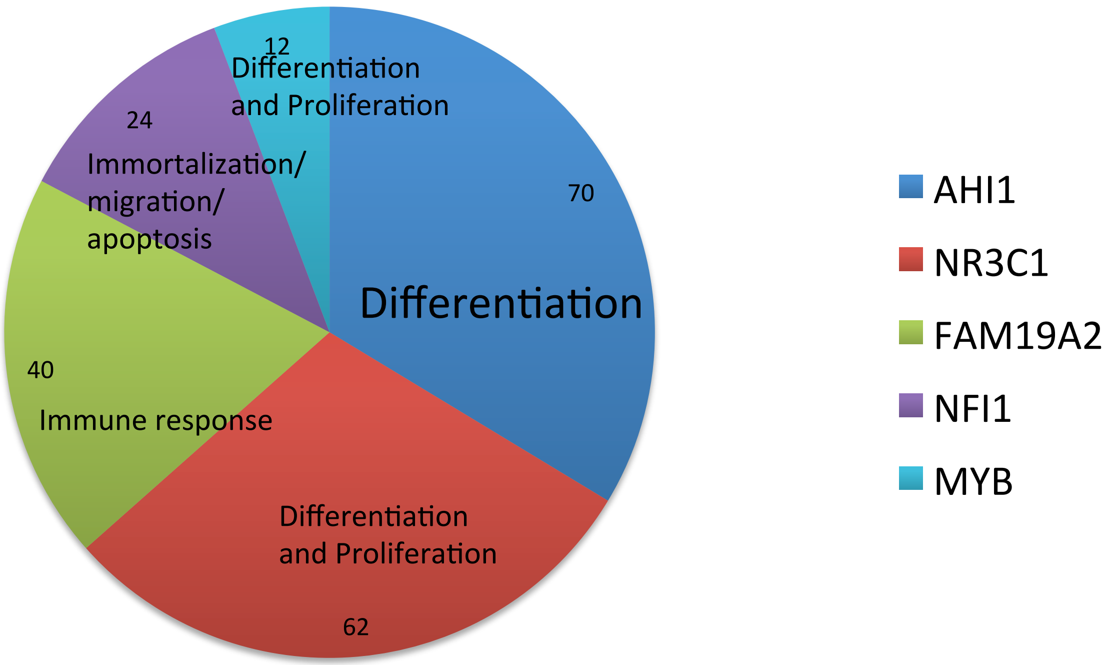

12
Differentiation
and Proliferation
24
Immortalization/
migration/
apoptosis
70
Differentiation
40
Immune response
Differentiation
and Proliferation
62
AHI1
NR3C1
FAM19A2
NFI1
MYB

F8S Tumor

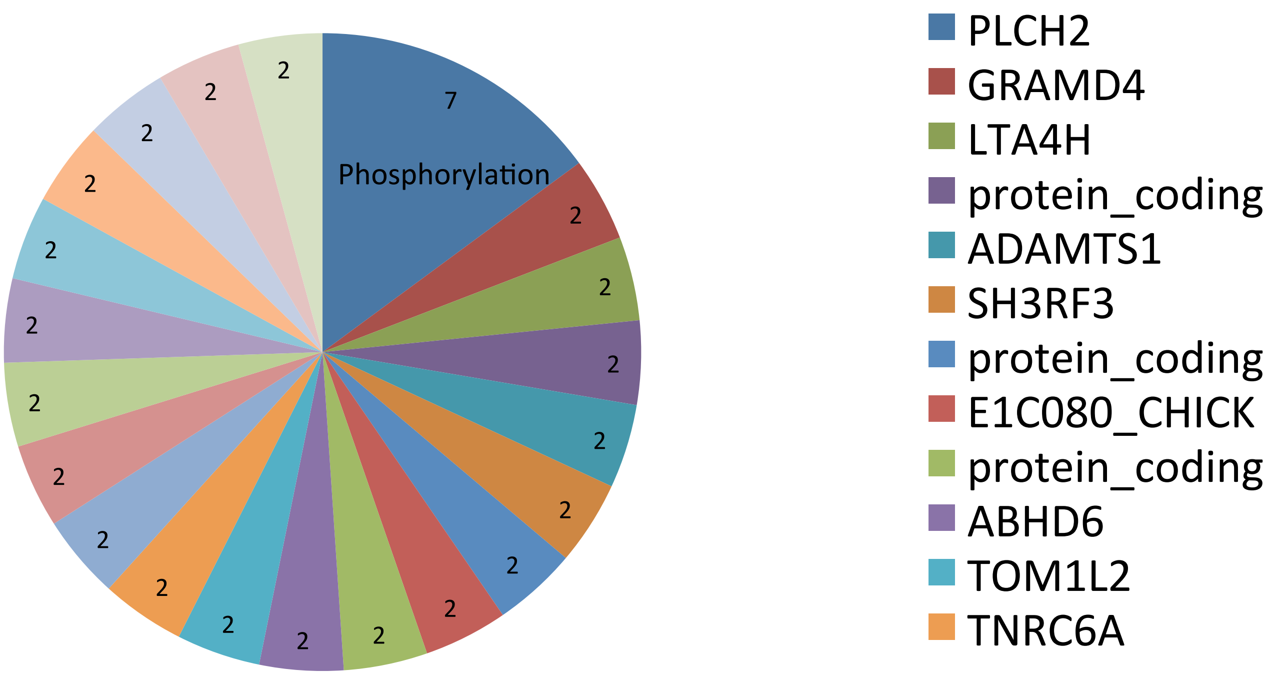
7
Phosphorylation
2
PLCH2
GRAMD4
LTA4H
protein_coding
ADAMTS1
SH3RF3
protein_coding
E1C080_CHICK
protein_coding
ABHD6
TOM1L2
TNRC6A

Kidney

A2K Tumor

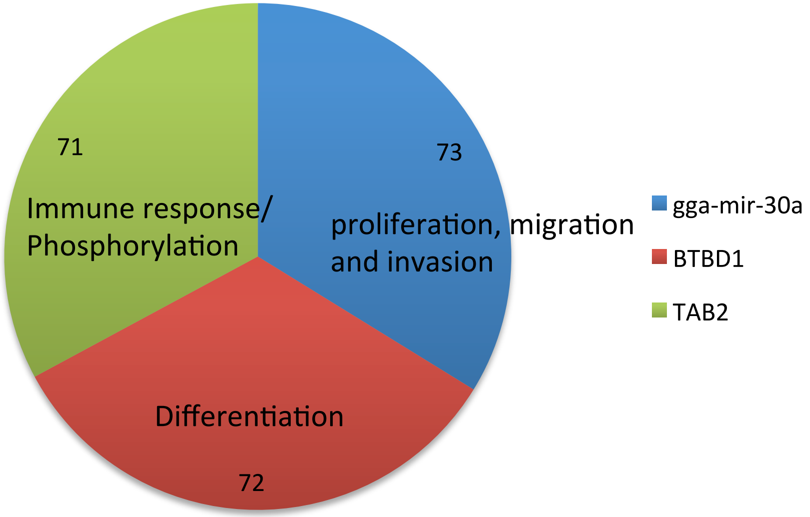

71
73
Immune response/
Phosphorylation
proliferation, migration
and invasion
Differentiation
72
gga-mir-30a
BTBD1
TAB2

C3K Tumor

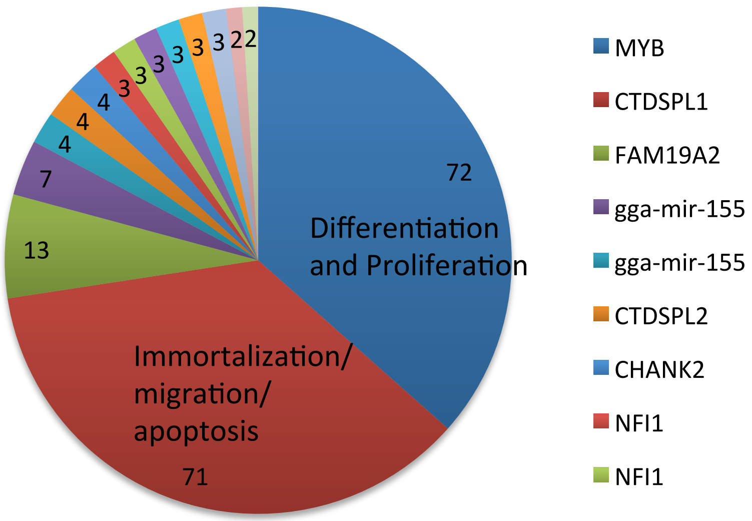
72
Differentiation and Proliferation
Immortalization/ migration/ apoptosis
71
13
7
4
4
4
3
3
3
3
3
3
2
2
MYB
CTDSPL1
FAM19A2
gga-mir-155
gga-mir-155
CTDSPL2
CHANK2
NFI1
NFI1

C7K Tumor

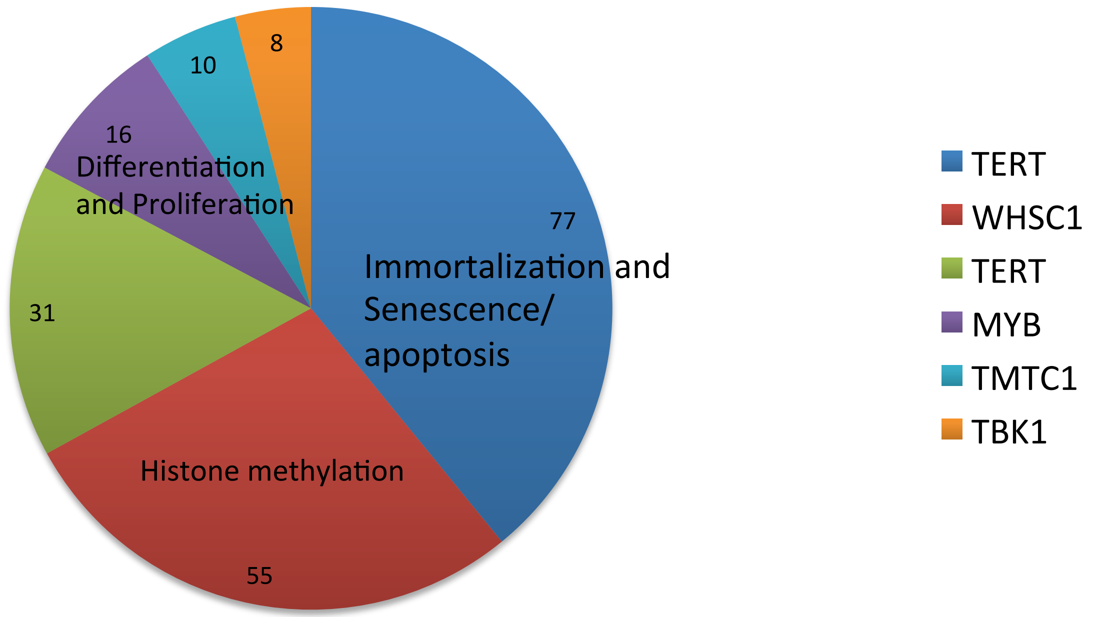
8
10
16
Differentiation and Proliferation
77
Immortalization and Senescence/ apoptosis
31
Histone methylation
55
TERT
WHSC1
TERT
MYB
TMTC1
TBK1

C8K Tumor

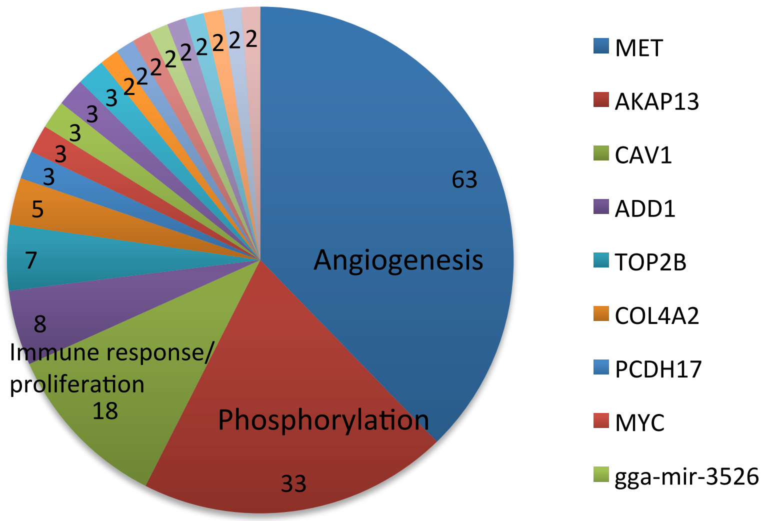
63
Angiogenesis
33
Phosphorylation
Immune response/
proliferation
18
8
7
5
3
3
3
3
3
2
2
2
2
2
2
2
2
2
MET
AKAP13
CAV1
ADD1
TOP2B
COL4A2
PCDH17
MYC
gga-mir-3526

D2K Tumor

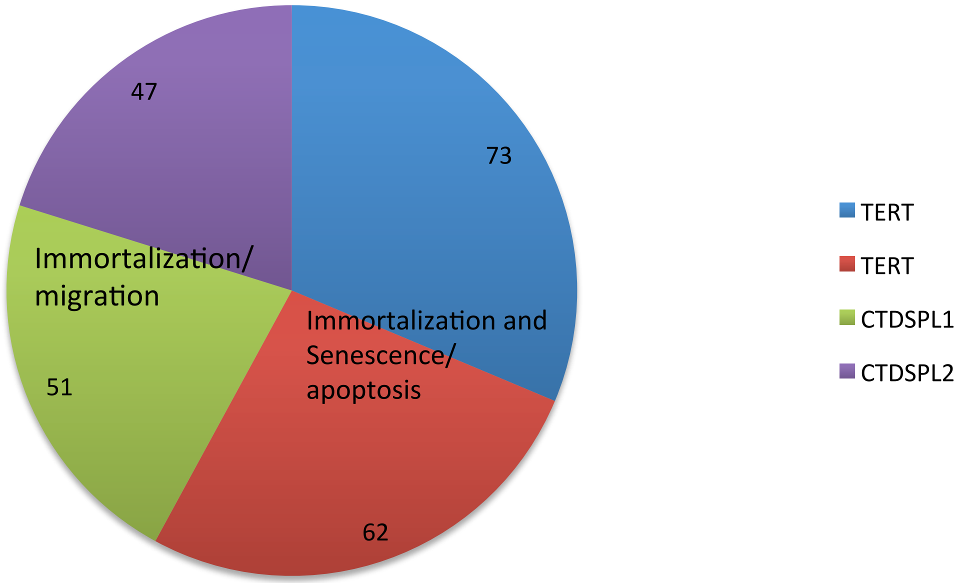
47
73
Immortalization/
migration
Immortalization and
Senescence/
apoptosis
51
62
TERT
TERT
CTDSPL1
CTDSPL2

D5K Tumor

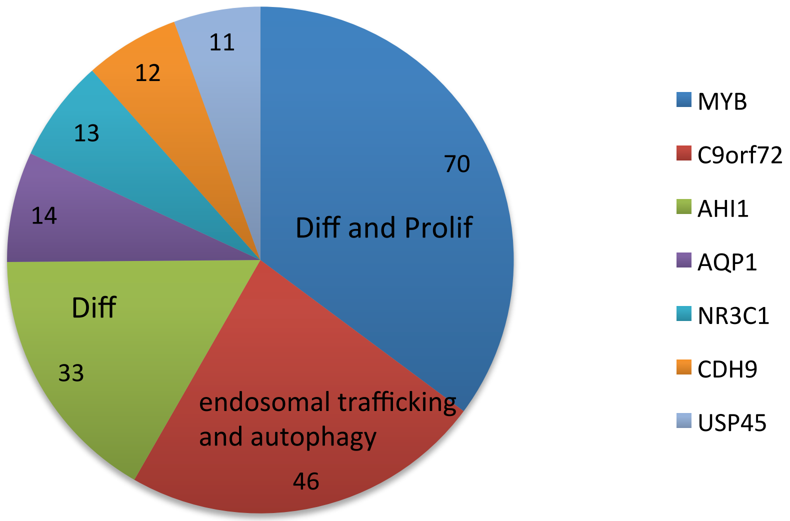

11
12
13
70
14
Diff and Prolif
Diff
33
endosomal trafficking and autophagy
46
MYB
C9orf72
AHI1
AQP1
NR3C1
CDH9
USP45

C4K Tumor

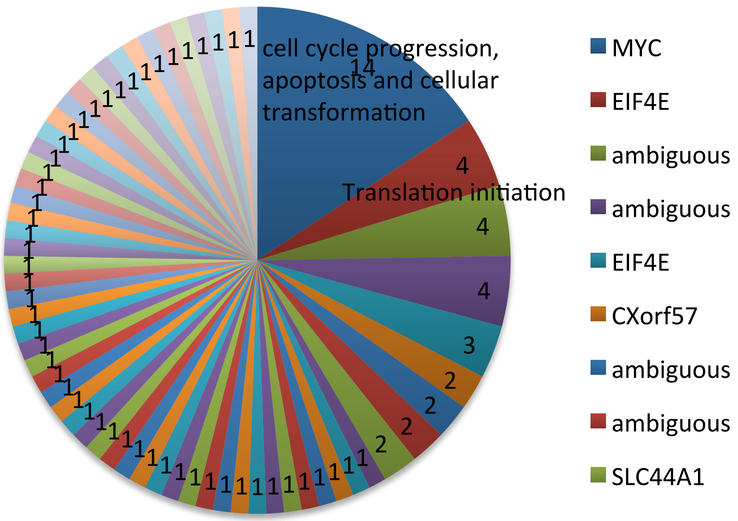

cell cycle progression, apoptosis and cellular transformation
14
Translation initiation
4
4
4
3
2
2
2
2
MYC
EIF4E
ambiguous
ambiguous
EIF4E
CXorf57
ambiguous
ambiguous
SLC44A1
