# Supplementary material for: Selection for avian leukosis virus integration sites determines the clonal progression of B-cell lymphomas
Source: PLoS Pathog. 2017 Nov 3;13(11):e1006708. doi: 10.1371/journal.ppat.1006708 (PMC5687753; doi:10.1371/journal.ppat.1006708)
Supplement: S5 Fig — The top 200 breakpoints of clonally expanded unique integration sites are depicted within each pie chart, along with the list of most proximal host genes. (PDF) [file ppat.1006708.s005.pdf]

Bird C2

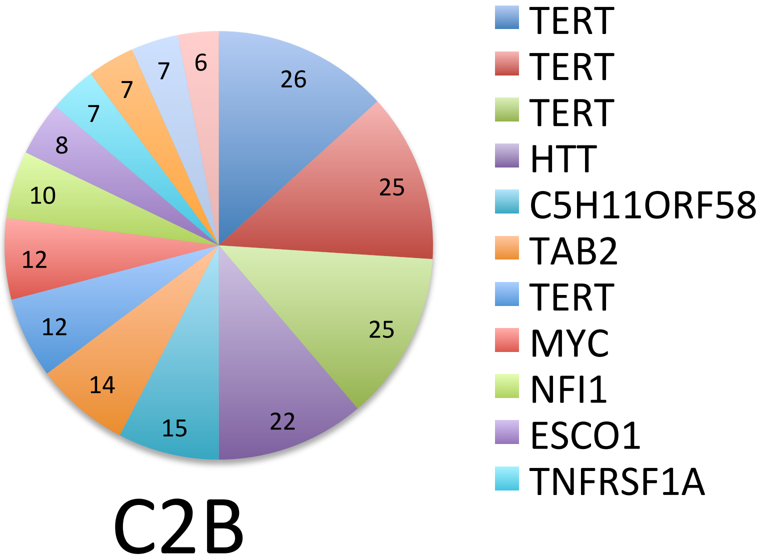

C2B

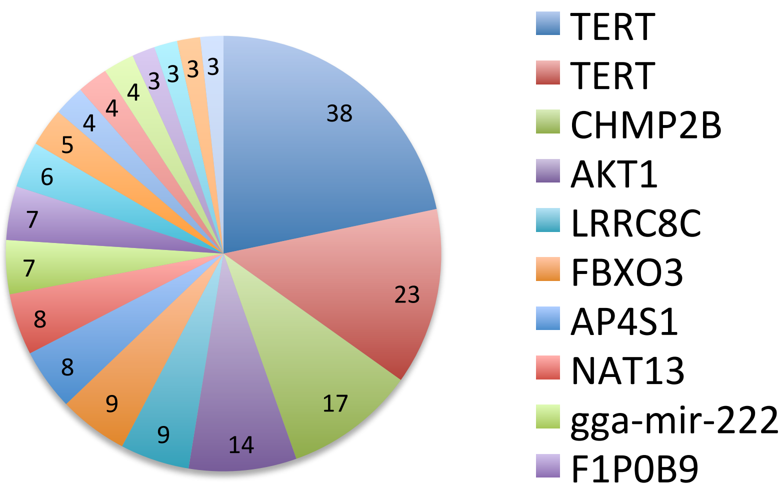

C2L

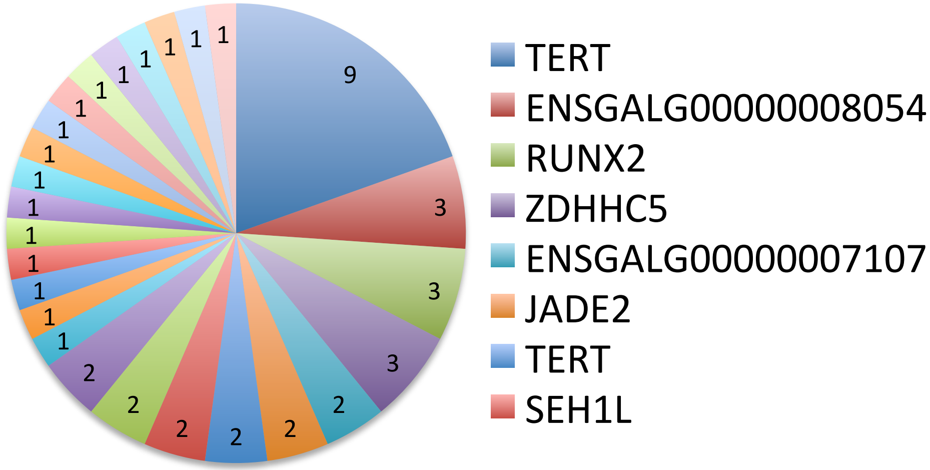

C2S

Bird C7

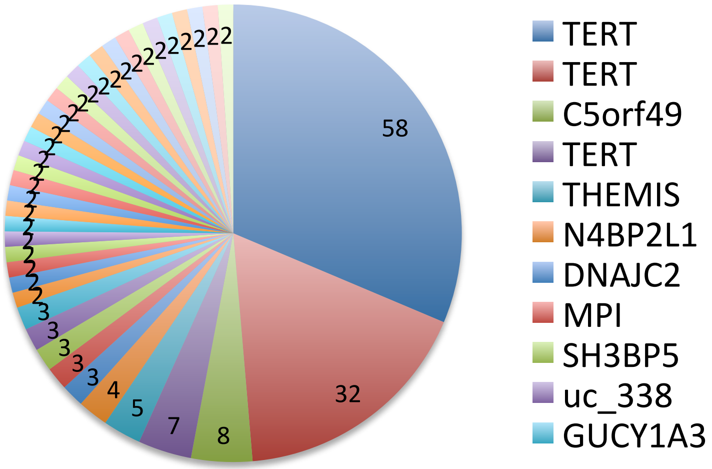

C7B

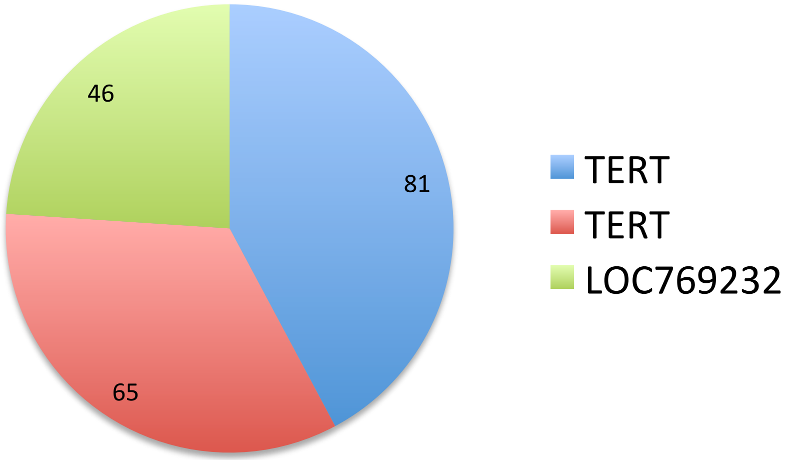

C7L

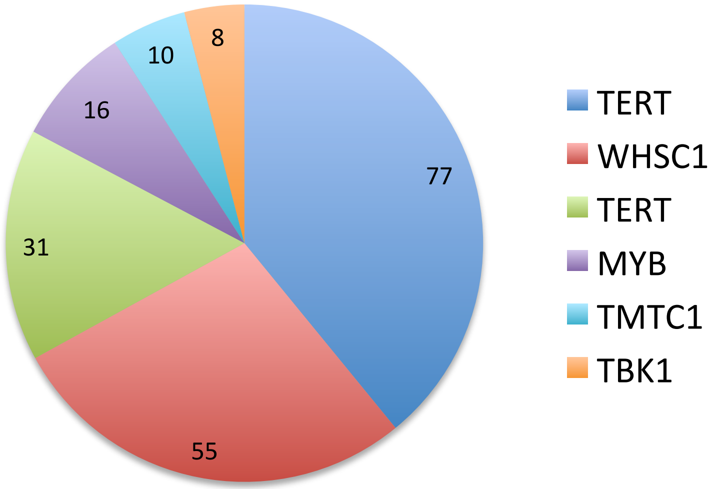

C7K

Bird D2

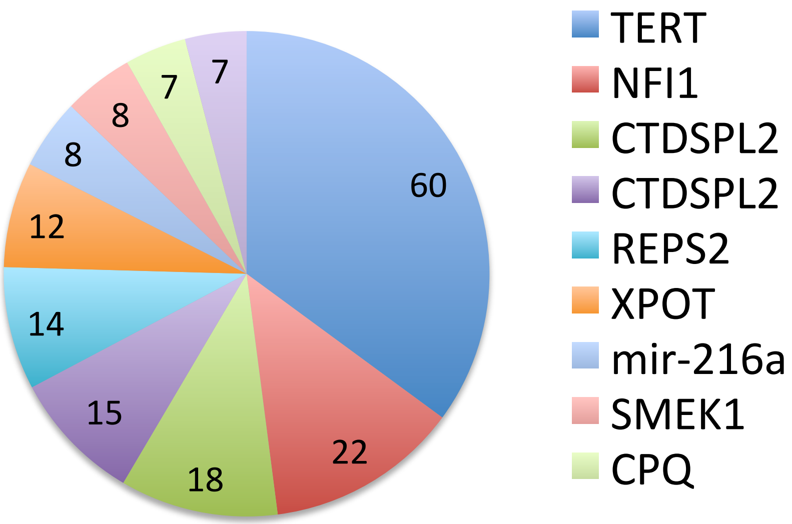

D2B

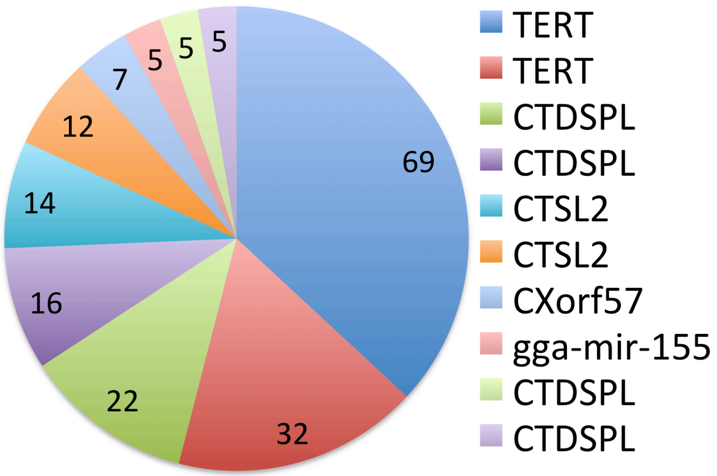

D2L

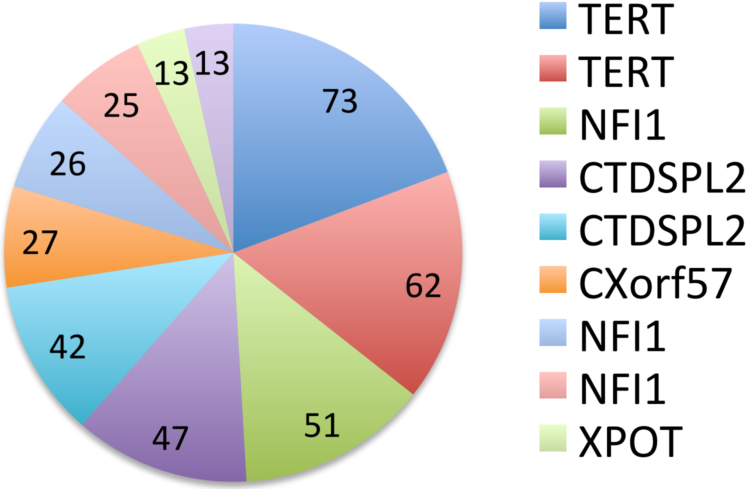

D2K
