# Supplementary material for: Selection for avian leukosis virus integration sites determines the clonal progression of B-cell lymphomas
Source: PLoS Pathog. 2017 Nov 3;13(11):e1006708. doi: 10.1371/journal.ppat.1006708 (PMC5687753; doi:10.1371/journal.ppat.1006708)
Supplement: S6 Fig — Top 10 most clonally expanded integrations from different slices of primary tumor (C2B) and liver metastases (C6L, C7L and D2L) are illustrated. Individual pie charts represent UISs and corresponding extent of clonal expansion (as breakpoints) from a different slice of the tissue. Slices were chosen randomly from three distinct portions of tumor mass, including peripheral and interior regions. (PDF) [file ppat.1006708.s006.pdf]

C2B

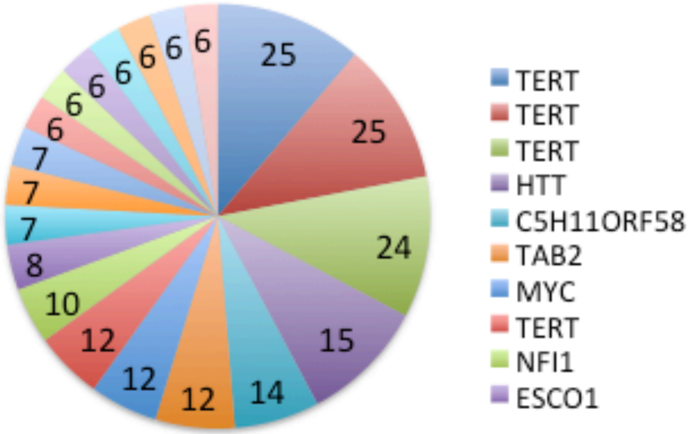
25
25
24
15
14
12
12
12
10
8
7
7
7
6
6
6
6
6
6
6
TERT
TERT
TERT
HTT
C5H11ORF58
TAB2
MYC
TERT
NFI1
ESCO1

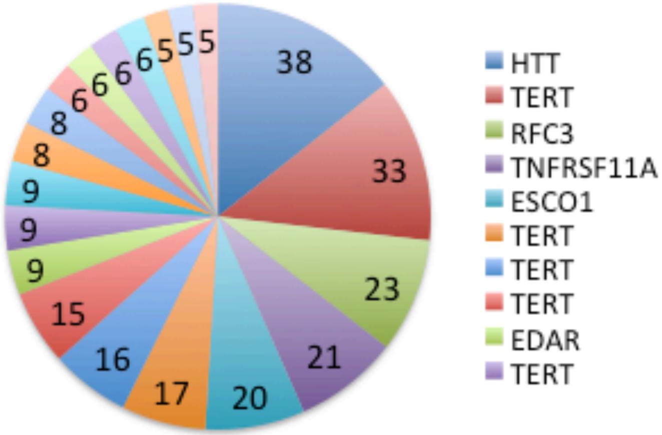
38
33
23
21
20
17
16
15
9
9
9
8
8
6
6
6
6
5
5
5
HTT
TERT
RFC3
TNFRSF11A
ESCO1
TERT
TERT
TERT
EDAR
TERT

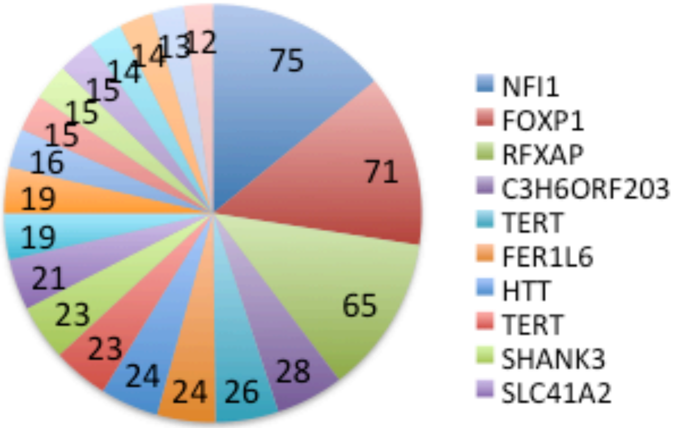
75
71
65
28
26
24
24
23
23
21
19
19
16
15
15
15
14
14
13
12
NFI1
FOXP1
RFXAP
C3H6ORF203
TERT
FER1L6
HTT
TERT
SHANK3
SLC41A2

C6L

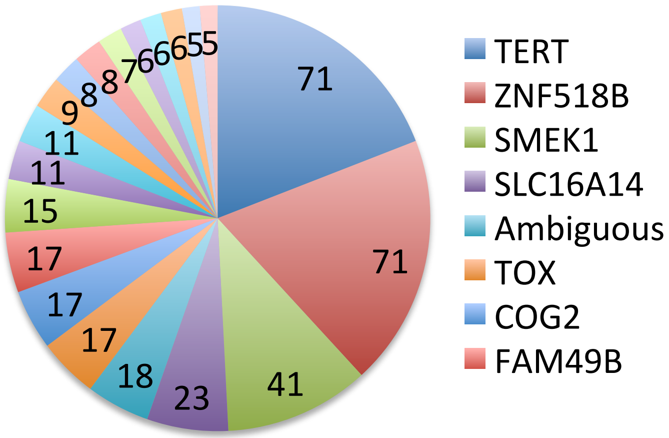
71
71
41
23
18
17
17
17
15
11
11
TERT
ZNF518B
SMEK1
SLC16A14
Ambiguous
TOX
COG2
FAM49B

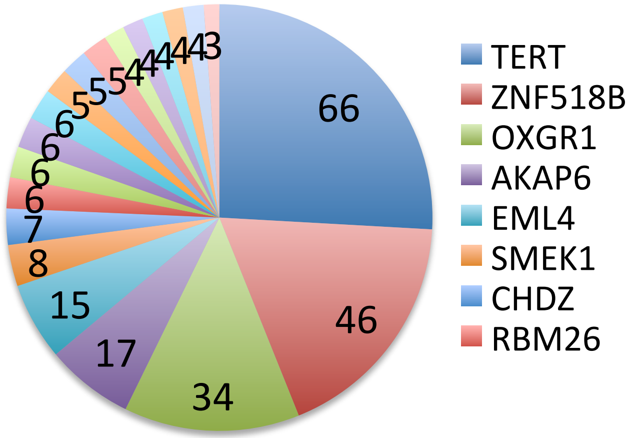
66
46
34
17
15
8
7
TERT
ZNF518B
OXGR1
AKAP6
EML4
SMEK1
CHDZ
RBM26

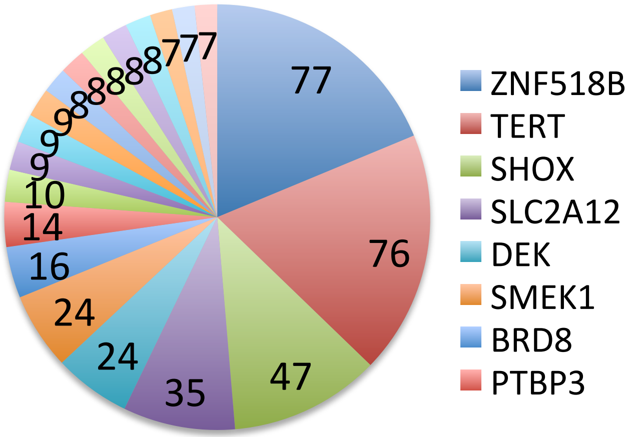
77
76
47
35
24
24
16
14
10
ZNF518B
TERT
SHOX
SLC2A12
DEK
SMEK1
BRD8
PTBP3

C7L

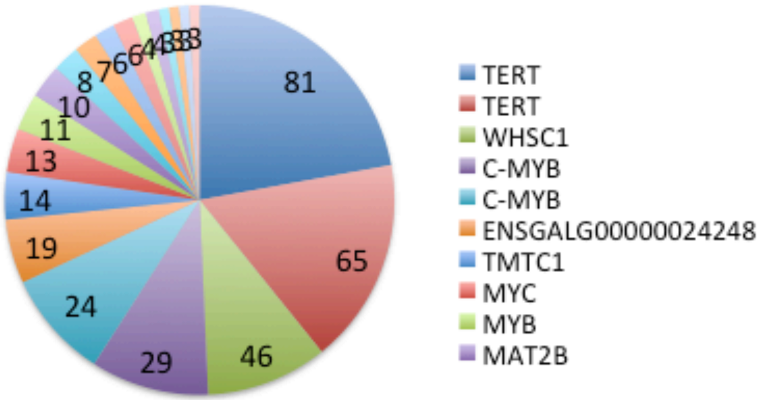
81
65
46
29
24
19
14
13
11
10
8
7
TERT
TERT
WHSC1
C-MYB
C-MYB
ENSGALG00000024248
TMTC1
MYC
MYB
MAT2B

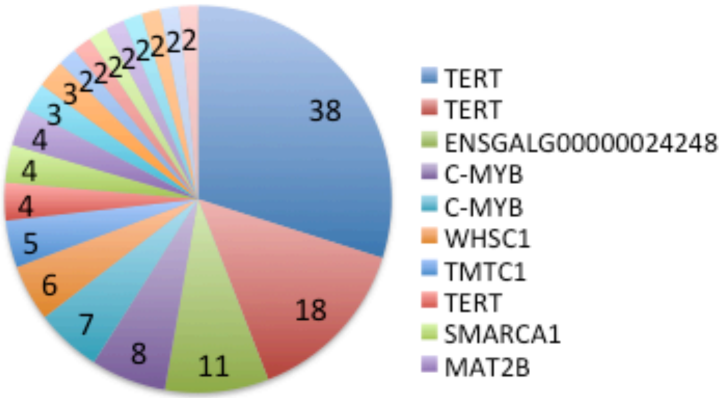
38
18
11
8
7
6
5
4
4
4
3
3
TERT
TERT
ENSGALG00000024248
C-MYB
C-MYB
WHSC1
TMTC1
TERT
SMARCA1
MAT2B

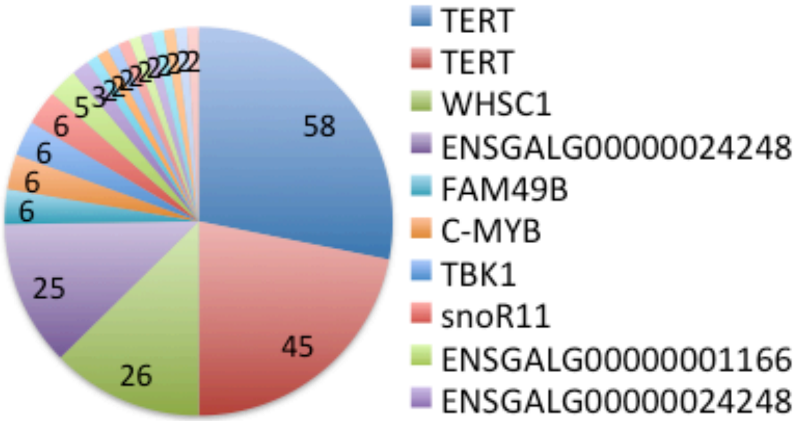
58
45
26
25
6
6
6
6
5
TERT
TERT
WHSC1
ENSGALG00000024248
FAM49B
C-MYB
TBK1
snoR11
ENSGALG00000001166
ENSGALG00000024248
