# Supplementary material for: Selection for avian leukosis virus integration sites determines the clonal progression of B-cell lymphomas
Source: PLoS Pathog. 2017 Nov 3;13(11):e1006708. doi: 10.1371/journal.ppat.1006708 (PMC5687753; doi:10.1371/journal.ppat.1006708)
Supplement: S1 Table — The different ALV subgroups (A, C and J) and cell types (CEF, DT40 and HeLa) used for analysis are denoted. (PDF) [file ppat.1006708.s008.pdf]

Sample	ALV-A CEF	ALV-C CEF	ALV-J CEF	ALV-C DT-40	ALV-A Tumors	ALV-C HeLa
#Integrations	2125	4313	3548	4377	71368	1053
#Matched Random Sites	2125	4313	3548	4377	71368	1053
