# Supplementary material for: Selection for avian leukosis virus integration sites determines the clonal progression of B-cell lymphomas
Source: PLoS Pathog. 2017 Nov 3;13(11):e1006708. doi: 10.1371/journal.ppat.1006708 (PMC5687753; doi:10.1371/journal.ppat.1006708)
Supplement: S2 Table — (PDF) [file ppat.1006708.s009.pdf]

Type	Tissue	#Samples
Uninfected	Bursa	2
Uninfected	Liver	1
Non-tumor	Brain	2
Non-tumor	Kidney	1
Inflammation	Liver	2
Neoplastic follicle	Bursa	9
Primary Tumor	Bursa	25
Metastases	Liver	13
Metastases	Kidney	10
Metastases	Spleen	7
