# Supplementary material for: Selection for avian leukosis virus integration sites determines the clonal progression of B-cell lymphomas
Source: PLoS Pathog. 2017 Nov 3;13(11):e1006708. doi: 10.1371/journal.ppat.1006708 (PMC5687753; doi:10.1371/journal.ppat.1006708)
Supplement: S4 Table — (PDF) [file ppat.1006708.s011.pdf]

**Pathway**	**TERT Cooperating players (15 tumors, 9 birds)**	**MYB Cooperating players (15 tumors, 11 birds)**
Differentiation	MYB (A1,B4,A5,C7), SELIL (B4), HTT (C2), ZNF518B (C6), AHI1 (A5)	LMO4 (A4), AHI1 (A5,D5), SELIL (B4), NR3C1 (D5)
Phosphorylation	SMEK1 (C6), CCNA2 (A1), TAB2 (C2), AKT1 (C2)	CCNA2 (A1), DCAKD (E5)
Proliferation	MYB (A1,B4,A5,C7), EGFR (C6), AKT1 (C2)	NR3C1 (D5), HELLS (E5)
Cell survival/apoptosis	EGFR (C6), CTDSPL2 (D2,E3), AKT1 (C2)	TERT (A1,A5,B4,C7), KIF1B (A4), MYC (E5), mir-155 (E1,E2,E4)
DNA Repair	CCNA2 (A1), SMEK1 (C6)	CCNA2 (A1), HELLS (E5)
Immune Response	TPN (A1), TAB2 (C2),	TPN (A1), FAM19A2 (D5)
Immortalization	CTDSPL (D2), CTDSPL2 (D2,E3)	TERT (A1,A5,B4,C7,E2), CTDSPL (C3,D5,E2)
